# Reproducibility Before Treatment Decision Rules: A Targeted Scoping Review of AI-Assisted Coronary Plaque Characterization

**DOI:** 10.3390/diagnostics16213703

**Published:** 2026-11-09

**Authors:** Mingyang Qin, Negin Moghadasi, Ahmadreza Ghasemiesfe, Misagh Piran

**Affiliations:** 1 CardiaX Intelligence LLC, Bellevue, WA 98006, USA; 2 Center for Applied AI, Sutter Health, Sacramento, CA 95833, USA; negin.moghadasi@sutterhealth.org; 3 Department of Radiology, University of California Davis, Sacramento, CA 95817, USA; aghasemiesfe@health.ucdavis.edu (A.G.);

**Keywords:** coronary computed tomography angiography, artificial intelligence, deep learning, atherosclerotic plaque, radiomics, photon-counting computed tomography, plaque quantification, coronary artery calcium, evidence map, scoping review

## Abstract

**Background/Objectives:** Coronary computed tomography angiography (CCTA) can quantify plaque, whose burden predicts myocardial infarction better than stenosis severity. We reviewed evidence for artificial intelligence (AI)-assisted plaque characterization. **Methods:** This targeted scoping review (PRISMA-ScR) used a non-exhaustive search, mainly of MEDLINE/PubMed (July 2026, supplemented in September). AI title selection (135 of 1415 titles) and author assessment of 141 records yielded 121 included records under the revised criteria. An AI re-screen of 1280 unselected records (blind second pass on 175) found 110 further eligible records after charting; two AI passes screened 3634 confirmatory records (κ 0.93). The corresponding author accepted AI recommendations after reviewing written assessments, not blind (for the 335 confirmatory records, in one instruction). Of the 543 eligible records, 236 were charted (121 + 110 + 4 + 1; full text for 121 of the 236, abstract alone for 115) and 307 mapped from abstracts only (not fully charted). **Results:** In symptomatic or referred patients, AI-derived plaque burden was associated with major adverse cardiovascular events (a property of the measurement, not evidence that AI improves outcomes). In one comparison, AI modestly improved discrimination over manual quantification (AUC 0.82 vs. 0.78) without significant net reclassification improvement. No randomized trial of AI-based plaque analysis reporting clinical endpoints was identified in the sources searched and assessed; TRANSFORM (NCT06112418) is recruiting. Three small same-patient comparisons found systematic between-platform differences; no adequately powered comparison of major commercial platforms was identified. Abstract-level mapping of the 307 additional records identified no randomized outcome, observed decision-impact or photon-counting prognostic study. **Conclusions:** AI-derived plaque measures carry prognostic information in symptomatic patients, but randomized evidence that AI-guided treatment reduces myocardial infarction or cardiovascular death in asymptomatic adults, or does so better than coronary artery calcium (CAC)-guided prevention, has not been established in the sources searched and assessed. Under the modeled effects, demonstrating benefit in the lowest-burden stage would need an impractically large trial; as a design proposal, we outline a stage-enriched randomized trial. AI-assisted plaque characterization is best understood as a throughput and within-system repeatability technology; cross-platform portability is not demonstrated, and it is not a validated treatment decision rule.

## 1. Introduction: From Stenosis-Focused CCTA to Plaque Phenotyping

CCTA is now a first-line investigation for stable chest pain on the strength of outcome data. In the 10-year SCOT-HEART analysis of patients with stable chest pain (*n* = 4146), CCTA-guided management reduced coronary heart disease death or non-fatal myocardial infarction (MI) versus standard care (6.6% vs. 8.2%; hazard ratio [HR] 0.79, 95% CI 0.63–0.99), with no increase in revascularization but sustained higher use of preventive therapy [1]. In that symptomatic population, the benefit appears to arise from better identification and treatment of atherosclerosis rather than more stenting, suggesting the scan’s main role is detecting atherosclerosis [2].

Stenosis severity is an imperfect surrogate for event risk. In the same SCOT-HEART population, low-attenuation plaque (LAP) burden was the strongest predictor of fatal or non-fatal MI (adjusted HR 1.60 per doubling), independent of risk score, calcium score and area stenosis; LAP burden > 4% conferred a nearly five-fold risk [3]. Qualitatively assessed adverse plaque features were also associated with events (HR 3.01), but not independently of the calcium score, so visual assessment appears to add little once burden is accounted for [4]. In patients with coronary artery disease undergoing invasive fractional flow reserve (FFR) measurement, high-risk plaque characteristics likewise carried prognostic weight mainly in the non-ischemic range (FFR > 0.80) [5].

The association has since been reproduced in larger datasets. A systematic review of 38 studies (15 eligible for pooling) found pooled HRs for major adverse cardiovascular events (MACEs) of 3.93 for total plaque volume and 2.81 for LAP volume [6]. A larger review (52 studies, *n* = 58,123) found that CT-derived FFR (HR 6.14) and high-risk plaque (HR 4.05) were most strongly associated with MACEs, with moderate-to-severe heterogeneity [7]. In a PROMISE substudy of symptomatic outpatients, total and non-calcified plaque burden independently predicted MACEs after adjustment for risk factors, statin use and qualitative findings, although the authors stated that clinical utility “requires prospective evaluation” [8]. Pericoronary adipose tissue (PCAT) attenuation and LAP burden are complementary, with LAP being the stronger (AUC 0.71 vs. 0.64) [9]. Not all of this evidence derives from AI-based quantification; it establishes the prognostic value of plaque measurement itself, and Section 3 examines the AI-specific evidence. The target lesion has also been redefined. A recent position statement argues that the classical thin-cap fibroatheroma model is too narrow, since superficial erosion causes up to 40% of acute coronary syndromes, and plaque burden, inflammation, healing, hemodynamics and subtended myocardium all contribute [10].

The populations studied determine how this evidence can be used. In the charted dataset, outcome studies of quantified plaque enrolled patients referred for CCTA for symptoms or suspected coronary disease, with acute chest pain or acute coronary syndromes, or with known coronary disease. Among the 235 records charted before the confirmatory search was screened, no outcome study of AI-derived CCTA plaque measurement in an asymptomatic screening population was identified (one community-cohort study, listed in Table S1 as R2-N005, analyzed radiomic features of non-contrast calcium-score images, not CCTA plaque); 8 of the 54 outcome studies did not state the clinical population (Table S1, clinical-population column). Screening the confirmatory search (Section 2.3) added one study close to that population, which was then charted. In 23,314 randomly selected adults aged 50–64 years from a population-based cohort without prior atherosclerotic cardiovascular disease, deep-learning-quantified total plaque volume was associated with coronary heart disease death or myocardial infarction over a median of 7.8 years and improved prediction beyond SCORE2 and the segment involvement score (C-statistic 0.798 versus 0.783); risk rose with plaque volume even among participants with a coronary artery calcium score of zero [11]. This is evidence of improved prediction in a general population, not of benefit from measurement-guided treatment. In asymptomatic adults, the established imaging test for prediction is coronary artery calcium (CAC) scoring. In the population-based Multi-Ethnic Study of Atherosclerosis (6722 adults without clinical cardiovascular disease), CAC predicted coronary events beyond standard risk factors in each of four racial and ethnic groups [12], and a CAC score of zero was the strongest of 13 negative risk markers [13]. In the CONFIRM registry, among 7590 individuals without chest pain or known coronary disease, adding CCTA to risk factors and CAC raised the C-statistic only from 0.75 to 0.77, with negligible net reclassification [14]. A CAC score of zero does not exclude plaque; however, in the Miami Heart Study, 49% of 2359 asymptomatic adults had coronary plaque on CCTA, including 16% of those with zero CAC [15]. These findings concern prediction and prevalence, not treatment benefit; prognostic associations from diagnostic chest pain cohorts cannot establish that screening asymptomatic adults is beneficial. In a lower-risk population, the balance between the benefit of earlier treatment and the harms of further testing and long-term therapy may differ and must be tested there. Throughout, we name the population for each prognostic finding and keep three kinds of findings apart: improved prediction, changes in prescribing or management, and improved clinical outcomes.

The main practical obstacle has been measurement. Expert manual quantification took a mean of 25.7 min per patient, against 5.7 s for a deep learning system, with excellent agreement (intraclass correlation coefficient [ICC] 0.964) [16]. Inter-observer data concur: among expert readers, per-vessel agreement on total plaque volume was moderate to high (ICC 0.78 for a single reader, 0.91 for mean scores) but fell to 0.68 for plaque composition. Reader–AI agreement was lower still, with weighted κ of 0.23–0.34 for the calcified/non-calcified split and 0.17–0.26 for high-risk plaque designation [17]. Standardized definitions [18] and consensus roadmaps [19] exist, but the need for throughput and reproducibility makes AI an enabling technology rather than a convenience [20].

The readouts reviewed here fall into three domains. Anatomical readouts quantify plaque burden and composition (Section 3 and Section 5). Inflammatory readouts measure pericoronary adipose tissue as a vascular-inflammation marker (Section 3.5). Acquisition-related factors, such as photon-counting detector CT, are not AI applications but change the images from which AI-derived measurements are made (Section 4). The three domains are reviewed together because each yields a quantitative readout from the same CCTA acquisition and faces the same question of cross-platform and cross-scanner portability. CT-derived fractional flow reserve (FFR-CT), a physiological readout, is out of scope; studies of it are cited only as context.

**Figure 1:**
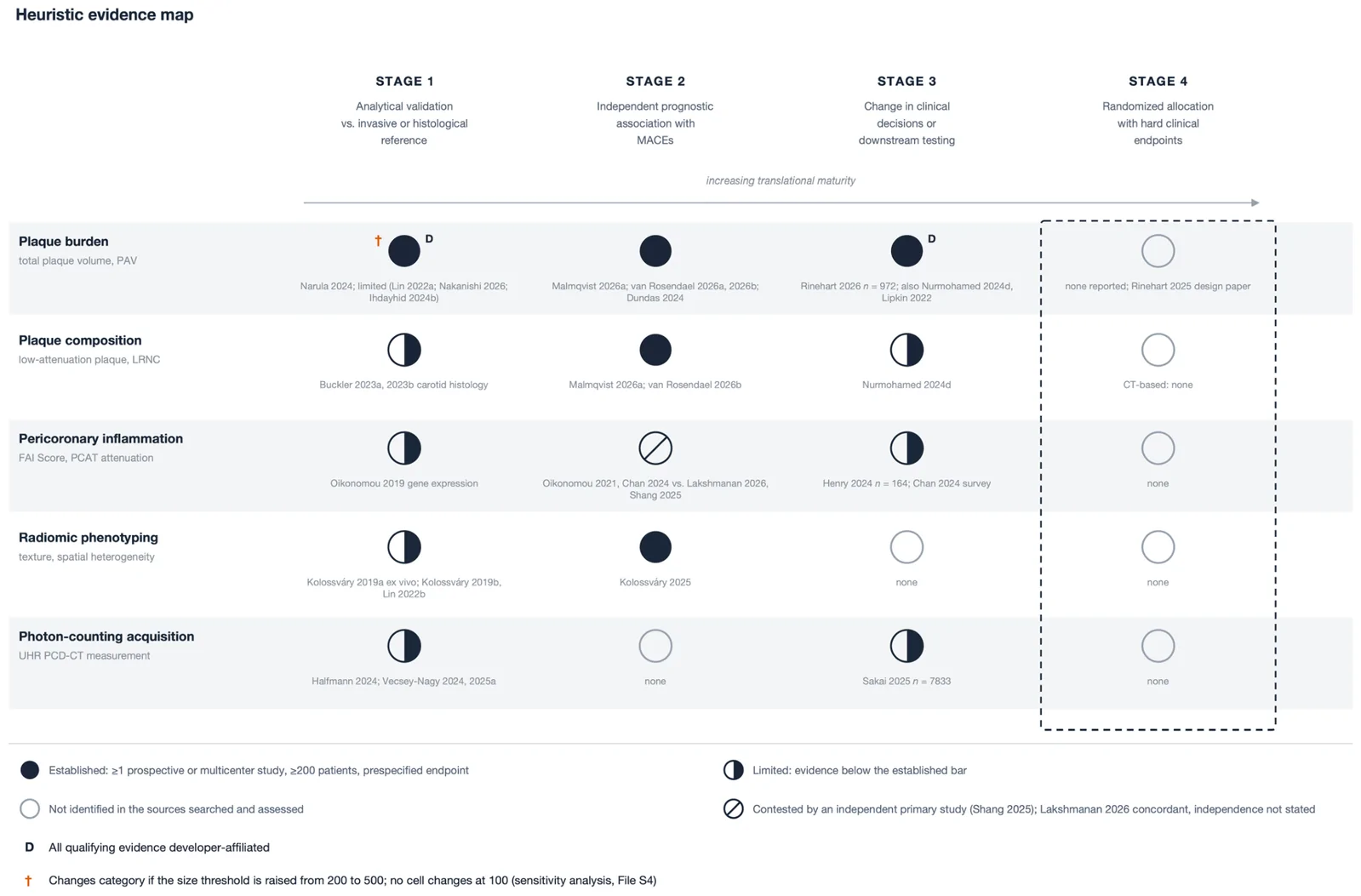
Heuristic evidence map of the translational status of AI-assisted coronary CT readouts, graded with a framework proposed by the authors. Rows are the five claim domains; columns are four sequential stages of translational evidence. Cells are scored on AI-derived or automated quantification only. Filled: at least one charted study at that stage that is prospective or multicenter, includes at least 200 patients (or 200 lesions at stage 1; a convention rather than a power-based threshold) and has a prespecified endpoint; at stage 1, the reference must be invasive or histological and able to measure the readout, at stage 3, the change must be observed rather than intended or modeled, and at stage 4, the primary endpoint must be death or myocardial infarction. Half-filled: evidence at that stage, none meeting that bar. Open: no charted study identified in the sources searched and assessed. Slashed: an independent primary study meeting the same design and size bar reaches a materially different conclusion. At stage 2 of inflammation, this is [24,25]: ref. [25] is independent (no competing interests reported), and ref. [24] reaches the same conclusion, but its independence is not stated (its disclosure statement could not be accessed). D: all qualifying evidence is developer-affiliated; that is, it comes only from studies whose authors are affiliated with, or funded by, the commercial developer of the platform; software developed by a study’s own academic authors is classed as academic in-house and is not marked D (File S4). Study labels appear beneath each symbol; File S4 justifies every cell’s score. At stage 2 of plaque burden, the qualifying studies of commercial platforms are developer-affiliated, but the cell carries no D because studies without commercial developer affiliation also meet the bar, including the general-population cohort [11] (File S4). Prognostic evidence from semi-automated, non-AI quantification [3,9] is not scored. A sensitivity analysis at 100 and 500 participants is given in File S4; cells that change are marked (†). The map shows the 236 charted records; File S6 compares it with the figure published at the first revision and with the 121 records charted at submission and lists the added records that contribute to a cell. The developer marker on plaque composition at stage 2 was removed at the second revision, when a general-population cohort from the screened confirmatory search [11] was charted from its full text and met the bar; adding the 307 records mapped from abstracts (File S7) changed no other cell or developer marker (File S6). The inflammation row is scored at class level; the contesting studies [24,25] measured pericoronary adipose tissue attenuation rather than the FAI Score; ref. [25] reported no competing interests, and the disclosure statement of [24] could not be accessed. The rightmost column is open for every domain: AI-DECIDE [26] is an observational cohort (ClinicalTrials.gov NCT06376851) whose published primary outcomes report change in management rather than clinical events [27], and TRANSFORM (ClinicalTrials.gov NCT06112418), the first randomized allocation to AI-based plaque analysis, is recruiting and has not reported. FAI, fat attenuation index; LAP, low-attenuation plaque; LRNC, lipid-rich necrotic core; MACE, major adverse cardiovascular event; PAV, percentage atheroma volume; PCAT, pericoronary adipose tissue; UHR PCD-CT, ultra-high-resolution photon-counting detector CT. Labels in the image give the first author and year of the cited reference (a, b, c or d where first author and year repeat): Malmqvist 2026a [11]; Lin 2022a [16]; Lakshmanan 2026 [24]; Shang 2025 [25]; Rinehart 2025 [26]; Rinehart 2026 [27]. The key for the remaining labels, whose references are first cited later in the text, is given in Supplementary File S4 (Table S4-K).

This targeted scoping review examines what the published literature, rather than vendor claims, currently supports. It is a targeted evidence map: a structured, non-exhaustive sample of the indexed literature, charted (or abstract-mapped) against a pre-specified framework. Its conclusions about gaps apply only to the sources searched and the records assessed. Several recent reviews cover adjacent ground [21,22,23]. We emphasize separating what has been validated from what has only been asserted and identifying the evidence’s limits. Framed as population, concept and context, the review asks three questions about adults undergoing coronary CT angiography: What indexed evidence supports AI-assisted characterization of coronary plaque at each stage, from analytical validation to randomized clinical outcome? How does that evidence extend to clinical, regulatory and economic settings? And where was it not identified in the sources searched and assessed? Section 2 sets out how the literature was identified. Figure 1 summarizes the central finding across five claim domains. Analytical validation against an invasive reference is established for plaque burden and limited elsewhere. Prognostic association is established for burden, composition and radiomic phenotyping and contested for inflammation. At the fourth stage, randomized allocation with hard clinical endpoints, no study was identified for any domain in the sources searched and assessed.

## 2. Materials and Methods

### 2.1. Design and Reporting

**Figure 2:**
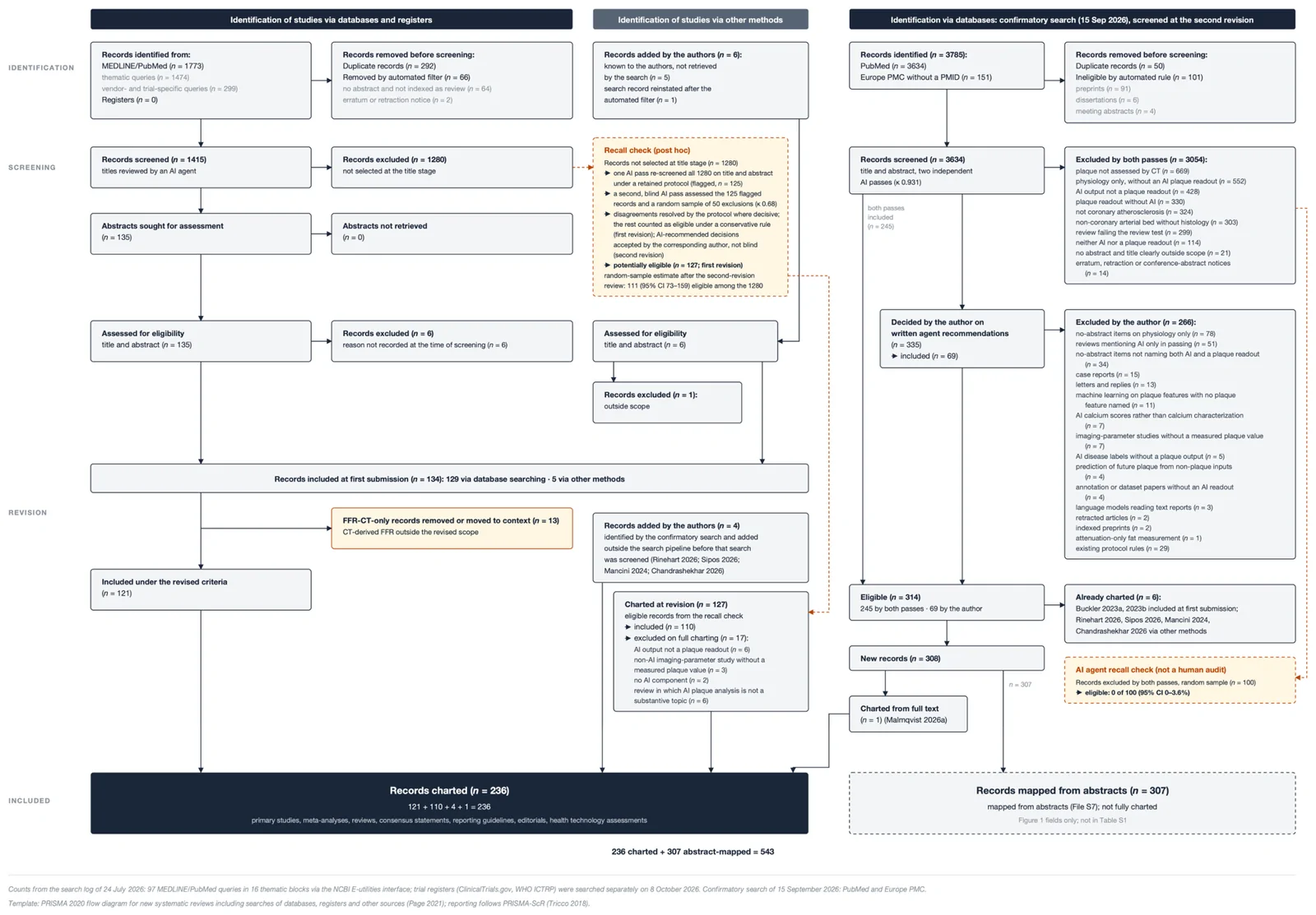
PRISMA 2020 flow diagram [29] of the identification, screening, eligibility assessment and inclusion of records, reported in accordance with PRISMA-ScR [28]. Counts are taken from the search log of 24 July 2026 (Section 2.2): the searches returned 1773 records (1474 from thematic queries and 299 from vendor- and trial-specific queries), of which 292 were duplicates. An automated filter removed a further 66 records: 64 without an abstract and not indexed as reviews and 2 erratum or retraction notices. Of the 1415 records whose titles were reviewed by an AI agent (Section 2.3), 135 were selected under selection rules set by the corresponding author, who then evaluated them on title and abstract, and 129 were included; reasons for the 6 exclusions at this stage were not recorded. The authors added 6 records outside the search pipeline—5 not retrieved by the search and 1 search record reinstated after the automated filter—of which 5 were included and 1 was excluded (outside scope), giving 134 under the original criteria; 13 records whose only readout was CT-derived FFR or another physiological measure were reclassified as context under the revised eligibility criteria (Section 2.3), leaving 121 included records. Trial registers (ClinicalTrials.gov and the WHO ICTRP) were searched separately on 8 October 2026 (Section 2.2); registry records cited in the text are context and are not among the included records. For the records charted at submission, open-access full texts (56 records) were used only to verify charted numeric values, not for selection; at revision, full texts were used for charting where accessible (Section 2.6). Box labels are adapted from “reports” to “abstracts” because eligibility was assessed on abstracts. References [28,29] are cited as reporting guidelines and are not among the 121 included records. One AI pass re-screened on title and abstract all 1280 records not selected at the title stage under a retained protocol and flagged 125; a second, blind AI pass assessed the 125 flagged records and a random sample of 50 exclusions (κ 0.68). Disagreements were resolved by the protocol where it was decisive; the remaining records were counted as eligible under a conservative rule at the first revision, and AI-recommended decisions on them were accepted by the corresponding author at the second revision, not blind to the recommendations. Potentially eligible *n* = 127 (first revision). Random-sample estimate after the second-revision review: 111 (95% CI 73–159) eligible records among the 1280. At revision, the 127 eligible records were charted: 110 were included, and 17 were excluded on full charting (AI output not a plaque readout, 6; non-AI imaging-parameter study without a measured plaque value, 3; no AI component, 2; review in which AI plaque analysis is not a substantive topic, 6). Records identified by the confirmatory search and added by the authors outside the search pipeline before that search was screened: *n* = 4 [27,30,31,32] (identification via other methods). Total charted: 121 + 110 + 4 + 1 = 236 (the last from the screened confirmatory search, below). Confirmatory search of 15 September 2026, screened at the second revision (Section 2.3; File S7): 3785 records not in the July search (PubMed 3634; Europe PMC without a PMID 151); removed before screening: duplicates 50, preprints 91, dissertations 6 and meeting abstracts 4. Screened on title and abstract by two independent AI passes: 3634 (κ 0.931). Excluded by both passes: 3054 (plaque not assessed by CT, 669; physiology only, without an AI plaque readout, 552; AI output not a plaque readout, 428; plaque readout without AI, 330; not coronary atherosclerosis, 324; non-coronary arterial bed without histology, 303; review failing the review test, 299; neither AI nor a plaque readout, 114; no abstract and title clearly outside scope, 21; erratum, retraction or conference-abstract notices, 14). Decided by the corresponding author on written agent recommendations: 335 (included 69; excluded 266: no-abstract items on physiology only, 78; reviews mentioning AI only in passing, 51; no-abstract items not naming both AI and a plaque readout, 34; case reports, 15; letters and replies, 13; machine learning on plaque features with no plaque feature named, 11; AI calcium scores rather than calcium characterization, 7; imaging-parameter studies without a measured plaque value, 7; AI disease labels without a plaque output, 5; prediction of future plaque from non-plaque inputs, 4; annotation or dataset papers without an AI readout, 4; language models reading text reports, 3; retracted articles, 2; indexed preprints, 2; attenuation-only fat measurement, 1; existing protocol rules, 29). Eligible: 314 (245 by both passes, 69 by the author), of which 6 were already charted ([27,30,31,32,33,34]); of the 308 new records, 1 was charted from its full text [11], and 307 were mapped from their abstracts on the Figure 1 fields (File S7). AI agent recall check (not a human audit) of 100 records excluded by both passes: 0 eligible (95% CI 0–3.6%). Reports and studies: grouping under a stated rule merged no records, so the 236 charted and 307 mapped records remained, totaling 543 records (18 possible companion sets listed in File S7). PRISMA, Preferred Reporting Items for Systematic Reviews and Meta-Analyses; PRISMA-ScR, PRISMA extension for Scoping Reviews. Labels in the image give the first author and year of the cited reference (a, b, c or d where first author and year repeat): Malmqvist 2026a [11]; Rinehart 2026 [27]; Tricco 2018 [28]; Page 2021 [29]; Sipos 2026 [30]; Mancini 2024 [31]; Chandrashekhar 2026 [32]; Buckler 2023a [33]; Buckler 2023b [34].

This targeted scoping review was conducted and reported in accordance with the Preferred Reporting Items for Systematic Reviews and Meta-Analyses extension for Scoping Reviews (PRISMA-ScR) [28]. The completed PRISMA-ScR checklist, with the location of every reported item, is provided as Supplementary File S2; Figure 2 shows the flow of records through identification, screening, eligibility assessment and inclusion, using the PRISMA 2020 flow diagram template [29]. The review protocol was neither registered in a public registry nor published separately; the methods are reported in full in Section 2.2, Section 2.3, Section 2.4, Section 2.5, Section 2.6 and Section 2.7 and in File S1. A scoping review was chosen because the objective was not to answer a single comparative question but to map the extent, range and nature of the evidence for a rapidly evolving technology across analytical, prognostic, regulatory and economic domains and to identify claims for which evidence was not identified in the sources searched and assessed. It is a targeted evidence map: its evidence base is a prioritized sample drawn from capped searches of a single database, not an exhaustive map of the literature, and the sample is charted or, for the 307 abstract-mapped records, mapped against a pre-specified framework (Figure 1), with gaps reported only for that sample. Statements that evidence was not identified refer to the sources searched and the records assessed (Section 2.2). Consistent with PRISMA-ScR, we did not conduct a formal risk-of-bias assessment of individual sources or a quantitative synthesis; where the literature permitted pooling, we cite published meta-analyses [6,7] rather than performing our own. Records were identified, screened and charted as set out in Section 2.2, Section 2.3, Section 2.4, Section 2.5, Section 2.6 and Section 2.7 and summarized in Figure 2.

### 2.2. Search Strategy

On 24 July 2026 we searched MEDLINE/PubMed programmatically through the NCBI E-utilities application programming interface, running 97 queries in 16 thematic blocks across two rounds. Publication-date limits were 2019–2026 in the first round (44 queries in nine blocks) and 2018–2026 in the second (53 queries in seven blocks), applied uniformly within each round. These original thematic searches were not re-run. An uncapped confirmatory search of MEDLINE/PubMed and Europe PMC on 15 September 2026 tested their coverage and was screened at the second revision; its contributions are reported separately below. Trial registries were searched on 8 October 2026. Records published after these dates were not systematically captured. No language restriction was applied at the search stage; every record meeting the eligibility criteria was published in English. File S1 provides the verbatim query strings, raw returned records and triage script.

Each query was a free-text PubMed search string; apart from author name ([au]) and exact-phrase searches, queries did not use controlled-vocabulary (MeSH) terms or field tags. Across the 97 queries, the terms covered three concepts: (i) imaging modality—coronary computed tomography angiography, CCTA, coronary CTA, photon-counting detector CT, spectral CT, dual-energy CT; (ii) analytical method—artificial intelligence, machine learning, deep learning, convolutional neural network, radiomics, automated quantification; and (iii) target—atherosclerotic plaque, plaque burden, low-attenuation plaque, pericoronary adipose tissue, fat attenuation index, high-risk plaque, vulnerable plaque, fractional flow reserve. Each query was sorted by relevance and returned at most its 40 highest-ranked records, so the search was designed to capture the most relevant records per query rather than every matching record.

Vendor-specific searches used the product and corporate names of each commercial platform discussed in Section 3, and author name searches covered several research groups; no affiliation-field searches were run. These searches underpin the negative finding in Section 3.7 (indexed validation not identified in the sources searched and assessed); File S1 reproduces the supporting queries. The authors added six further records outside the search pipeline, reported separately in Figure 2: five known to them that the search did not retrieve and one returned by the vendor-specific search that was reinstated after the automated filter had removed it for lacking an abstract.

After drafting, a confirmatory search was run on 15 September 2026 to test the reproducibility of the searches above. A single structured query, built from controlled-vocabulary and title/abstract terms in the same three concepts and run without a record cap, returned 4058 records from MEDLINE/PubMed and, as free text, 2784 from Europe PMC (at most 4159 unique records, of which 424 had already been identified in July; the first revision reported 4209, but 50 of the Europe PMC records without a PMID duplicate PubMed records by title). It retrieved 79 of the 134 originally included records (59.0%) and 79 of the 81 included records that fall inside all three concepts (97.5%). The included records it did not retrieve were background, regulatory, reporting-guideline and detector-physics records, outside the artificial intelligence concept by design. Its 3785 additional records (3634 from PubMed and 151 from Europe PMC without a PMID; at most 3735 unique) were screened at the second revision (Section 2.3; Figure 2; File S7). Four records identified by the confirmatory search and already cited in the text for specific statements [27,30,31,32] had been added by the authors outside the search pipeline before this screen and were charted at revision (Figure 2); one of them [27] is the only study that fills the plaque burden cell at stage 3 of Figure 1 (File S6). Both screening passes later included all four, as well as two records already included at submission [33,34]. One further eligible record, a general-population outcome study [11], was charted from its full text because it bears on Figure 1 and Section 1 and Section 9; the other 307 were mapped on the Figure 1 fields from their abstracts only, are reported separately (File S7) and are not among the 236 charted records. Statements that evidence was not identified should be read throughout as “not identified in the sources searched and the records assessed”, that is, in the July 2026 search, the re-screen and the screened confirmatory search; for the 307 records mapped from abstracts only, evidence reported only in a full text would not have been captured. Such a statement is not a claim that the evidence does not exist. File S1 gives the query string, counts and per-record outputs and File S7 the per-record screening decisions. ClinicalTrials.gov (API v2; four queries restricted to randomized interventional studies) and the WHO ICTRP (seven queries) were searched on 8 October 2026 for randomized trials in which AI-based or quantitative CCTA plaque analysis guides management; query strings, hit counts and the screened list are in File S1. Only TRANSFORM (ClinicalTrials.gov NCT06112418), which is recruiting, met all criteria, and no registered trial had reported clinical endpoint results (Section 5.1).

### 2.3. Eligibility Criteria and Selection

We included indexed primary studies, meta-analyses, narrative and systematic reviews, consensus statements, reporting guidelines, health technology assessments and editorials concerning AI-assisted characterization of coronary atherosclerotic plaque on CT, together with the inflammatory (pericoronary adipose tissue) readouts derived from the same acquisitions. Studies whose only CT-derived readout was fractional flow reserve or another physiological measure, without a plaque readout, were excluded; this criterion was made explicit at revision, and 13 previously included records of this kind are no longer counted as included (Figure 2). Studies of non-coronary arterial beds were included only where they supplied histological ground truth unobtainable in coronary specimens (as in [33,34,35,36]).

We excluded, by design, vendor white papers, regulatory clearance summaries used as evidence of performance, conference abstracts without full publication and any performance claim traceable only to promotional material. This exclusion, the review’s main methodological choice, is why some widely repeated accuracy figures do not appear here. Where multiple publications reported the same cohort, we cite the primary analytical or outcome publication and note companion analyses alongside it (as with [37,38,39]). Screening was staged. An automated filter removed records published before 2015 (none because the search date limits began in 2018), erratum and retraction notices and records without an abstract that were not indexed as reviews; it did not apply the eligibility criteria above. The remaining 1415 records were listed by query block, with titles truncated to 118 characters and without abstracts or scores (File S1, shortlist); an AI agent screened these titles under selection rules set by the corresponding author and selected 135 records for assessment (Section 2.7). No relevance score or pre-specified cut-off was used, and the prompt for this step was not retained. The corresponding author then evaluated the titles and abstracts of these 135 records and of the 6 records added by the authors (141 in total) against the eligibility criteria, without independent duplicate screening; 2 had no abstract and were assessed on the title and bibliographic record. Eligibility was decided on title and abstract; full texts were not used for selection. Reasons were not recorded for six of the seven records excluded at this stage. The lack of dual independent screening is a limitation (Section 8). Figure 2 summarizes the selection process.

Because the title selection step could not be validated retrospectively, its sensitivity was measured directly in three stages. First, the agent’s selection of 135 records was taken as given (Stage 1). Second, a separate AI agent (Claude Opus 5.5, Anthropic, San Francisco, CA, USA) re-screened all 1280 records not selected, on full title and abstract, under a written protocol fixed before screening. The protocol gives the criteria above verbatim, operational decision rules and fixed reason codes and is retained in full in File S1. The agent was instructed to flag any record that might be eligible, and it flagged 125 (Stage 2). Third, a second, independent AI screening pass checked Stage 2 (Stage 3): a separate agent session decided the 125 flagged records and a seeded random audit of 50 of the Stage 2 exclusions (seed 20261009), from title and abstract only and without access to the Stage 2 decisions. Its decisions were fixed (time-stamped and hashed) before the two passes were compared. The two passes agreed on 144 out of 175 records (82.3%; Cohen’s κ = 0.68). Disagreements were resolved against the protocol where it was decisive, and the rule and evidence were logged. In the first revision, records on which both AI passes agreed were accepted as decided, and the records whose eligibility the protocol did not settle (*n* = 37) were counted as eligible under a conservative rule, without record-level review, giving 127 eligible records. At the second revision, AI-recommended decisions with written rationale for these 37 records and for the 6 re-screen records cited in the text were accepted by the corresponding author (24 October 2026) after he had reviewed the written assessments; his review was not blind to the recommendations. After this review and full charting of all 127 records (Section 2.6), 110 of the 1280 records were eligible.

A seeded random sample of 300 of the 1280 records (seed 20260915), screened first under the same protocol, gives a design-based estimate: 26 of the 300 (8.7%; 95% CI 5.7–12.4%) were eligible, an estimated 111 eligible records (95% CI 73–159) among the 1280. In the audit of 50 exclusions, the second pass judged none as eligible and three as unsure, and none was eligible after the corresponding author accepted the AI recommendations on the three unsure records (0 of 50; 95% CI 0–7.1%). Because both passes used the same model under the same protocol, this design cannot detect errors common to both. The eligible records are listed in File S1. At this revision, all of them were charted (Section 2.6): 110 were added to the dataset, and 17 were excluded on full charting (Table S1); the effect of adding them is reported as a sensitivity analysis (Section 8; File S6).

The 3785 records added by the confirmatory search (Section 2.2) were screened at the second revision under a separate written protocol, fixed and hashed before screening (summarized in File S1; the full protocol is available from the corresponding author on request). Fifty Europe PMC duplicates of PubMed records were removed, and 101 were excluded by deterministic rule (91 preprints, 6 dissertations and 4 meeting abstracts). The remaining 3634 records were screened on title and abstract by two AI passes (Claude Opus 5.5, Anthropic, San Francisco, CA, USA), run as separate sessions under an identical written instruction; the 204 records without an abstract were decided on the title. The decision files were hashed before comparison. The passes agreed on 98.1% of records (Cohen’s κ 0.931, 95% CI 0.915–0.946, across include, exclude and unsure; 0.942, 0.927–0.957, with unsure counted as include). Both passes included 245 records and excluded 3054; for 2814 of the 3054 exclusions (92%), they cited the same protocol rule. By the rule applied (where the passes cited different rules, the earlier rule in the protocol’s order), the exclusions were as follows: plaque not assessed by CT, 669; physiology only, without an AI plaque readout, 552; AI output not a plaque readout, 428; plaque readout without AI, 330; not coronary atherosclerosis, 324; non-coronary arterial bed without histology, 303; review failing the review test, 299; neither AI nor a plaque readout, 114; no abstract and title clearly outside scope, 21; and erratum, retraction or conference-abstract notices, 14. The remaining 335 records (68 disagreements and 267 that both passes marked unsure) were decided by the corresponding author, who, in a single instruction (25 October 2026), accepted an AI agent’s written recommendation for each record, including 16 rule-level proposals for recurring questions; this was not a record-by-record review, and it was not blind to the recommendations. He included 69 and excluded 266 (237 under 15 of the 16 rules, the remaining rule having led only to an inclusion, and 29 under existing protocol rules; Figure 2). In total, 314 records were eligible: 6 were already charted (the 4 records added [27,30,31,32] and 2 records included at submission [33,34]), and 308 were new, of which 1 [11] was charted (Section 2.6) and 307 were mapped from their abstracts. File S7 lists every record identified with both passes’ decisions, the final decision, the rule applied and a one-line reason.

A random sample of 100 records excluded by both passes (seed 20261025) was re-read by an AI agent, which judged none as eligible (0 of 100; exact 95% CI 0–3.6%, that is, at most about 111 of the 3054); this is an AI check, not a human audit. For the 307 mapped records, two independent AI extraction sessions extracted the fields used to score Figure 1 from the abstract and the disclosure fields of the PubMed record, and their disagreements were resolved against the abstract. These records were not fully charted and are reported in File S7, not in Table S1.

Records were counted as reports. Grouping reports into studies under a stated rule (a shared registration number with the same question, an update, erratum or preprint link or an identical cohort, authors and endpoint) merged none of the 543 records, which are therefore counted as 543 records (sources of evidence, not necessarily independent primary studies); 18 possible companion sets, mostly different analyses of shared cohorts or a design paper with its results paper, were kept separate (File S7). Four deviations from the screening protocol are recorded in its amendment log and listed in File S1. The screening sessions shared one working directory, so blinding between the passes rests on each session’s own report that it did not open the other pass’s output. One screening session had also been given the protocol, which names the four records already added, so the finding that both passes included those four is a weaker internal check than a fully blind one. One record identifier in one output file was corrected by hand before the files were hashed. The output folders were named differently from those in the protocol, and the files were copied unchanged before hashing.

### 2.4. Vendor Attribution

Vendor identification in this literature is inconsistent. Where the primary publication names the software, we name it. Where it does not, we attempted attribution in this order: (i) explicit statement in the Methods; (ii) conflict-of-interest or funding disclosure; and (iii) author affiliation. Attributions resting on (ii) or (iii) are flagged at each point of use and in Table 1. Where none of the three permitted attribution, the study is reported as vendor-unattributed (Section 3.8).

### 2.5. Appraisal and Synthesis

Studies were not scored with a formal risk-of-bias instrument. Instead, we appraised each study against the specific claim it was cited to support, attending to the reference standard, sample size relative to the endpoint, prospective versus post hoc design, blinding, single- versus multicenter recruitment, independence of the investigators from the platform developer and whether the endpoint was a measurement, a physician intention or a clinical event. Where independent and developer-affiliated assessments reached different conclusions, both are reported, and the discrepancy is left unadjudicated (Section 3.5 and Section 7.2). Published appraisals are cited where available, including assessment with the Prediction model Risk Of Bias ASsessment Tool (PROBAST) [40], Radiomics Quality Score (RQS) and METhodological RadiomICs Score (METRICS) analyses [41,42] and a review of adherence to the Checklist for Artificial Intelligence in Medical Imaging (CLAIM) [43]. The four-stage translational axis and the evidence grading in Figure 1 are the authors’ own framework, not a validated instrument; Figure 1 is therefore a heuristic evidence map, and its threshold of at least 200 participants is a convention rather than a power-based criterion (File S4). Comparative judgements between platforms and studies rest on attributes recorded in Table S1 or, for the abstract-mapped comparison, File S7 (sheet E), in particular investigator independence from the developer, the reference standard and the study design (Section 2.6). A sensitivity analysis, specified before the added records were scored, compares the reproducibility summary (Section 4.3) and the platform summaries (Table 1) between the 121 records charted at submission and the merged dataset, and Figure 1 across five defined versions: the figure published at the first revision, the 121 records under the corrected charting of this revision, and the 235, 236 and 543 records (File S6; Section 8).

### 2.6. Data Charting

Charted fields were as follows: bibliographic identifiers; study design; population and sample size; the reference standard; the endpoint, classified as a measurement, a physician intention or a clinical event; the quantification platform and the level of the attribution hierarchy (Section 2.4) on which that attribution rests; investigator independence from the platform developer; the detector technology used for the analysis; and the stage of the four-stage translational axis to which the record contributes. Charting used AI-assisted extraction from abstracts and, where available, open-access full texts (Section 2.7). A record is charted when its attributes were extracted into Table S1, from the full text where accessible and otherwise from the abstract and the disclosure fields of the bibliographic record (in the initial duplicate extraction of the 131 candidate records, full text was used for 72 and for 74 after later full-text checks); a record is mapped when the targeted fields in File S7, principally those used for Figure 1, were read from its abstract and disclosure fields. Of the 236 charted records, the full text was used for 121 (alone or with the abstract) and the abstract and disclosure fields alone for 115 (Table S1, column Q). For the 121 records charted at submission, no independent duplicate extraction was performed. At the second revision, an AI verification step re-checked every charted value against the abstract, the disclosure fields of the bibliographic record and, where accessible, the full text; AI-recommended decisions with written rationale were accepted by the corresponding author (24 October 2026) after he had reviewed the written assessments, and for each of the 13 judgement items raised at this revision, he chose the recommended option; his review was not blind to the recommendations, and values in 25 of the 121 records were corrected (Table S1). Each charted value is linked to its source record through the listed bibliographic identifier. The charted dataset for all 236 charted records is provided as Table S1. At revision, two independent AI extraction passes, each blind to the other, charted the 127 records identified by the re-screen and the 4 records added from the confirmatory search from the abstract and, for 72 of these 131 records in the initial extraction (74 after later full-text checks), the open-access full text. Factual disagreements were reconciled against the source text. The eligibility questions (20 records) and four judgement items that the source and protocol did not settle were decided by the corresponding author, who accepted the agents’ recommendations (24 October 2026). For the 58 studies that support Figure 1 and the summary tables (Section 3.1, Section 4.2 and Section 4.3), the design, developer affiliation, reference standard and evidence stage were checked against the full text where accessible (45 studies) and otherwise against the abstract and the disclosure fields of the bibliographic record; the result for each is given in Table S1 or, for the one study from the screened confirmatory search that was mapped from its abstract rather than charted (PMID 38634943), in File S7 (sheet E). For 56 of the cited records, open-access full text was retrieved from Europe PMC and processed programmatically with a large language model tool (Section 2.7) to verify the numeric values reported in this review against the source; values for the remaining records were verified against the PubMed abstract. The record added from the confirmatory search [11] was charted from its full text by an AI agent, not field by field by an author. The charted dataset is aggregated by claim domain in Figure 1, by platform in Table 1, by threshold in Section 4.2 and by reproducibility in Section 4.3. Figure 1, the figures in Section 3, Section 4 and Section 5 and the tables rest on the 236 charted records or on context references; the 1 abstract-mapped record used is marked in the reproducibility table (row ‡, Section 4.3) and shown in the inter-vendor panel of the agreement figure (Section 5.2); absence statements cover the 307 mapped records only at the abstract level (Section 2.2).

Evidence was synthesized narratively along a four-stage translational axis—analytical validation, prognostic association, decision impact and randomized clinical outcome—used consistently in Figure 1 and Table 1 and structuring the argument of Section 9.

### 2.7. Use of Generative Artificial Intelligence

A customized AI agent was developed for this review to run the literature search, process retrieved records and support research on the topic. The agent combined several large language models: Claude Opus 5 and Claude Fable 5.1 (Anthropic, San Francisco, CA, USA), Qwen 2.5 (Alibaba Cloud, Hangzhou, China) and GLM 5.2 (Zhipu AI, Beijing, China). These models were used to write the search and analysis code, review the titles of the 1415 screened records to select those for assessment (Section 2.3), retrieve and process abstracts and open-access full texts, verify numeric values and citations against the source records (Section 2.6), draft text and check wording and spelling. Two further agent sessions (Claude Opus 5.5, Anthropic, San Francisco, CA, USA) re-screened the records not selected under a retained protocol (Section 2.3): the first screened all 1280, and the second, blind to the first, assessed the 125 records the first had flagged and a random sample of 50 of its exclusions. At revision, further agent sessions (Claude Opus 5.5, Anthropic, San Francisco, CA, USA) were used: two extracted the charted fields for the added records independently and blind to each other, a third reconciled factual disagreements against the source, and another checked the studies supporting Figure 1 and the summary tables against open-access full texts. The values extracted for the added records were checked against the source by that third session, not field by field by an author. For the confirmatory-search records, two further agent sessions screened the 3634 records independently, and an agent wrote a recommendation for each of the 335 records they did not settle; the corresponding author accepted these recommendations in a single instruction without record-by-record review. An agent re-read a random sample of 100 exclusions (an AI check, not a human audit), and two independent agent sessions mapped 307 of the added records from their abstracts, with disagreements resolved against the abstract by a further agent session (Section 2.3). At the second revision, agent sessions (Claude Opus 5.5, Anthropic, San Francisco, CA, USA) also performed a language edit of the whole text and shortened the Abstract and, at the third revision, made the wording changes on charted and abstract-mapped records in the text and the Supplementary Files, corrected File S7 and the supplementary notes and re-rendered Figure 2 and three figures in Section 5; an independent audit agent (OpenAI Codex) checked the revised files (audit only; it did not write text); agent sessions also edited the code that renders the figures. The figures were rendered from code, written with the agent sessions described above, as vector graphics; GPT-5 (OpenAI) was used only to generate an image that is not among the figures of this paper; the plotted values are taken from the cited sources and from the tables. Final eligibility decisions on the 141 records assessed were made by the corresponding author, who had set the selection rules for the AI title screen; in the re-screen, records the protocol did not settle were first counted as eligible under a conservative rule, and at the second revision, an AI agent prepared a written recommendation for each of them and for each cited record, which the corresponding author accepted after reviewing the written assessments, not blind to the recommendations (Section 2.3 and Section 2.6). The authors reviewed and edited all AI-drafted text and take full responsibility for the content of this review; Section 2.6 states the extent of author checking of charted values.

## 3. The Current AI Landscape: Commercial Platforms and Their Published Validation

### 3.1. The Shared Pipeline, Attribution and Overview of Evidence

**Figure 3:**
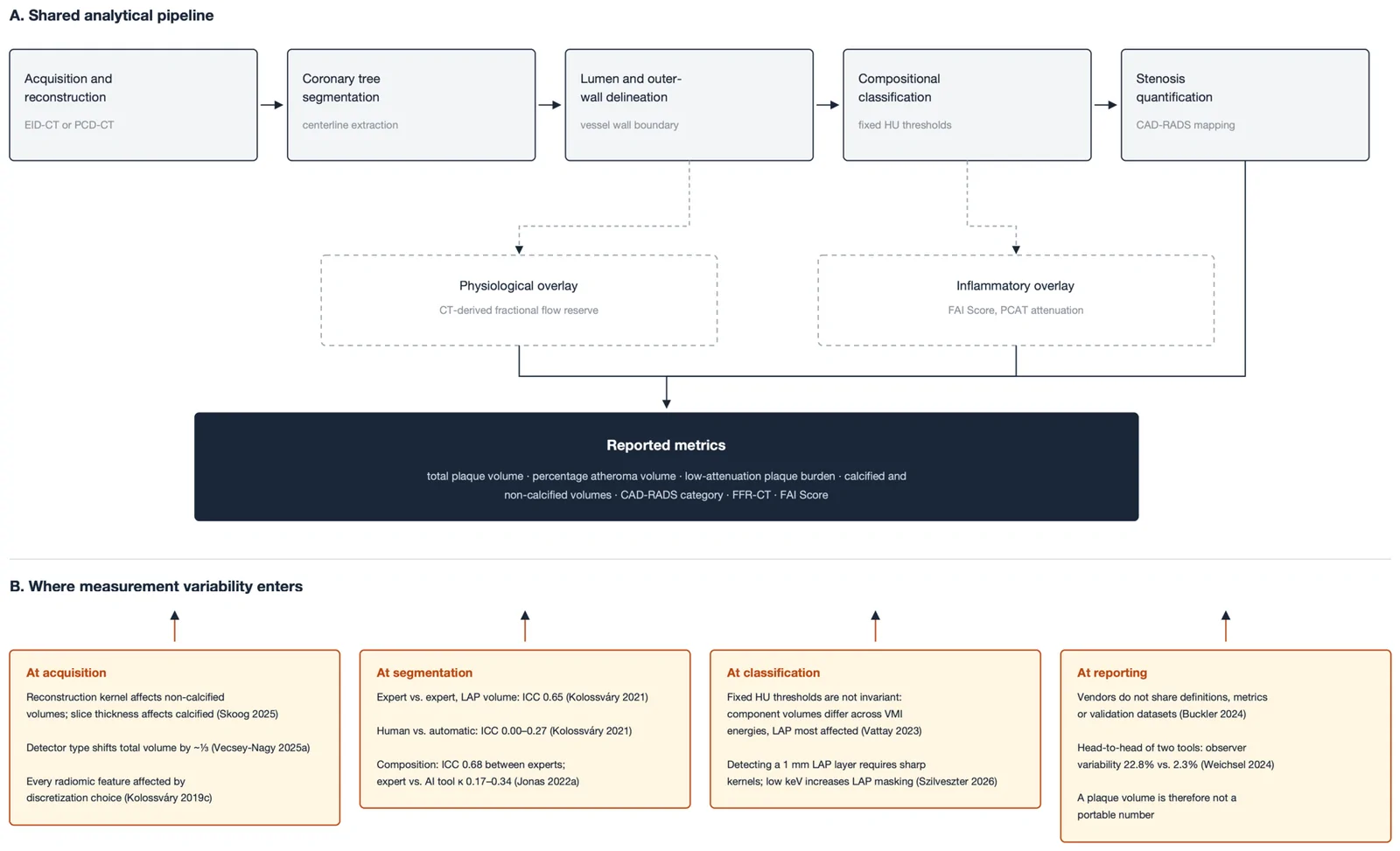
(**A**) The analytical pipeline shared by contemporary AI coronary plaque tools, with optional physiological and inflammatory overlays. (**B**) Where measurement variability enters, with the supporting evidence for each. The four points of entry are cumulative: a reported plaque volume carries the variance of every upstream stage. CAD-RADS, Coronary Artery Disease Reporting and Data System; EID-CT, energy-integrating detector CT; FAI, fat attenuation index; FFR-CT, CT-derived fractional flow reserve; HU, Hounsfield unit; ICC, intraclass correlation coefficient; LAP, low-attenuation plaque; PCAT, pericoronary adipose tissue; PCD-CT, photon-counting detector CT; VMI, virtual monoenergetic image. The FFR-CT overlay is context only (Section 2.3). Labels in the image give the first author and year of the cited reference (a, b, c or d where first author and year repeat): Jonas 2022a [17]. The key for the remaining labels, whose references are first cited later in the text, is given in Supplementary File S4 (Table S4-K).

Contemporary AI plaque tools share one pipeline: coronary segmentation, lumen and outer-wall delineation, attenuation-threshold compositional classification and stenosis quantification, increasingly with physiological or inflammatory overlays (Figure 3A). The platforms differ mainly in their validation reference standard and demonstrated endpoint. A shared pipeline means shared points of failure: Figure 3B maps where measurement variability enters, and each point recurs in Section 4 and Section 5. Unless marked, the evidence in Section 3, Section 4, Section 5, Section 6 and Section 7 comes from the 236 charted records or is cited as context; absence statements cover the 307 abstract-mapped records at the abstract level only (Section 2.2).

Some studies describe only “AI-QCT” or “an FDA-cleared cloud-based software”; platforms are attributed following the order in Section 2.4. Table 1 summarizes each platform’s reference standard, largest analytical validation, prognostic and decision-impact evidence and the principal limitation of that evidence base.

### 3.2. AI-QCT (Cleerly)

Cleerly’s atherosclerosis imaging–quantitative CT (AI-QCT) has the largest published evidence base (Table 1, File S5). Here, AI-QCT denotes this platform only; the generic approach is written as AI-assisted quantitative CCTA. AI-QCT has been validated against quantitative coronary angiography [51] and, in a developer-affiliated registry, against intravascular ultrasound (IVUS) [52]. Its plaque measures predicted MACEs incrementally to clinical models and the Coronary Artery Disease Reporting and Data System (CAD-RADS) 2.0 in patients referred for suspected coronary disease and in chronic coronary disease [37,38,39,53,54], although the gain over expert manual quantification was small [53] (Section 5.2). Decision-impact evidence rests on stated intent, post hoc analyses and modeling [55,56,69]; percentile work used an academic system, not this platform [70].

### 3.3. HeartFlow

HeartFlow’s most extensive evidence (Table 1, File S5) concerns FFR-CT [62,64,71,72,73], which is outside this review’s scope and cited as context. Its plaque tool (AI-QCPA) predicted events incrementally to stenosis and FFR-CT in the ADVANCE registry of patients with suspected coronary disease [62,63]. In REVEALPLAQUE, seven of whose authors are manufacturer employees, it correlated with IVUS at 0.91 for total plaque volume [60]; a smaller study also found strong agreement [61]. AI-DECIDE, a manufacturer-funded observational cohort (ClinicalTrials.gov NCT06376851) [26,74], has published pre–post outcomes on change in management [27] (Section 5.2).

### 3.4. Elucid

Elucid (Table 1, File S5) anchors tissue definitions to histology: in carotid endarterectomy specimens, its phenotype classification agreed with expert pathologists (weighted κ 0.82) [33,34], and treatment-related changes in the lipid-rich necrotic core (LRNC) were detected in small cohorts [57,58,59]. No study linking Elucid-derived plaque characterization to MACEs was identified in the sources searched and assessed; tissue-level accuracy does not establish prognostic performance. An Elucid-associated consensus Quantitative Imaging Biomarkers Alliance (QIBA) Profile states that vendors use differing definitions, metrics and validation datasets [75].

### 3.5. Caristo

Caristo’s CaRi-Heart (Table 1, File S5) uses the perivascular fat attenuation index (FAI) as a readout of coronary inflammation, an index originally validated against adipose tissue gene expression rather than a plaque measure [47]. In patients undergoing clinically indicated CCTA, the originating studies and ORFAN report large prognostic effects [48,49], including an ORFAN hazard ratio for cardiac mortality of 29.8 for an FAI Score in the top versus bottom quartile in all three coronary arteries [49], and a developer-affiliated cost-effectiveness model has been published [76]. Evidence from groups without developer affiliation is weaker. A National Institute for Health and Care Research (NIHR) Early Value Assessment found only one includable study, at a high risk of bias [40]. In RAPID-CTCA, among patients with suspected acute coronary syndrome, pericoronary adipose tissue attenuation added no discrimination beyond LAP burden [24] (its disclosure statement could not be accessed), a finding echoed by a recent review [77]. A further cohort identified by the recall check (Section 2.3), whose authors include an employee and a founder of the developer, reported independent prognostic value for CaRi-Heart risk [78]. We do not adjudicate the discrepancy.

### 3.6. Artrya

Artrya-affiliated work (Table 1, File S5) reports a reasonably powered stenosis evaluation but a high-risk-plaque test set of only 45 patients [44], with calcium-scoring [45] and perioperative-risk [46] models validated separately. Attribution to Artrya rests on author affiliation.

### 3.7. Circle Cardiovascular Imaging

Table 1 and File S5 detail this vendor. Searching by product and company name (File S1) did not identify a peer-reviewed study validating its coronary plaque quantification against an intravascular, histological or expert reference standard; published studies used the software as a measurement tool only. We make no claim about its plaque-analysis performance; that indexed validation was not identified in the sources searched and assessed is a finding, not an omission.

### 3.8. Independent and Unattributed Validation and the Comparability Problem

No adequately powered comparison of the major commercial platforms on the same examinations in the same patients, that is, one designed and sized to test their agreement, was identified in the sources searched and assessed. The comparisons identified are few and small: two successive generations of one manufacturer’s software in 50 patients [68], two platforms across 24 coronary segments [31] and two vendors’ tools, one a non-commercial prototype, in 170 patients [30]; the screen of the confirmatory search added a comparison of one deep learning platform with two general-purpose workstation packages in 19 patients [79]; and an earlier study outside the search period compared three commercial platforms in 47 patients [80]. None was designed or powered to establish the interchangeability of the major platforms. How far plaque burden, composition, percentage atheroma volume, low-attenuation plaque and high-risk-plaque classification vary with the software platform alone is unknown, one of this review’s principal gaps.

Academic groups have validated in-house or unattributed pipelines against several reference standards. One pipeline was validated against optical coherence tomography (OCT) (*n* = 91 patients, 153 co-registered lesions; r = 0.84, 1.8 min/patient) [65]; that study is not independent of the developer: the software is the authors’ in-house prototype with models developed on their core-laboratory data, and its segmentation method has since been integrated into commercial software from a company the corresponding author cofounded. In a 17-hospital Chinese development with four external test sets including a photon-counting dataset, a fully automated model matched IVUS and expert readers (all ICCs > 0.90) yet showed wide Bland–Altman limits of agreement and only moderate prognostic discrimination (C-index 0.64–0.74) [66]. A stepwise multicenter study in heavily calcified vessels, the most difficult setting, found deep learning assistance improved radiologists’ specificity by 7–11% and inter-reader agreement [67].

Comparability is limited even within one manufacturer’s product line: two successive software generations, compared head to head in 50 patients, showed inter- and intra-observer variability of 22.8% and 19.7% versus 2.3% and 0.2%, with a two-fold difference in processing time [68]. Cross-vendor comparisons are few and small and point the same way. Across 24 coronary segments, measures within one platform correlated highly whether adaptive or fixed Hounsfield-unit thresholds were used (R ≥ 0.819), but agreement between two platforms was poorer, particularly for low-attenuation plaque, where one platform returned very small volumes within a very narrow range. The authors conclude that the proprietary algorithm affects the measures more than the thresholding option does [31]. An earlier study outside the search period also found systematic differences in noncalcified plaque volume between three commercial platforms on the same examinations of 47 patients (Pearson r 0.55–0.68) [80]. In 170 patients and 550 lesions, two vendors’ tools differed systematically in median total plaque volume (67.0 versus 50.8 mm^3^, *p* < 0.001), with a mean between-software bias of 45.3 mm^3^ on energy-integrating detector CT and 15.0 mm^3^ on photon-counting CT [30]. None of these studies was designed to test interchangeability, and none establishes that a threshold derived on one platform can be applied on another; one of the two tools in the largest study [30] is a prototype not commercially available. With the QIBA observation that vendors do not share definitions, metrics or validation datasets [75], these comparisons suggest that plaque volumes cannot yet be assumed portable between platforms, nor can thresholds be assumed to transfer between them. These three small same-patient comparisons [30,31,80] documented systematic between-platform differences, largest for low-attenuation plaque where it was assessed, so interchangeability cannot be assumed. Because no adequately powered comparison of the major commercial platforms was identified in the sources searched and assessed, the size of disagreement between those platforms, as well as its clinical consequences, has not been established. The 110 records added from the re-screen contained no further between-platform comparison. The screen of the confirmatory search added one comparison of plaque measurement, mapped from its abstract and confirmed in its open-access full text [79]: in 26 coronary segments of 19 patients, fully automated segmentation by a deep learning platform agreed more closely with IVUS plaque volume (Pearson r 0.85; ICC 0.71) than did two scanner manufacturers’ general-purpose post-processing packages (syngo.via, Siemens: r 0.55, ICC 0.55; IntelliSpace Portal, Philips: r 0.54, ICC 0.41). The developer of the deep learning platform funded the study and employs one author, and the comparators are not the dedicated plaque-analysis platforms in Table 1. It adds to the evidence of between-platform differences without changing the conclusion that the major commercial platforms have not been compared in an adequately powered design.

## 4. Acquisition-Related Domain Shift: Photon-Counting and Spectral CT, and Radiomic Reproducibility

Photon-counting detector CT is not an AI technology. It is considered here as an acquisition-related source of domain shift: its detector and reconstruction alter the measurements AI tools produce, testing whether AI-derived plaque measurements and thresholds transfer across CT technologies.

### 4.1. What Photon-Counting Detector CT Has Established

PCD-CT improves spatial resolution, reduces electronic noise and provides intrinsic spectral information; first-in-human coronary work showed superior image quality versus energy-integrating detector (EID) CT, with phantom detectability 2.3-fold higher for lumen and 2.9-fold for non-calcified plaque [81]. The effect is most marked for calcified lesions. Within photon-counting acquisition, ultra-high-resolution (UHR) reconstruction gave more accurate in vitro stenosis measurement than standard resolution (mean bias 2.3% vs. 10.1%) and reclassified 54.4% of patients to a lower CAD-RADS category, a reconstruction effect rather than a detector effect [82]. Intra-individual comparisons showed lower percent diameter stenosis for calcified and partially calcified plaques, with 49.0% of 49 patients reclassified downward; agreement with quantitative coronary angiography, assessed in a subset of 12 patients and 56 lesions, was better for the UHR measurements [83]. On semiquantitative analysis, PCD-CT detected less severe but more extensive disease (52% reclassified downward, higher segment involvement) [84]. Clinically, in a retrospective single-center comparison of 7833 patients, those scanned on PCD-CT were referred less often for invasive angiography (9.9% vs. 13.1%); among those referred, revascularization was more frequent (43.4% vs. 35.5%), and vessel-level specificity across 1686 vessels was higher (98.0% vs. 93.0%) [85]. That comparison was not randomized, and detector type is confounded with scanner vendor.

UHR PCD-CT reduced segmented total plaque volume by nearly one-third versus EID-CT and improved LAP reproducibility from poor to excellent (inter-reader ICC 0.47 to 0.92) [86]. Reconstruction kernel and section thickness [87] and temporal [88] and spatial resolution [89] each shift the results systematically. Not every reconstruction change is consequential: a super-resolution deep learning reconstruction at 60% lower dose agreed with hybrid iterative reconstruction on plaque volumes and composition (ICC > 0.8) [90]. This record came from the recall check (Section 2.3); its agreement was reported as correlation rather than bias.

The established claim is technical: PCD-CT measures calcified and low-attenuation plaque more accurately and reproducibly than EID-CT. Whether this improves patient outcomes is unproven; no study testing it was identified in the sources searched and assessed.

### 4.2. The Threshold Problem This Creates

Because compositional classification uses fixed Hounsfield-unit thresholds, spectral reconstruction choices change the measured volumes. In 51 plaques from 51 patients, component volumes differed significantly across virtual monoenergetic image (VMI) energies, most for fixed-threshold LAP volume, and the authors called for normalization protocols [91]. Reconstruction kernel predominantly affects non-calcified quantification while slice thickness affects calcified volumes [92]. In phantoms, detecting a 1 mm LAP layer adjacent to iodine and calcium requires sharp kernels and higher iterative strength, and lower-keV images increase LAP masking [93]. In a single-institution study using a manufacturer’s prototype analysis software, with two manufacturer employees among the authors, harmonized settings achieved high inter-scanner correlation (r = 0.85–0.95 for total plaque in 38 patients and 77 vessels), which the authors translated into sample-size estimates for mixed-platform longitudinal trials [94]. Yet in the same study mean bias for total plaque volume ranged from 8.2 to 164.4 mm^3^, the pattern of correlation without agreement noted above, to which the same caution applies.

**Figure 4:**
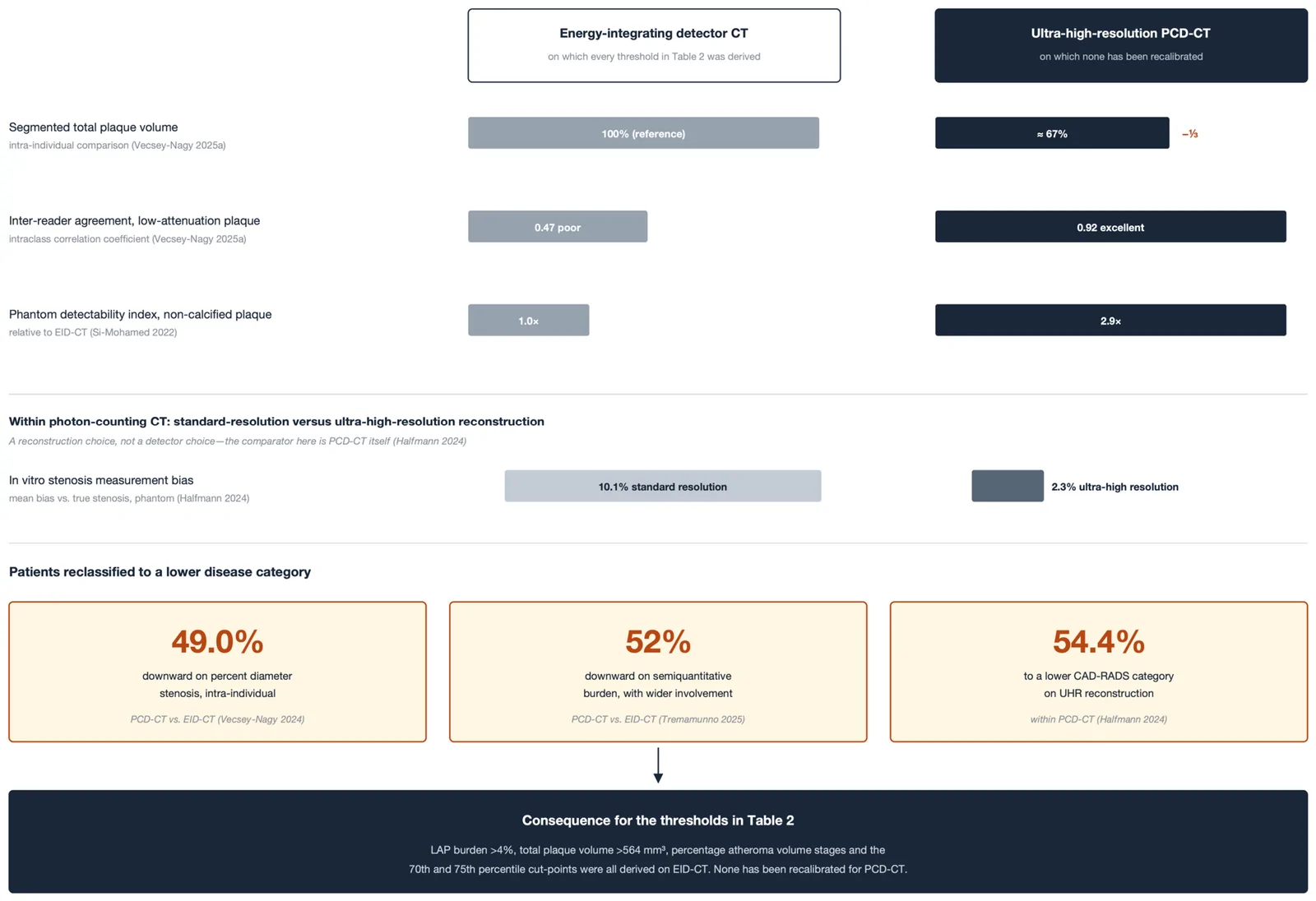
Detector dependence of coronary plaque measurement. Upper panel: intra-individual and phantom comparisons between energy-integrating detector CT and ultra-high-resolution photon-counting detector CT. Middle panel, shown separately because the comparator differs: standard-resolution versus ultra-high-resolution reconstruction within photon-counting acquisition, a reconstruction rather than a detector choice [82]. Lower panel: the proportion of patients reclassified to a lower disease category, with the comparator stated for each series: refs. [83,84] compare photon-counting with energy-integrating detector CT in the same patients, whereas ref. [82] compares two reconstructions of the same photon-counting acquisition. The consequence is stated in the lowest panel and detailed in Table 2. CAD-RADS, Coronary Artery Disease Reporting and Data System; EID-CT, energy-integrating detector CT; LAP, low-attenuation plaque; PCD-CT, photon-counting detector CT; UHR, ultra-high-resolution. Labels in the image give the first author and year of the cited reference (a, b, c or d where first author and year repeat): Si-Mohamed 2022 [81]; Halfmann 2024 [82]; Vecsey-Nagy 2024 [83]; Tremamunno 2025 [84]; Vecsey-Nagy 2025a [86].

This is the main practical consequence (Table 2): almost every prognostic threshold in Section 1 and Section 3—LAP burden > 4%, total plaque volume > 564 mm^3^, percentage atheroma volume stages and the 70th and 75th percentile cut-points—was derived on EID-CT. Transferability of these thresholds to PCD-CT has not been established in the sources searched and assessed; given the volume shift in Figure 4, it is unlikely to be one-to-one.

### 4.3. Radiomics: Prognostic Signal, Unresolved Reproducibility

Radiomics extends beyond volume and mean attenuation to texture and spatial heterogeneity. In an ex vivo study with histological ground truth, radiomics-based machine learning outperformed the visual assessment, low-attenuation area and mean Hounsfield unit for identifying advanced atheromatous lesions (AUC 0.73 vs. 0.65, 0.55 and 0.53) [97]. Against invasive and radionuclide references, radiomic parameters outperformed conventional features for attenuated plaque by IVUS, thin-cap fibroatheroma by OCT (AUC 0.80 vs. 0.66) and fluorine-18 sodium fluoride (^18^F-NaF) positivity (0.87 vs. 0.65), though in only 44 plaques from 25 patients [98], and distinguished culprit from non-culprit and stable lesions incrementally to high-risk features and volumes (0.86 vs. 0.76) [99]. In the largest study (1750 SCOT-HEART patients with stable chest pain, median follow-up 8.6 years), adding eigen-radiomic features to a model containing the risk score, Agatston score and obstructive CAD improved the C-statistic from 0.70 to 0.74, with the advantage emerging after the fifth year [100].

Spectral radiomics remains investigational: ex vivo spectral PCD-CT identified and histologically confirmed lipid-rich cores, spotty calcification and ulceration in a single carotid specimen [35] and quantified the fibrous cap thickness, cap area and LRNC area against histopathology in 20 excised plaques [36], while a radiomics/support vector machine model on multi-energy PCD-CT separated low- from high-risk plaques in 19 patients [101]. These are proofs of concept rather than clinical results, as a recent narrative review also notes despite its optimistic reading of the field [102].

Reproducibility warrants the most caution (Table 3). In a texture phantom scanned on one PCD-CT and four dual-energy systems, test–retest repeatability was excellent (97.1% of features), and intra-system reproducibility good, but inter-system reproducibility was poor: mean ICC 0.157, with no feature reaching ICC > 0.90 across scanners [103]. Within PCD-CT, features were robust to tube voltage, dose, iterative strength and kernel but sensitive to high-pitch scanning and slice thickness [104] and stable only under specific optimized reconstructions [105]. In CCTA, volumetric and radiomic metrics are largely robust to reconstruction algorithm (ICC > 0.90), yet discretization choices affect every radiomic variable [106]. Segmentation has a large effect: expert agreement was only fair for low-attenuation non-calcified plaque volume, and every human reader agreed poorly with automatic segmentation (ICC 0.00–0.27) except for calcified plaque volume, where agreement was good (0.73–0.88) [107]. Methodological quality is a further concern: across 15 cardiac CT radiomics studies, the mean Radiomics Quality Score was 9.9/36, with 2 out of 15 (13.3%) externally validated and none conducting phantom or cost-effectiveness analyses [41]. A 2026 METRICS/RQS assessment of 154 cardiac CT and magnetic resonance imaging (MRI) studies (93 of them CT) again found external validation, prospective design and open data uncommon, although overall quality was rated good by METRICS and lower by RQS [42].

## 5. Clinical Integration and Decision-Support Challenges

### 5.1. Where These Tools Plausibly Fit Today

**Figure 5:**
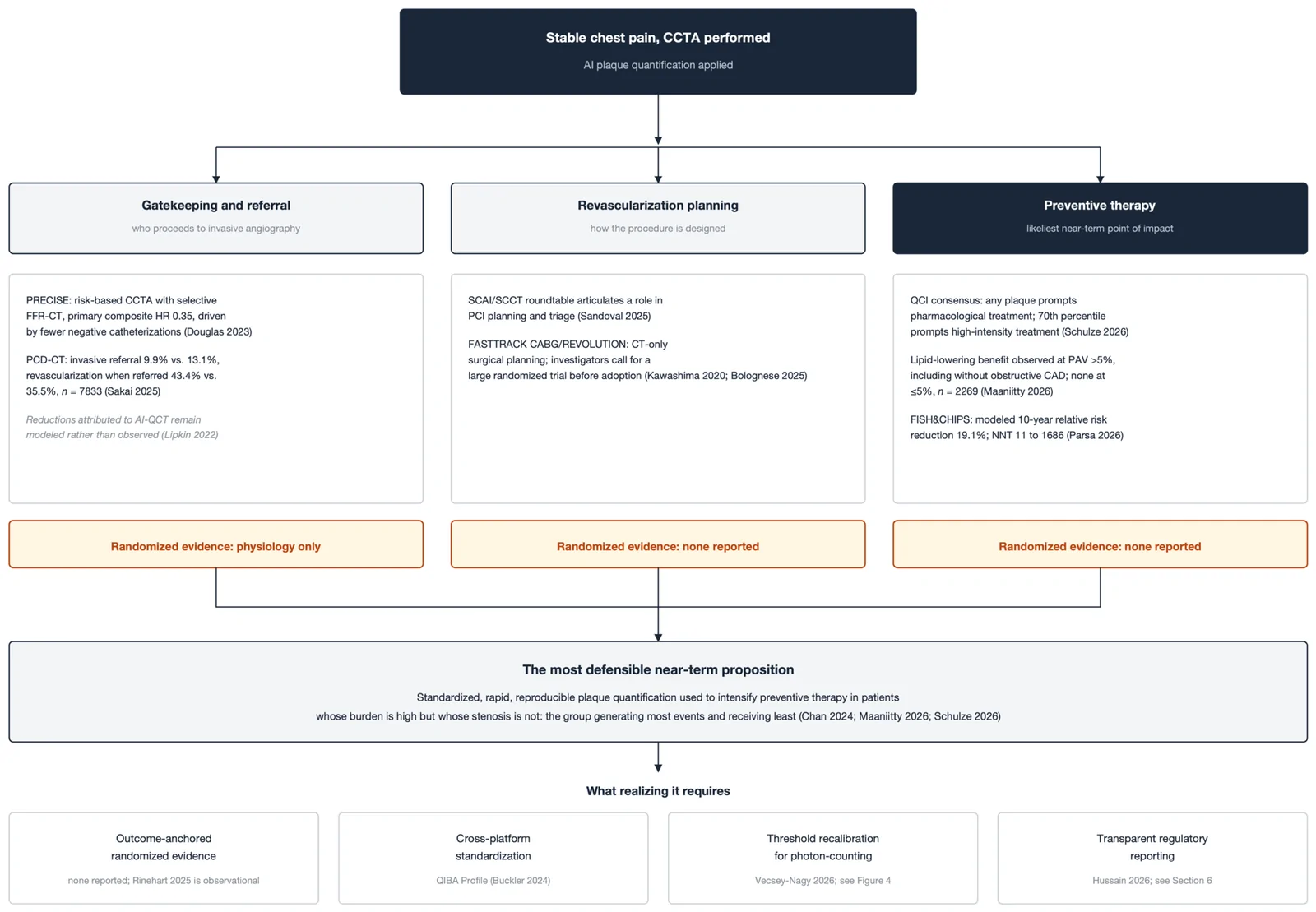
Proposed points of clinical integration for AI-assisted coronary plaque analysis, with the supporting evidence for each and the randomized evidence status beneath. The three branches converge on the proposition defended in Section 9, and the lowest row states its four prerequisites. CAD, coronary artery disease; CCTA, coronary computed tomography angiography; FFR-CT, CT-derived fractional flow reserve; NNT, number needed to treat; PAV, percentage atheroma volume; PCI, percutaneous coronary intervention. FFR-CT elements are context only (Section 2.3). Labels in the image give the first author and year of the cited reference (a, b, c or d where first author and year repeat): Rinehart 2025 [26]; Chan 2024 [49]; Lipkin 2022 [56]; Douglas 2023 [64]; Buckler 2024 [75]; Sakai 2025 [85]; Vecsey-Nagy 2026 [94]; Maaniitty 2026 [95]; Schulze 2026 [96]; Sandoval 2025 [115]; Kawashima 2020 [116]; Bolognese 2025 [117]; Parsa 2026 [118]. The key for the remaining labels, whose references are first cited later in the text, is given in Supplementary File S4 (Table S4-K).

Figure 5 maps the three proposed points of action for AI plaque analysis in the care pathway and the strength of evidence at each.

**Gatekeeping and referral.** The strongest integration evidence concerns physiology, outside the scope of this review and cited as context: PRECISE showed a risk-based CCTA/FFR-CT strategy improved clinical efficiency [64], and PCD-CT reduced invasive referral while raising the yield of those referred [85]. Reductions in invasive angiography attributed to AI-QCT remain modeled [56].

**Revascularization planning.** A roundtable of the Society for Cardiovascular Angiography and Interventions (SCAI) and the Society of Cardiovascular Computed Tomography (SCCT) articulated a role for CCTA in percutaneous coronary intervention (PCI) planning, triage and case-complexity assessment and identified unmet needs and barriers [115]. CCTA/FFR-CT-only surgical planning, tested in FASTTRACK CABG [116] within the REVOLUTION program, rests on physiology rather than plaque analysis and is cited as context; the program’s authors call for a large randomized trial before adoption [117].

**Preventive therapy** is the most likely near-term point of application, although no clinical benefit of AI-guided prevention was demonstrated in the sources searched and assessed. A Quantitative Cardiovascular Imaging (QCI) Study Group consensus statement, derived from a three-step Delphi process using age- and sex-adjusted percentile curves from DISCHARGE and SCOT-HEART, recommends pharmacological treatment for any plaque and high-intensity treatment at the 70th percentile of total plaque volume [96]. Consensus was reached on the treatment percentiles but not on treating any atherosclerotic plaque. A 2025 American College of Cardiology Scientific Statement, published during preparation of this review, gives guidance on clinical indications (including serial imaging), interpretation, reporting and standardization and names incorporation of the measurement into outcomes trials as a research direction [32]; it is a consensus statement rather than a guideline and does not settle the threshold question below. In 2269 symptomatic patients, lipid-lowering medication was associated with improved outcomes at percentage atheroma volume > 5%, including without obstructive CAD, but not at ≤5% [95]. FISH&CHIPS modeling in symptomatic patients (*n* = 6054) estimated a 10-year relative risk reduction of 19.1%, with the number needed to treat from 11 (highest stage) to 1686 (lowest) [118]. These are association and simulation data; no trial randomizing treatment intensity by AI-derived plaque burden was identified in the sources searched and assessed. Of the 14 registered randomized trials in which a CCTA plaque or inflammation readout guides management, only TRANSFORM (ClinicalTrials.gov NCT06112418), now recruiting, uses AI-based plaque analysis with a clinical-event primary endpoint; none of the 14 has reported clinical endpoint results (registry search of 8 October 2026; File S1).

**Coronary artery calcium as the comparator.** For primary prevention, the practical comparator for AI plaque quantification is not stenosis grading but coronary artery calcium (CAC) scoring, which current guidelines already use. The 2026 ACC/AHA dyslipidemia guideline, replacing the 2018 cholesterol guideline [119], recommends that “In adults at intermediate risk and select adults at borderline risk with no prior ASCVD, if the decision regarding LLT remains uncertain, a CAC score should be used for further risk stratification and to guide the decision to withhold, postpone, or initiate therapy” (Section 4.2.3.6 of guideline [120]; class of recommendation 1, level of evidence B-R) and that, if the CAC score is 0, the patient prefers to avoid drug therapy and no higher-risk conditions are present, deferring lipid-lowering therapy and repeating CAC testing in 3 to 7 years is reasonable (class 2a, level B-NR) [120]; ASCVD denotes atherosclerotic cardiovascular disease and LLT lipid-lowering therapy. For CCTA, the same section makes a weaker recommendation: in adults likely to have a high burden of noncalcified plaque, “selective use of coronary computed tomography angiography (CCTA) may be useful to inform risk assessment and guide decisions regarding treatment intensity of LLT” (class 2b, level B-NR) [120]. The 2021 ESC prevention guideline states that CAC scoring can reclassify cardiovascular risk upwards or downwards beyond conventional risk factors but gives no direct information on total plaque burden and can be low or zero when plaque is non-calcified [121]. CAC needs only a non-contrast acquisition and is already linked to treatment decisions. Its prognostic evidence in asymptomatic adults comes from large population cohorts and registries: CAC predicts coronary events across racial and ethnic groups [12], a score of zero was the strongest of 13 negative risk markers [13], and in individuals without chest pain, CCTA added little prognostic information once CAC was known [14], although 16% of asymptomatic adults with zero CAC had plaque on CCTA [15]. Randomized evidence for CAC-based strategies is itself limited. In DANCAVAS, inviting men aged 65–74 years to a screening program that included CAC scoring did not significantly reduce all-cause death over a median of 5.6 years (hazard ratio 0.95, 95% CI 0.90–1.00) [122]. In CAUGHT-CAD, CAC-informed prevention in asymptomatic adults with a family history of premature coronary disease slowed plaque progression over 3 years compared with usual care (total plaque volume +15.4 versus +24.9 mm^3^; *p* = 0.009), a surrogate endpoint [123]. In ROBINSCA, a population-based randomized screening trial in 28,928 adults at expected elevated risk, CAC scoring classified fewer participants as eligible for preventive treatment than the SCORE risk model (relative reduction 37.2% in women and 28.8% in men) [124]. This is a change in treatment indication; the trial’s clinical outcome results were not identified in PubMed (search of 26 October 2026). No trial comparing AI-CCTA-guided with CAC-guided prevention was identified in the literature and trial-registry searches (Section 2.2). SCOT-HEART 2 (ClinicalTrials.gov NCT03920176) randomizes asymptomatic adults aged 40–70 years with at least one risk factor to CCTA-guided or risk-score-guided prevention, with coronary heart disease death or non-fatal myocardial infarction as the primary outcome; it had not reported at our registry check (21 October 2026) and does not test AI plaque quantification. Whether AI plaque quantification improves primary prevention therefore needs testing against CAC-guided care, not just against clinical risk scores.

**Focal plaque treatment.** PREVENT randomized 1606 patients with non-flow-limiting vulnerable plaques to preventive PCI plus medical therapy or medical therapy alone; preventive PCI reduced the 2-year primary composite (0.4% vs. 3.4%) [125]. This supports the concept that plaque-directed treatment can alter outcomes, but plaques were identified by intracoronary imaging, not CT, so the trial does not support CT-based selection.

**Figure 6:**
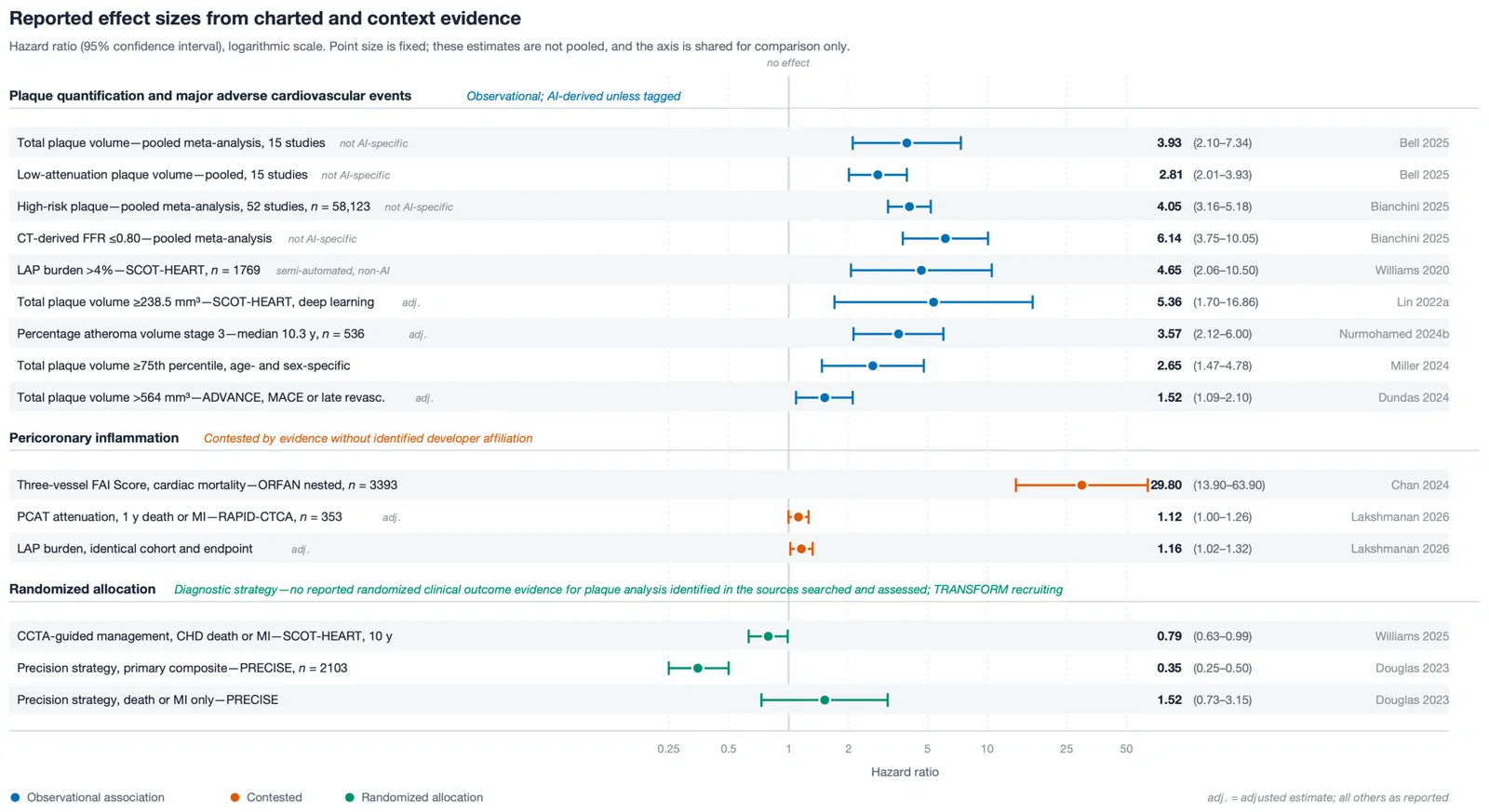
Reported effect sizes from charted and context evidence. Hazard ratios (95% confidence intervals; logarithmic scale) grouped by type of evidence: observational association between quantified plaque or physiology metrics and events (AI-derived unless tagged in the figure as not AI-specific or semi-automated, non-AI); pericoronary inflammation, where studies without identified developer affiliation reach materially different conclusions from the same class of measurement; and randomized allocation, always of a diagnostic strategy rather than plaque-guided management. No reported randomized clinical outcome evidence for plaque analysis was identified in the sources searched and assessed; TRANSFORM is recruiting. FFR-CT estimates, including the PRECISE rows, are context only (Section 2.3). Estimates come from different populations, endpoints and adjustment models and are not pooled; the shared axis permits comparison of magnitude only. Estimates marked “adj.” are adjusted as reported in the source publication. The ORFAN estimate compares an FAI Score in the top versus the bottom quartile in all three coronary arteries, whereas the RAPID-CTCA estimates are per measurement unit, so their magnitudes are not directly comparable. The two PRECISE rows [64] give the same trial’s primary composite and its death-or-myocardial-infarction component. Endpoints are named where they are not major adverse cardiovascular events; the ADVANCE estimate [63] includes late revascularization. CCTA, coronary computed tomography angiography; CHD, coronary heart disease; FAI, fat attenuation index; FFR, fractional flow reserve; LAP, low-attenuation plaque; MI, myocardial infarction; PCAT, pericoronary adipose tissue. Labels in the image give the first author and year of the cited reference (a, b, c or d where first author and year repeat): Williams 2025 [1]; Williams 2020 [3]; Bell 2025 [6]; Bianchini 2025 [7]; Lin 2022a [16]; Lakshmanan 2026 [24]; Chan 2024 [49]; Nurmohamed 2024b [53]; Dundas 2024 [63]; Douglas 2023 [64]; Miller 2024 [70].

Figure 6 assembles the effect sizes underlying these propositions. The observational association between quantified plaque burden (AI-derived or not) and events is large and consistent across cohorts, meta-analyses and measurement conventions. The three randomized estimates all allocated a diagnostic strategy rather than plaque-guided treatment; one, the death-or-myocardial-infarction component of PRECISE, points numerically opposite to the trial’s primary composite [64].

### 5.2. What Still Blocks Routine Use

**Figure 7:**
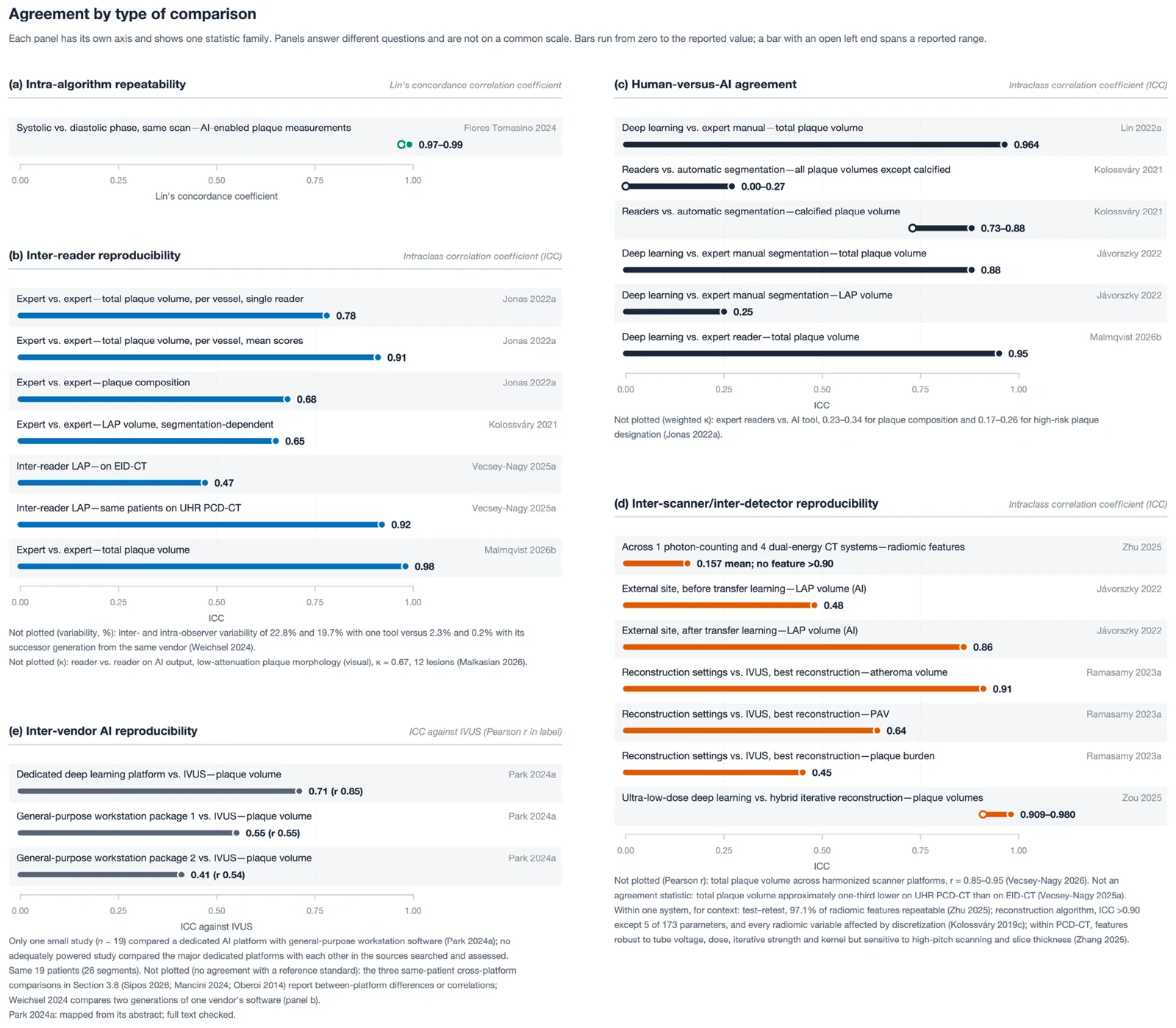
Agreement by comparison type. Each panel shows one comparison type from Table 3 and one family of agreement statistic on its own axis: (**a**) intra-algorithm repeatability; (**b**) inter-reader reproducibility; (**c**) human-versus-AI agreement; (**d**) inter-scanner and detector reproducibility; (**e**) inter-vendor reproducibility. Panels share no common scale, and values should not be compared across them: the intraclass correlation coefficient, weighted κ, Pearson r, Lin’s concordance coefficient and between-platform bias answer different questions. Most favorable AI figures concern repeatability of a fixed algorithm; agreement between expert readers and an AI tool on composition and high-risk plaque was fair [17]; and inter-vendor reproducibility has the least evidence [30,31,68,79,80]. Panel (**e**) shows the intraclass correlation with IVUS plaque volume in the same 19 patients (26 segments): 0.71 for a dedicated deep learning platform and 0.55 and 0.41 for two general-purpose workstation packages, with Pearson r in the labels [79]. Only one small study (*n* = 19) compared a dedicated AI platform with general-purpose workstation software [79] (mapped from its abstract; full text checked); no adequately powered study compared the major dedicated platforms with each other in the sources searched and assessed. The three same-patient cross-platform comparisons [30,31,80] report between-platform differences or correlations rather than agreement with a reference standard and are not plotted, and ref. [68] compares two generations of one vendor’s software (panel (**b**)). EID-CT, energy-integrating detector CT; ICC, intraclass correlation coefficient; LAP, low-attenuation plaque; PCD-CT, photon-counting detector CT; UHR, ultra-high-resolution. Labels in the image give the first author and year of the cited reference (a, b, c or d where first author and year repeat): Lin 2022a [16]; Jonas 2022a [17]; Sipos 2026 [30]; Mancini 2024 [31]; Weichsel 2024 [68]; Park 2024a [79]; Oberoi 2014 [80]; Vecsey-Nagy 2025a [86]; Zou 2025 [90]; Vecsey-Nagy 2026 [94]; Zhu 2025 [103]; Zhang 2025 [104]; Kolossváry 2019c [106]; Kolossváry 2021 [107]; Flores Tomasino 2024 [108]; Malmqvist 2026b [109]; Malkasian 2026 [110]; Jávorszky 2022 [111]; Ramasamy 2023a [112].

**Serial measurement** is an attractive application but the least secure. AI-enabled measurements are stable across systolic and diastolic phases of the same scan (Lin’s concordance 0.97–0.99) [108], but a review of serial Hounsfield-unit-based quantification documents substantial cross-study variability in lipid-rich plaque measurement, questions its reliability for tracking biological transformation and calls for standardized methods and phantom calibration [126]. Expert disagreement on LAP volume segmentation [107] and inter-scanner differences [94,103] compound this uncertainty (Table 3, Figure 7). Grouped by comparison type in Table 3, most favorable AI figures concern repeatability of a fixed algorithm; agreement between expert readers and the AI tool on composition and high-risk plaque was only fair (weighted κ 0.23–0.34 and 0.17–0.26) [17]; and inter-vendor reproducibility has the least evidence [30,31,68,79,80].

**Image quality and analyzability.** Performance in analyzable scans should be distinguished from feasibility in unselected examinations. Most validation studies excluded scans judged non-diagnostic or uninterpretable before AI analysis, often without reporting how many [16,44,65,108], and REVEALPLAQUE enrolled only patients whose scans the same vendor’s pipeline had already processed successfully [60]. Reported analysis failures among enrolled patients were few: 1.3% of 3826 ISCHEMIA participants, with segment-level exclusion of artefact-degraded vessel amounting to 3.1% of total vessel length [54], and 1.6% of 258 in REVEALPLAQUE [60]. By contrast, single-center photon-counting studies excluded 4.5–13.9% of scanned patients for motion, metal or inadequate image quality [88,91]. For CT-derived FFR (outside the scope of this review and cited as context), a prospective multicenter study of a virtual-stenting tool found that agreement with invasive post-PCI FFR did not differ across adjudicated image-quality grades (48.3% excellent, 45% good and 6.7% sufficient; *p* = 0.695), with nitrate dose the only independent factor; two of its authors are affiliated with the manufacturer [127]. Because the cohort contained no examination graded below sufficient, the study describes accuracy across analyzable image quality rather than feasibility in unselected scans. Motion, noise, calcium blooming and suboptimal opacification therefore limit both who can be analyzed and how accurately; published figures largely describe selected analyzable datasets rather than routine practice.

**Decision integration.** The tools output continuous variables, whereas clinicians need decisions. Except for the QCI consensus thresholds [96] and the reporting and indication guidance in the 2025 ACC Scientific Statement [32], the mapping from plaque volume to decision is largely undefined, which makes implementation studies such as AI-DECIDE important [26]. One post hoc analysis proposed a rule-in/rule-out framework for further ischemia testing that combines AI-derived diameter stenosis with percentage atheroma volume above 14.7% and average lumen area below 3.9 mm^2^, derived in CREDENCE and validated in PACIFIC-1 against invasive FFR. It addresses referral for functional testing rather than preventive treatment and has not been tested prospectively [128]; it was identified by the recall check (Section 2.3). The published primary outcomes of AI-DECIDE show the mapping already being made in practice: management changed in 51.3% of 972 symptomatic patients after the analysis was released, graded by the burden stage [27], a change in management, not a clinical outcome. As an observational pre–post cohort, the study characterizes how the output is used rather than testing it against a randomized comparator. Cross-over designs in which clinicians revise decisions after seeing AI output [50,55] likewise cannot distinguish better information from anchoring on the algorithm.

**Surrogate-endpoint status.** CCTA plaque measures are strong candidate surrogate biomarkers for CAD drug development, but formal validation and multi-stakeholder coordination remain outstanding [129].

**Figure 8:**
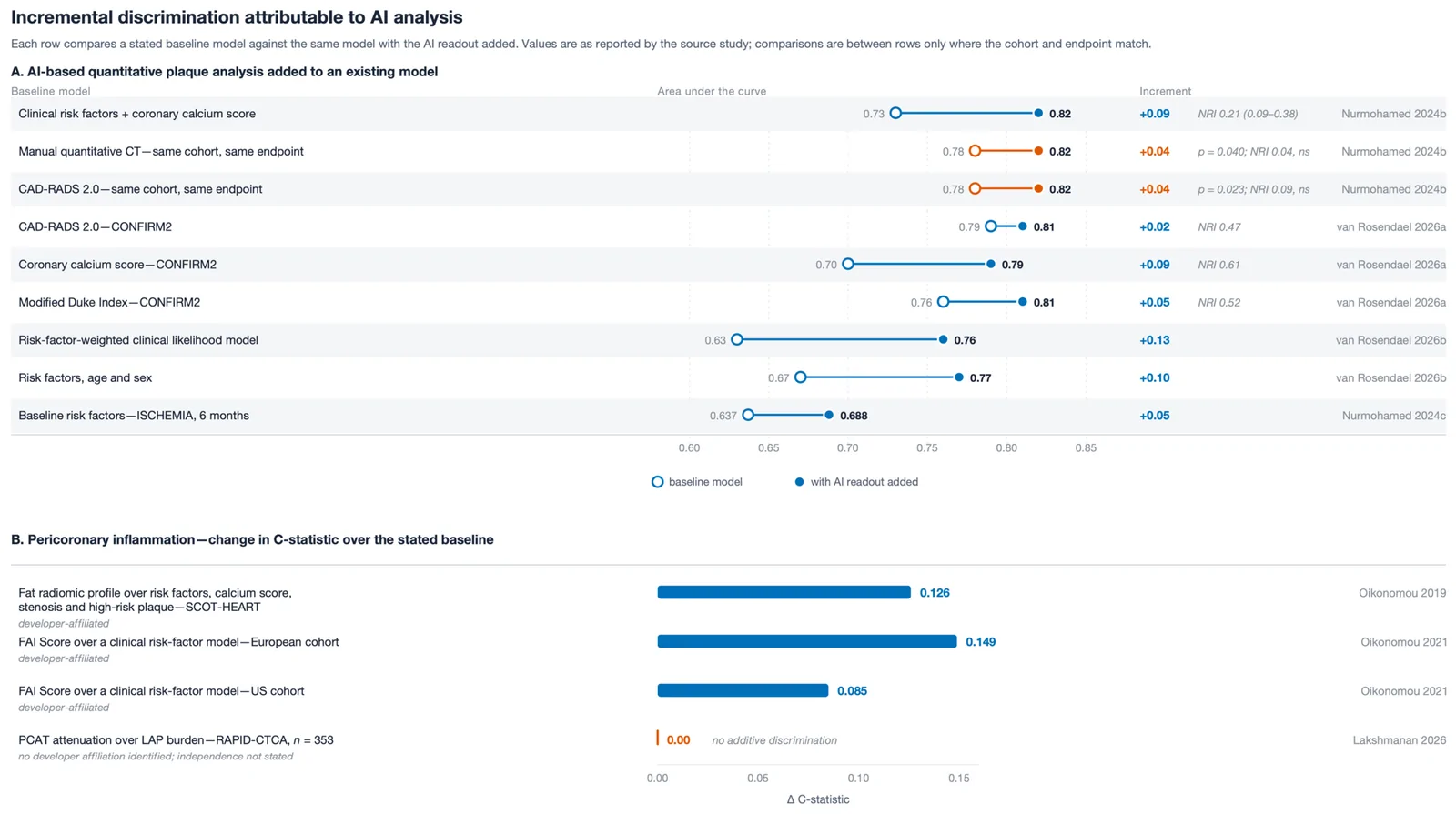
Incremental discrimination attributable to AI analysis. (**A**) Area under the curve for a stated baseline model and for the same model with an AI-derived plaque readout added, with the increment at the right. Against both manual quantitative CT and CAD-RADS 2.0 in the same cohort and for the same endpoint, the area under the curve improved from 0.78 to 0.82 (*p* = 0.040 and *p* = 0.023) while net reclassification reached significance against neither (0.04, 95% CI −0.05 to 0.27; and 0.09, 95% CI −0.02 to 0.29) [53]; both rows are highlighted. (**B**) Change in C-statistic reported for pericoronary inflammation metrics over a stated baseline, annotated by the independence of the analysis from the platform developer. Net reclassification index values are reported as published; the caveats in Section 5.2 apply. AI, artificial intelligence; AUC, area under the receiver operating characteristic curve; CAD-RADS, Coronary Artery Disease Reporting and Data System; FAI, fat attenuation index; LAP, low-attenuation plaque; NRI, net reclassification index; ns, not significant; PCAT, pericoronary adipose tissue. Labels in the image give the first author and year of the cited reference (a, b, c or d where first author and year repeat): Lakshmanan 2026 [24]; van Rosendael 2026a [37]; van Rosendael 2026b [38]; Oikonomou 2019 [47]; Oikonomou 2021 [48]; Nurmohamed 2024b [53]; Nurmohamed 2024c [54].

**Incremental value over what is already available.** The gain attributable to AI analysis depends on the comparator; Figure 8 sets the reported comparisons side by side. Against clinical risk models without anatomical information, the increment is substantial; against CAD-RADS, it is statistically significant but numerically small. Expert manual quantitative CT is the relevant comparator for the claim that AI adds diagnostic information rather than throughput. In the one direct comparison identified, the area under the curve improved from 0.78 to 0.82 (*p* = 0.040), while net reclassification did not reach significance (0.04, 95% CI −0.05 to 0.27) [53]. The net reclassification index, used in both directions in this literature and in this review, has been criticized because it can overstate the incremental value of a new marker [130]; the small differences in area under the curve warrant the same reservation.

### 5.3. Potential Harms and Overtreatment

**Figure 9:**
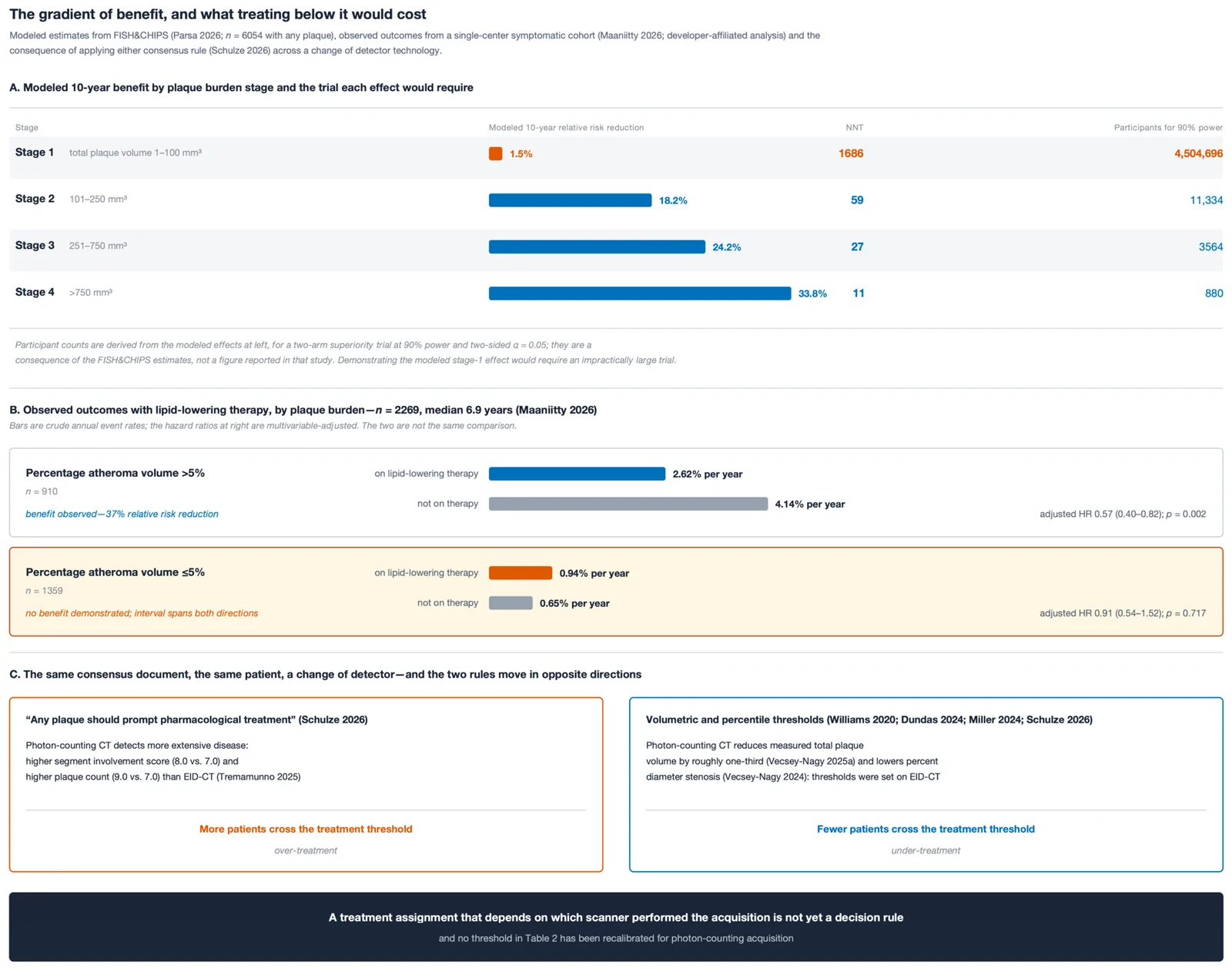
The gradient of benefit and the cost of treating below it. (**A**) Modeled 10-year relative risk reduction and number needed to treat by plaque burden stage, from FISH&CHIPS [118], with the participants a two-arm superiority trial would require to demonstrate each effect at 90% power and two-sided α = 0.05. These counts are derived here from the modeled effects and are not reported in the source study. (**B**) Observed outcomes with and without lipid-lowering therapy, stratified by percentage atheroma volume, in a single-center symptomatic cohort [95] (developer-affiliated analysis). The bars are crude annual event rates, and the hazard ratios are multivariable-adjusted; these are not the same comparison, and in the lower-burden group, they point in different directions. Both are observational and confounded by indication. (**C**) The two thresholds recommended in the same consensus statement [96] move in opposite directions when acquisition changes from energy-integrating to photon-counting detector CT. CAD-RADS, Coronary Artery Disease Reporting and Data System; EID-CT, energy-integrating detector CT; NNT, number needed to treat. Labels in the image give the first author and year of the cited reference (a, b, c or d where first author and year repeat): Williams 2020 [3]; Dundas 2024 [63]; Miller 2024 [70]; Vecsey-Nagy 2024 [83]; Tremamunno 2025 [84]; Vecsey-Nagy 2025a [86]; Maaniitty 2026 [95]; Schulze 2026 [96]; Parsa 2026 [118].

This section goes beyond evidence mapping: it combines published estimates with the authors’ own arithmetic and is offered as interpretation rather than a finding of the review. The sections above do not address the cost of acting on these measurements. A recommendation as broad as the QCI consensus, that any atherosclerotic plaque should prompt pharmacological treatment [96], warrants explicit consideration of that cost. The consensus group, not unanimous on that recommendation, itself anticipated that improving scanners would increasingly detect small plaques in patients with stable chest pain or healthy individuals at low cardiovascular risk and that defining an optimal treatment threshold therefore “might be warranted in the future” [96]. Applied to a symptomatic or screened population, the any-plaque recommendation would place many people on long-term therapy based on a measurement whose portability across vendors, readers and detector technologies has not been demonstrated. Figure 9 sets out the three components of the problem.

The modeled benefit is strongly graded by burden and minimal at the lowest stage. The FISH&CHIPS modeling (*n* = 6054 with any plaque) is a developer-affiliated study; several authors are employees of the platform manufacturer. It grouped total plaque volume into four DECIDE burden stages (1–100, 101–250, 251–750 and >750 mm^3^ for stages 1 to 4, respectively), with treat-to-target low-density lipoprotein cholesterol (LDL-C) goals of <100, <70, <55 and <40 mg/dL. The 10-year relative risk reduction from treat-to-target lipid lowering fell from 33.8% at stage 4 (number needed to treat 11) through 24.2% at stage 3 (number needed to treat 27) to 1.5% at stage 1 (number needed to treat 1686) [118]. These burden stages should not be confused with the AI-DECIDE study (Section 3.3 and Section 5.2). These effects are modeled rather than observed, but the implication for a treat-all rule follows arithmetically: a two-arm superiority trial powered at 90% to demonstrate the stage-1 effect would require about 4.5 million participants, against roughly 3600 for the stage-3 effect (calculation and assumptions in File S3). Under these modeled effects, demonstrating benefit at stage 1, where a treat-all rule would add the most patients, would therefore require an impractically large trial, with sample sizes derived from modeled rather than randomized data.

Where observed outcome data at low burden exist, they do not show a benefit of treatment. In the only cohort identified in the sources searched and assessed that stratifies observed outcomes of lipid-lowering therapy by AI-derived burden (2269 symptomatic patients, median 6.9 years), lipid-lowering therapy was associated with a lower annual event rate among the 910 patients with a percentage atheroma volume above 5% (2.62% versus 4.14%) but not among the 1359 patients at or below 5%. In that lower-burden group, the crude annual event rates were 0.94% in treated patients against 0.65% in untreated, but the multivariable-adjusted hazard ratio was 0.91 (95% CI 0.54–1.52; *p* = 0.717), with a confidence interval wide enough to include both substantial benefit and substantial harm. The source characterizes the two rates as comparably low rather than favoring either arm [95]. In the higher-burden group, by contrast, the crude annual event rate was 2.62% with lipid-lowering therapy against 4.14% without (37% lower), and the multivariable-adjusted hazard ratio was 0.57 (95% CI 0.40–0.82; *p* = 0.002), a 43% lower adjusted hazard. Both comparisons are observational and confounded by indication, and neither is evidence of harm. The lower-burden result therefore shows no demonstrated benefit where a treat-all rule would add the most patients. A related two-center cohort from the same investigators, which may overlap with it, applied the same approach to revascularization in patients referred for CCTA and positron emission tomography (PET) to evaluate coronary disease (*n* = 2233, median 6.8 years); it was identified by the recall check (Section 2.3). Revascularization was associated with fewer deaths, myocardial infarctions or unstable angina only from a per-vessel percentage atheroma volume of 22% upwards (HR 0.50, 95% CI 0.27–0.91), and the patient-level estimate was non-significant; this analysis is also observational and confounded by indication [131].

Reclassification adds a further problem. Without demonstrated portability, a threshold rule would make a patient’s treatment assignment depend partly on which scanner acquired the images. The direction of that dependence is not consistent: the two rules in the same consensus statement move in opposite directions under the same hardware change. Under a volumetric or percentile rule, photon-counting acquisition reduces measured total plaque volume by roughly one-third [86] and lowers percent diameter stenosis [83], moving patients below thresholds derived on energy-integrating detector CT and producing under-treatment relative to the evidence base. Under the any-plaque rule, the same acquisition detects more extensive disease—higher segment involvement and higher plaque count [84]—and would be expected to move patients into treatment who would not have qualified before. A rule whose effect reverses depending on which of its two thresholds is applied, as well as on hardware the patient did not choose, is not yet a reliable decision rule. The same argument applies, with less symmetry, in three further settings: between successive generations of one vendor’s software, whose observer variability differed by an order of magnitude [68]; across vendors, which share no common definitions [75] and whose tools differed systematically in the three small same-patient comparisons identified [30,31,80]; and across readers, whose agreement with automatic segmentation was poor for every plaque component except calcified plaque [107]. Only one small study (*n* = 19) compared a dedicated AI platform with general-purpose workstation software [79] (mapped from its abstract; full text checked); no adequately powered study compared the major dedicated platforms with each other in the sources searched and assessed. The same patient may therefore fall on different sides of a treatment threshold (250 or 564 mm^3^, a percentage atheroma volume stage or a percentile) depending on the platform; before plaque burden serves as a treatment decision rule, thresholds need either cross-platform calibration or platform-specific validation against outcomes.

Two further costs deserve consideration. The first is the downstream cascade: an AI-flagged plaque finding generates further testing, surveillance imaging and the attendant incidental findings and patient anxiety. None of these consequences was quantified for this technology in the sources searched and assessed, and the decision-impact studies in Section 5.1 measured changes in physician intention rather than what happened to patients [50,55]. The second is radiation. In a worldwide survey, the median effective dose for CCTA was 7.4 mSv; only 56% of centers performing it met the ≤9 mSv recommendation, and doses were up to 96% higher in low- and lower-middle-income countries [132]. Any application implying repeat acquisition to track burden inherits that dose, most heavily where it is already highest.

None of this argues against burden-guided prevention. It supports testing such prevention where the modeled benefit is largest and the number needed to treat acceptable, rather than wherever plaque is detectable. Section 9 specifies that trial.

## 6. Regulatory and Validation Considerations

File S5 gives the detailed regulatory discussion. Of the 1016 AI and machine learning (ML) devices authorized by the US Food and Drug Administration (FDA), 277 (27.3%) had cardiology applications; 97.1% were cleared via the 510(k) pathway, and only 3.2% were supported by high-quality clinical trials [133]. Predicate lineage and the reporting of AI/ML use and performance are inconsistent [134,135,136]; frameworks converge on principles rather than evidentiary thresholds [137]. Reporting standards exist but are voluntary [32,43,138,139,140,141,142,143]; weighted mean compliance with CLAIM was 63% [43]. External validation is uncommon [144]. Robustness of stenosis detection to acquisition parameters [145] is not the same as agreement between platforms, for which the evidence is less favorable [68,75,103]. Clearance therefore certifies substantial equivalence to a predicate, not analytical comparability between contemporary devices and not clinical benefit.

## 7. Future Directions

### 7.1. Multimodality Fusion

Plaque morphology, physiology and inflammation are partially independent axes of risk, which argues for fusion rather than substitution. A prospective multicenter study integrating non-contrast T1-weighted MRI high-intensity plaque volume with deep learning-quantified CCTA plaque improved the prediction of periprocedural myocardial injury over CCTA alone (C-statistic 0.880 vs. 0.738), and a hybrid strategy reserving MRI for intermediate-risk lesions retained most of the benefit (0.803 vs. 0.705) [146]. Automated CT-to-CMR fusion pipelines are being developed to co-register coronary anatomy with perfusion territories [147]. Combining AI-derived plaque with hemodynamic analysis improved the prediction of acute coronary syndrome culprit lesions over a CAD-RADS-plus-high-risk-plaque reference model (AUC 0.84 vs. 0.78) [148]. Published work sets out the general case for multimodal medical AI [149], its barriers (data heterogeneity, fusion technique selection, generalization, fairness and safety) [150] and the cardiac-specific obstacles: protocol variability, scarce annotated multimodal datasets, vendor interoperability and limited prospective validation [151]. Digital-twin approaches have also been proposed [152,153,154,155,156], but a systematic review of 42 studies found mostly mechanistic, imaging-personalized models reporting decision making rather than outcome benefits [157]; for coronary plaque, they remain conceptual.

### 7.2. The Health-Economic Case

File S5 gives economic detail. Economic evaluations of CCTA with FFR-CT [158,159,160,161] and the English FFR-CT audit [73] are cited as context only. For AI inflammation analysis, the developer-led model reports strong cost effectiveness [76], whereas the independent assessment finds the evidence insufficient to populate such a model [40]. PCD-CT economics appear more favorable, through avoided downstream testing [162,163]; radiation dose is considered in Section 5.3 [132].

## 8. Strengths and Limitations of This Review

Three features of this review are deliberate. First, excluding vendor materials means that every performance figure reported here has passed peer review, at the cost of omitting claims that may be true but are unverifiable. Second, we report negative findings and evidence not identified in the sources searched and assessed as prominently as positive findings: for example, the non-significant net reclassification of AI over manual quantitative CT [53], the null additive value of pericoronary fat attenuation in a substudy without developer affiliation [24], the poor reproducibility of radiomic features across scanners [103] and indexed validation not identified for one vendor (Section 3.7). Third, where we could not resolve a discrepancy between studies, we report both results.

The limitations follow largely from the same choices. This targeted scoping review used structured but non-exhaustive study selection and, consistent with PRISMA-ScR, applied no formal risk-of-bias instrument across the included literature and performed no quantitative synthesis. The principal procedural limitation is that no step was performed in independent duplicate by two human reviewers: the original selection was an AI title screen under selection rules set by the corresponding author, followed by his evaluation of the 141 records assessed; charting at submission was AI-assisted, and its values were re-checked against sources and the corrections accepted by the corresponding author at this revision; the re-screen used one AI pass over all 1280 records not selected and a blind second AI pass on 175 of them; and the screen of the confirmatory search, the charting of the added records and the abstract mapping of the 307 further records used two independent AI passes, with author decisions only on unresolved items. The original thematic searches were run on a single date (24 July 2026) and were not re-run; each of their queries returned at most its 40 highest-ranked records; and they searched a single database, so records indexed only in Embase, Web of Science or Scopus would not have been retrieved. The uncapped confirmatory search of 15 September 2026 added Europe PMC but no other database (Section 2.2). Records were selected for assessment by an AI agent’s judgement on truncated titles, with no retained prompt, rather than by manual title screening; a protocol-based AI re-screen of all 1280 records not selected, with a blind second AI pass on the 125 flagged records and a random sample of 50 exclusions and with AI-recommended decisions accepted by the corresponding author at the second revision, identified 110 eligible records that this step had missed, and a random sample of 300 gave an estimated 111 (95% CI 73–159) eligible records among the 1280 against the 121 included at submission; eligibility was assessed on title and abstract rather than full text; reasons were not recorded for six of the seven records excluded at that stage; and six records were added by the authors outside the search pipeline (Figure 2). A scoping review maps evidence and identifies gaps; it does not establish the comparative effectiveness of the technologies it describes. Selection and emphasis therefore involve some interpretive judgement that a systematic protocol would constrain.

Reliance on the published record has an asymmetric consequence in this field. Developer-affiliated studies dominate the prognostic literature for every commercial platform in Section 3, and negative internal validations are unlikely to reach publication. The evidence base we characterize is therefore probably more favorable to these tools than the totality of evidence about them. Both instances in which groups without developer affiliation examined a developer-derived claim [24,40] returned substantially weaker results than the originating work, although for [24], the disclosure statement could not be accessed; the sample is small but consistent. A third cohort (*n* = 564, median 3.2 years), identified by the recall check (Section 2.3), reported a stronger association but is not independent: its authors include an employee and a founder of the developer. In that cohort, CaRi-Heart risk ≥ 5% was independently associated with a patient-oriented composite of death, myocardial infarction or any revascularization (adjusted HR 2.95), irrespective of high-risk plaque and invasive FFR. However, that endpoint includes revascularization, and the developer’s cloud service computes the risk score itself [78].

The field is also moving faster than the review cycle: our search closed on 24 July 2026, when the AI-DECIDE program [26] and the randomized trial called for by the REVOLUTION investigators [117] were both unreported; either could materially change the conclusions in Section 9. Final pagination, and in some cases final analyses, may differ for several cited records published online ahead of print. The confirmatory search (Section 2.2) returned 3785 records not returned by the original search. They were screened at this revision by two independent AI passes, and the corresponding author decided the 335 records the passes did not settle by accepting written agent recommendations, not record by record and not blind to them (Section 2.3). That screen has its own limitations: both passes used the same model under the same instruction, so errors common to both cannot be detected by their agreement; the only check on the 3054 shared exclusions is an AI re-read of 100 of them (0 eligible; 95% CI 0–3.6%), not a human audit; blinding between the passes was self-reported; and 307 of the 308 new eligible records it added were mapped from their abstracts only, not fully charted. Together, the 236 charted and 307 mapped records are therefore a structured, broader but still not exhaustive sample of the indexed literature; statements that evidence was not identified apply only to the sources searched and the records assessed and, for the mapped records, to what their abstracts report. Of the 236 charted records, 115 were charted from the abstract and disclosure fields alone (Table S1, column Q), so the same limit applies to part of the charted set. At the first revision, the most important absence statements had also been tested directly with targeted PubMed, Europe PMC and trial-registry searches (randomized outcome trials, cross-vendor comparisons, validation of one vendor’s plaque tool, outcome stratification by AI-derived burden and photon-counting threshold recalibration); two proved wrong and were corrected (Section 3.8, Section 5.1 and Section 9.1). The evidence map remained stable when the charted dataset approximately doubled and when the mapped records were added (File S6).

To test whether the re-screen records alter the evidence map, we charted all of them and, with the analysis specified before they were scored, re-scored Figure 1 with the same rule (File S6), comparing (a) the figure published at the first revision, (b) the 121 records charted at submission under the corrected charting of this revision, (c) the 235 records after adding the 110 re-screen records and the four records added from the confirmatory search, (d) 236 records and (e) 543 records. At the published threshold of 200 participants, the 235 records reproduced the published figure in every cell, including when only records that both screening passes called eligible were added. Relative to the corrected 121-record baseline, two cells changed: plaque burden at stage 3 moved from limited to established, developer-affiliated, through the DECIDE registry [27], one of the four confirmatory-search records; and pericoronary inflammation at stage 2 moved from established, developer-affiliated, to contested because an additional independent three-center cohort of 777 patients found that pericoronary attenuation did not improve prediction, although a radiomic model of the same fat did [25]. In the 121 records, that cell is not contested because the disclosure statement of the original contesting study [24] could not be accessed, and its independence is charted as not stated at this revision. At 500 participants, the cell is contested in the 235, 236 and 543 records, whereas the published figure marked it as changing to established at that threshold. In most cells, the added records contributed evidence below the Figure 1 bar (for example, 14 additional analytical validations of plaque burden); three further prognostic studies of plaque burden met the bar, all developer-affiliated (File S6). The added records contained no further between-platform comparison and no randomized outcome study; most concerning a named commercial platform were developer-affiliated (for example, 24 of the 30 attributed to AI-QCT). We then applied the same rule to the screened confirmatory-search records (File S6). One, the general-population cohort [11], met the bar for plaque composition at stage 2 when charted from its full text (six centers; prespecified analysis plan; 23,314 participants; noncalcified plaque volume quantified with academic software developed by three of the authors [109]; no commercial developer affiliation disclosed), so the developer marker on that cell was removed (236 records; Figure 1). Its published analysis centers on total plaque volume and reports similar results for noncalcified plaque volume. Adding the other 307 abstract-mapped records (543 records in all) changed no further cell or developer marker at 100, 200 or 500 participants, whether all were added or only the 238 that both screening passes included. The mapped records contained no randomized outcome study, no observed decision-impact study and no photon-counting prognostic study. Abstracts rarely report a prespecified endpoint or the number of centers, so this result partly reflects the limits of abstract-level mapping. At the published threshold, one further cell (radiomics at stage 1) could change if the full texts of two histology- or outcome-referenced radiomics studies, neither of which could be retrieved through open-access interfaces, showed design criteria that their abstracts do not state. The mapped records also include one further between-platform comparison of plaque measurement [79] (Section 3.8).

Finally, we had no access to primary data, source code or training datasets for any commercial platform discussed and could not independently verify the reported measurements. The review characterizes what the literature claims and how well those claims are supported; it is not a validation study.

## 9. Conclusions

### 9.1. Summary of Evidence

The evidence base for AI-assisted coronary plaque characterization has matured substantially. Platforms show strong analytical agreement with IVUS and OCT and one also with histology in carotid specimens. Plaque burden is associated with MACEs in symptomatic or clinically referred patients across registries, randomized-trial substudies and meta-analyses; this is a prognostic property of the measurement, AI-derived or manual, not evidence that AI itself improves prediction or outcomes. Radiomic phenotyping added prognostic information beyond volumetric measures in one large cohort, with a signal that strengthened over long follow-up.

Three qualifications apply, summarized in Figure 1. First, AI’s demonstrated advantage over expert human quantification in prognostic discrimination is small: where compared directly, the gain over manual quantitative CT was statistically significant but numerically small (0.82 versus 0.78), and net reclassification was not significant [53]; gains over CAD-RADS are likewise modest [37]. AI’s consistent advantages are speed and reproducibility. One prospective study, identified by the recall check (Section 2.3), trained a deep learning model on co-registered NIRS-IVUS and reported closer agreement with the invasive reference than conventional expert CCTA analysis for total and percentage atheroma volume in a test set of 48 vessels [164]; this accuracy advantage has been reported but not replicated. For the narrower task of detecting ≥ 50% stenosis, as distinct from plaque quantification, a meta-analysis of 17 mostly retrospective studies, also identified by the recall check, reported higher pooled discrimination for AI than for radiologists (AUC 0.96 versus 0.91), amid heterogeneity in CT technology [165]. Second, no randomized trial allocating patients to AI-based plaque analysis versus standard care and reporting clinical endpoints was identified in the sources searched and assessed; every published outcome claim rests on post hoc analyses, registries or simulation. AI-derived CCTA plaque measures therefore carry prognostic information in symptomatic patients, but randomized evidence that AI-CCTA-guided treatment reduces myocardial infarction or cardiovascular death in asymptomatic adults, or does so better than CAC-guided prevention, has not been established in the sources searched and assessed (Section 5.1). These absence statements also cover the 307 records mapped from abstracts only, but only at the abstract level: evidence reported only in a full text would not have been captured (Section 2.2 and Section 8). One general-population cohort without prior atherosclerotic disease, added from the confirmatory search and charted from its full text, reported that deep-learning-quantified plaque volume improved the prediction of coronary death or myocardial infarction beyond SCORE2 [11]. That is evidence of improved prediction in that population, not of a benefit from measurement-guided treatment, and no randomized comparison of AI-guided with CAC-guided prevention was identified in the sources searched and assessed. Improved prediction, changes in prescribing and improved clinical outcomes are distinct findings: the charted evidence supports improved prediction and includes observational or intention-based evidence of prescribing changes [27,49,55] but contains no randomized evidence of improved clinical outcomes. The first such trial, TRANSFORM (ClinicalTrials.gov NCT06112418), randomizes a planned 7500 patients to preventive care guided by AI-based plaque staging or to risk-factor-based care, with a cardiovascular-event primary endpoint, and has been recruiting since March 2024. AI-DECIDE is an observational cohort (ClinicalTrials.gov NCT06376851) [26,74] whose published primary outcomes measure change in management, not clinical events [27]. Third, measurement portability has not been demonstrated: between successive generations of one vendor’s software [68]; across vendors, where three small same-patient comparisons, one including a non-commercial prototype, documented systematic between-platform differences that are largest for low-attenuation plaque where it was assessed [30,31,80] and where vendors share no definitions or validation datasets [75]; across readers for compositional metrics [17,107]; and across detector technologies, where PCD-CT shifts plaque volumes by roughly a third and reclassifies about half of patients [83,84,86]. Only one small study (*n* = 19) compared a dedicated AI platform with general-purpose workstation software [79] (mapped from its abstract; full text checked); no adequately powered study compared the major dedicated platforms with each other in the sources searched and assessed. Interchangeability therefore cannot be assumed: without an adequately powered same-patient comparison in the sources searched and assessed, the size and clinical consequences of disagreement between the major commercial platforms have not been established. Because of the detector shift, no threshold in Table 2 can be assumed to transfer to photon-counting acquisition without recalibration. A patient may fall on different sides of a treatment threshold depending on the platform, so cross-platform calibration or platform-specific, outcome-validated thresholds are needed before plaque burden is used as a decision rule.

The most defensible near-term proposition, to be tested prospectively rather than adopted, is therefore not autonomous decision making but standardized, rapid, reproducible plaque quantification used to intensify preventive therapy in patients undergoing clinically indicated CCTA whose burden is high but whose stenosis is not, the population in which most events occur and which currently receives least attention [49,95,96]. Realizing it requires outcome-anchored randomized evidence, cross-platform standardization of the kind the QIBA Profile proposes [75], threshold recalibration for photon-counting systems [94] and regulatory reporting more transparent than the 510(k) record to date [133].

### 9.2. Proposed Research Agenda

The first need is an independent, vendor-neutral evaluation of the major AI-CCTA platforms on the same examinations, assessing absolute agreement, systematic bias, calibration, plaque-stage and treatment-threshold reclassification and prognostic discrimination against outcomes. A large population cohort with CCTA and outcome follow-up would suit this, and professional-society guidance on quantitative plaque analysis [32] should call for it explicitly.

**Figure 10:**
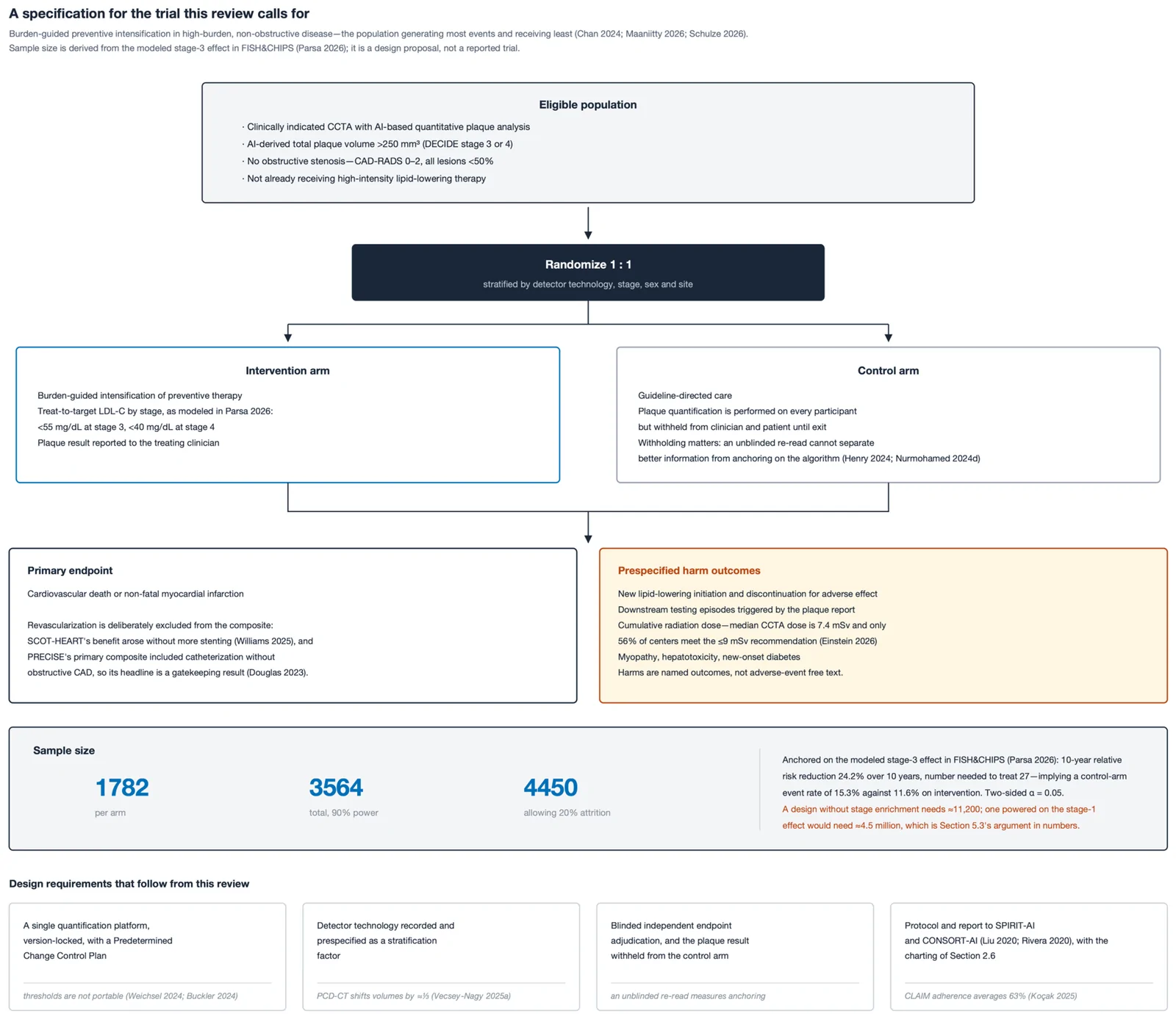
The authors’ proposed specification for the outcome-anchored randomized trial this review calls for. Eligibility, allocation, endpoint structure, prespecified harm outcomes and sample size for a two-arm superiority trial of burden-guided preventive intensification in high-burden, non-obstructive coronary disease. Sample size is derived from the modeled stage-3 effect in FISH&CHIPS [118], a developer-affiliated modeling study (see text and File S3); the schema is a design proposal and not a registered or reported trial. CAD-RADS, Coronary Artery Disease Reporting and Data System; CCTA, coronary computed tomography angiography; CONSORT-AI, Consolidated Standards of Reporting Trials—Artificial Intelligence extension; LDL-C, low-density lipoprotein cholesterol; PCD-CT, photon-counting detector CT; SPIRIT-AI, Standard Protocol Items: Recommendations for Interventional Trials—Artificial Intelligence extension. Labels in the image give the first author and year of the cited reference (a, b, c or d where first author and year repeat): Williams 2025 [1]; Koçak 2025 [43]; Chan 2024 [49]; Henry 2024 [50]; Nurmohamed 2024d [55]; Douglas 2023 [64]; Weichsel 2024 [68]; Buckler 2024 [75]; Vecsey-Nagy 2025a [86]; Maaniitty 2026 [95]; Schulze 2026 [96]; Parsa 2026 [118]; Einstein 2026 [132]; Liu 2020 [138]; Rivera 2020 [139].

The proposition in Section 9.1 is testable. What follows is the authors’ proposal, derived from the evidence reviewed above, rather than a finding of the review. Figure 10 specifies the proposed trial. The population is that identified above: patients undergoing clinically indicated CCTA whose AI-derived total plaque volume exceeds 250 mm^3^ but who have no obstructive stenosis and who are not already on high-intensity lipid-lowering therapy. The 250 mm^3^ threshold, the lower bound of stage 3 in the cited model [118], was defined on one platform and one detector technology and, like every threshold in Table 2, would need recalibration before use on another platform or detector. In ORFAN, two-thirds of major adverse cardiac events and 63.7% of cardiac deaths occurred in patients without obstructive coronary artery disease [49]. Participants are allocated 1:1 to burden-guided intensification of preventive therapy, using the stage-specific treat-to-target LDL-C thresholds modeled in FISH&CHIPS [118], or to guideline-directed care, in which plaque quantification is performed for every participant but withheld from the treating clinician and patient. Withholding is necessary because cross-over designs cannot distinguish better information from anchoring on the algorithm [50,55].

The primary endpoint should be cardiovascular death or non-fatal myocardial infarction, not including revascularization. SCOT-HEART’s 10-year composite was driven by fewer non-fatal myocardial infarctions (HR 0.72) with no reduction in all-cause, cardiovascular or coronary heart disease death and no increase in coronary revascularization [1]. PRECISE’s primary composite combined death and non-fatal MI with catheterization without obstructive coronary disease, so its headline hazard ratio of 0.35 records a gatekeeping rather than a survival gain; the death-or-MI component was numerically higher [64]. Harms belong in the protocol as named outcomes rather than adverse-event free text: initiation and discontinuation of lipid-lowering therapy, downstream testing episodes triggered by the plaque report, cumulative radiation dose and the recognized metabolic and musculoskeletal effects of intensified therapy.

The sample size is anchored on the modeled stage-3 estimates in FISH&CHIPS [118]: for stage 3, the second highest of four burden stages, a 10-year relative risk reduction of 24.2% and a number needed to treat of 27 imply a control-arm event rate of 15.3% against 11.6% on intervention. The trial would then require 1782 participants per arm, or 3564 in total (File S3), for 90% power at two-sided α = 0.05 over a 10-year horizon and approximately 4450 allowing for 20% attrition. Stage enrichment makes this feasible: without it, the same design requires roughly 11,200 participants. Three design constraints follow directly from this review’s findings. The trial must use a single, version-locked quantification platform under a Predetermined Change Control Plan because thresholds have not been shown to transfer between platforms [68,75]; record detector technology and prespecify it as a stratification factor because photon-counting acquisition shifts measured volumes by roughly a third [86]; and be protocolled and reported to the Standard Protocol Items: Recommendations for Interventional Trials–Artificial Intelligence (SPIRIT-AI) and Consolidated Standards of Reporting Trials–Artificial Intelligence (CONSORT-AI) extensions [138,139] because adherence to the CLAIM checklist across medical-imaging AI averages 63% [43]. These figures are a design proposal derived from modeled effects, not a reported trial, and a modeled relative risk reduction is not a guarantee of an observed one. Nevertheless, in our view, the lack of such a reported trial, rather than any dispute about the measurements, currently separates this technology from clinical adoption.

## Figures and Tables

**Table 1:** Commercial platforms for AI-assisted coronary plaque characterization: reference standards and published evidence. Platforms are listed alphabetically by vendor. The table is not a ranking: the supporting studies differ in population, reference standard, endpoint, design and independence from the developer, so rows are not directly comparable.

Platform (Vendor) b	Analytical Reference Standard	Largest Analytical Validation	Prognostic Evidence	Decision-Impact Evidence a	Randomized Outcome Trial	Principal Limitation of the Evidence Base
Artrya Attribution by author affiliation only	CAD-RADS category concordance and QCA-derived stenosis [44]	769 patients/3012 vessels; 93.5% per-vessel agreement within one CAD-RADS category; per-vessel AUC 0.897 for ≥50% stenosis [44]	None	None	None	High-risk-plaque test set of 45 patients, AUC 0.77–0.80—underpowered for the compositional claim; calcium scoring [45] and perioperative risk [46] validated separately; product name absent from indexed abstracts
Caristo (CaRi-Heart) Named in publications	Adipose tissue gene expression for inflammation, fibrosis and vascularity [47]: not an imaging or histological plaque reference	Radiotranscriptomic derivation with SCOT-HEART validation, ΔC-statistic 0.126 [47]; standardized FAI-Score mapping in 3912 US and European patients, ΔC-statistic 0.085 and 0.149 [48]	ORFAN (unselected patients undergoing clinically indicated CCTA): two-thirds of MACE occurred in patients without obstructive CAD in the full cohort (*n* = 40,091, eight UK hospitals); in a nested cohort of 3393 from two hospitals, cardiac mortality HR 29.8 (95% CI 13.9–63.9) for an FAI Score in the top versus the bottom quartile in all three vessels [49]	Single-center *n* = 164: about half reclassified, management changed in a third, no outcome data [50]; in an ORFAN survey of 744 patients undergoing CCTA for chest pain, making the AI risk classification available was reported to change management in 45% [49]	None	Contested. Independent NIHR assessment found one includable study, high risk of bias by PROBAST, and no studies reporting clinical effects or costs [40]; RAPID-CTCA substudy *n* = 353: diagnostic C-statistic 0.57 for ACS and no prognostic gain over LAP burden (ΔC-statistic 0.00) [24]
Circle Cardiovascular Imaging	—	No peer-reviewed coronary plaque characterization validation study identified by product or company name searching; the software is used as the measurement tool in single-center CCTA plaque and pericoronary-fat studies without reported validation (Section 3.7)	—	—	—	Recorded as indexed validation not identified in the sources searched and assessed, rather than as an omission; no claim is made about performance
AI-QCT (Cleerly) Named in publications	Blinded core-lab QCA and invasive FFR [51]; IVUS [52]	INVICTUS: 108 vessels in 85 patients, independent blinded core labs; r = 0.83 plaque, 0.94 lumen, 0.90 external elastic membrane and 0.96 calcium index; sponsored by the manufacturer; analysis at its own core laboratory [52]	Plaque staging, 536 patients referred for suspected CAD, median 10.3 y: AI-QCT stage 3 PAV adj. HR 3.57; 10 y AUC 0.82 vs. 0.73 (clinical risk factors + calcium score) [53]; CONFIRM2 (*n* = 1916; asymptomatic patients excluded): AUC 0.81 vs. 0.79 and NRI 0.47 versus CAD-RADS 2.0 [37]; companion *n* = 3551, symptomatic: AUC 0.63 → 0.76 [38]. ISCHEMIA (chronic coronary disease with moderate or severe ischemia) *n* = 3711: AUC 0.688 vs. 0.637 [54]	CERTAIN cross-over, 750 patients: intended management changed in 57.1%, projected testing −37.1%, intended statin initiation or intensification +28.1% [55]; modeled 39–49% reduction in invasive angiography [56]	None reported. TRANSFORM (NCT06112418) randomizes Cleerly stage-based care versus risk-factor-based care; *n* = 7500 planned, cardiovascular-event primary endpoint, recruiting since March 2024, primary completion projected 2029	10 y AUC over manual quantitative CT 0.82 vs. 0.78, *p* = 0.040, but NRI 0.04 (95% CI −0.05 to 0.27), non-significant [53]; ISCHEMIA gain described by the authors as “modest” [54]; decision-impact data are unblinded re-assessment of stated intent
Elucid Named in publications	Histology: carotid endarterectomy specimens with paired CTA [33]; 576 tissue samples [34]	Weighted κ 0.82 versus expert pathologists, AUC 0.95–0.99, against diameter stenosis κ 0.25 [33]; near-unity regression slopes for LRNC, intraplaque hemorrhage, calcification and matrix [34]	None identified in the sources searched and assessed (no MACE-anchored study)	Treatment-response detection only: EVAPORATE substudy *n* = 55 [57]; observational psoriasis cohort *n* = 209, ΔLRNC −0.22 mm^2^ [58]	None	The platform with the strongest tissue-level anchoring has no prognostic evidence identified in the sources searched and assessed; response cohorts are small, and reader reproducibility was assessed in 16 patients [59]
HeartFlow Named in publications	IVUS (AI-QCPA plaque tool) [60,61]	REVEALPLAQUE: prospective, 15 centers, 237 patients/432 lesions; r = 0.91 total and calcified, 0.87 non-calcified; little Bland–Altman bias [60]; smaller single-center study: 33 patients/67 vessels; ρ = 0.92 total, 0.91 non-calcified, 0.87 calcified; total plaque volume underestimated by 9.4 mm^3^ [61]	ADVANCE, retrospective analysis, *n* = 4430 (population not stated in this report; the parent registry enrolled patients with suspected CAD [62]): total plaque volume > 564 mm^3^, adj. HR 1.52 for MACE or late revascularization, incremental to stenosis and FFR-CT [63].	DECIDE registry, 972 symptomatic patients: management changed in 51.3% after release of the plaque analysis; observational, no concurrent control [27]	None for the plaque tool; PRECISE [64] randomized an FFR-CT-based strategy c	Plaque-tool validation is developer-affiliated (seven REVEALPLAQUE authors are manufacturer employees) [60]; decision-impact data are observational and uncontrolled [27]
Vendor-unattributed and academic pipelines	OCT [65]; IVUS and expert readers, including a photon-counting test set [66]	OCT: 91 patients, 153 co-registered lesions, r = 0.84, 1.8 min/patient [65]; 17-hospital development with four external test sets, all ICCs > 0.90 [66]	Moderate only: C-index 0.64–0.74; population not stated in the abstract [66]	Reader assistance in heavily calcified vessels: specificity improved 7–11%, better inter-reader agreement, multicenter stepwise design [67]	None	Wide Bland–Altman limits of agreement despite high ICCs [66]; head-to-head evaluation of two tools—successive generations from one manufacturer, 50 patients—found inter- and intra-observer variability of 22.8% and 19.7% versus 2.3% and 0.2%, with a two-fold difference in processing time [68]

a “Decision-impact evidence” denotes studies measuring change in physician intention or downstream test utilization, not clinical events. b Attribution basis is given beneath each platform name, following the order in Section 2.4. c PRECISE randomized a diagnostic strategy incorporating selective FFR-CT, not allocation to AI plaque analysis; it is the only randomized trial in this table and does not test the plaque tool. FFR-CT is outside this review’s scope, and PRECISE is cited as context. “None” denotes none identified in the sources searched and assessed. Of the 115 records charted at revision, those attributed to a platform (Section 2.4; File S6): AI-QCT (Cleerly) 30 (24 developer-affiliated, 3 partial, 2 independent and 1 not stated; neither independent record is a prognostic, decision-impact or randomized study); HeartFlow 5 and Caristo 6 (all developer-affiliated); Elucid 2 (no prognostic study); Artrya and Circle none. Another vendor’s pericoronary-fat software (Shukun) was used in three independent prognostic studies, one of them [25]. adj., adjusted; AUC, area under the receiver operating characteristic curve; CAD, coronary artery disease; CAD-RADS, Coronary Artery Disease Reporting and Data System; FAI, fat attenuation index; FFR, fractional flow reserve; FFR-CT, CT-derived fractional flow reserve; ICC, intraclass correlation coefficient; IVUS, intravascular ultrasound; LAP, low-attenuation plaque; LRNC, lipid-rich necrotic core; MACE, major adverse cardiovascular event; NRI, net reclassification index; OCT, optical coherence tomography; PAV, percentage atheroma volume; QCA, quantitative coronary angiography; ρ, Spearman correlation coefficient.

**Table 2:** Prognostic and treatment thresholds in current use, with the population and conditions under which each was derived.

Threshold	Metric	Derived in (*n*)	Platform	Detector	Endpoint	Effect at Threshold	Ref.
>4%	LAP burden	SCOT-HEART	Autoplaque (semi-automated; not AI-based)	EID-CT	Fatal or non-fatal MI	≈5-fold risk; HR 1.60 per doubling	[3]
>238.5 mm^3^	Total plaque volume	SCOT-HEART substudy, stable chest pain (1611)	Research deep learning system	EID-CT	Fatal or non-fatal MI	adj. HR 5.36 (1.70–16.86)	[16]
>564 mm^3^	Total plaque volume	ADVANCE (4430); population not stated in this report (parent registry: suspected CAD [62])	AI-QCPA (HeartFlow)	EID-CT	MACE or late revascularization	adj. HR 1.52	[63]
AI-QCT stage 3	Percentage atheroma volume	Cohort referred for suspected CAD (536), median 10.3 y	AI-QCT (Cleerly)	EID-CT	MACE, median 10.3 y	adj. HR 3.57	[53]
>5%	Percentage atheroma volume	Symptomatic cohort (2269)	AI-assisted quantitative CCTA, vendor not named	EID-CT	Benefit from lipid-lowering therapy	Benefit above; none at ≤5%	[95]
Any plaque	Presence	Consensus (DISCHARGE, SCOT-HEART percentiles)	AI-assisted quantitative CCTA	EID-CT	Pharmacological treatment	Consensus, not outcome-derived	[96]
70th percentile	Total plaque volume	Consensus, as above	AI-assisted quantitative CCTA	EID-CT	High-intensity treatment	Consensus, not outcome-derived	[96]
≥75th percentile	Total plaque volume, age- and sex-specific	Multicenter percentile derivation (with and without symptoms; outcomes tested in symptomatic samples)	Research deep learning system	EID-CT	MI	HR 2.65 unadjusted; adj. 2.38–2.50	[70]
Three-vessel elevation	FAI Score	ORFAN nested cohort, unselected clinically indicated CCTA (3393), median 7.7 y	CaRi-Heart (Caristo)	EID-CT	Cardiac mortality	HR 29.8 (13.9–63.9), top vs. bottom quartile in all three vessels	[49]
No usable threshold	PCAT attenuation	RAPID-CTCA substudy, suspected ACS (353)	Not named in the primary publication	EID-CT	Diagnosis of ACS	C-statistic 0.57 for diagnosis; 0.63 for 1 y death or MI, ΔC 0.00 over LAP	[24]

Every threshold in this table was derived on energy-integrating detector CT; photon-counting acquisition and reconstruction shift measured volumes and disease categories (Section 4.1, Figure 4) [82,83,84,86]. No study recalibrating any threshold in this table against outcomes or a reference standard for photon-counting acquisition was identified in the sources searched and assessed; published photon-counting work adapts segmentation thresholds to energy level, reconstruction kernel or acquisition settings [92,94] rather than re-anchoring a prognostic cut-point. Volumetric thresholds are also platform-specific: three small same-patient comparisons, one including a non-commercial prototype, documented systematic between-platform differences in plaque volume, largest for low-attenuation plaque where it was assessed [30,31,80], and a fourth, smaller comparison of a deep learning platform with two general-purpose workstation packages pointed the same way [79] (mapped from its abstract; full text checked). None was designed to test interchangeability or threshold transfer, no adequately powered comparison of the major commercial platforms was identified, and the lack of shared definitions, metrics and validation datasets is documented [75]. Where the primary publication does not name the quantification software, the table records this rather than inferring a platform, following the attribution hierarchy in Section 2.4. adj., adjusted; EID-CT, energy-integrating detector CT; FAI, fat attenuation index; HR, hazard ratio; LAP, low-attenuation plaque; MACE, major adverse cardiovascular event; MI, myocardial infarction; PCAT, pericoronary adipose tissue.

**Table 3:** Reproducibility of coronary plaque and radiomic measurements, grouped by comparison type.

Comparison Type	Source of Variation	Metric Affected	Agreement Achieved	Ref.
Intra-algorithm repeatability	Systolic vs. diastolic phase, same scan	AI-enabled plaque measurements	Lin’s concordance 0.97–0.99	[108]
	Test–retest, same system	Radiomic features	97.1% of features repeatable	[103]
	Discretization choice	Every radiomic variable	All affected	[106]
Inter-reader reproducibility	Expert reader vs. expert reader	Total plaque volume, per vessel	ICC 0.78 single reader; 0.91 mean scores	[17]
	Expert reader vs. expert reader	Plaque composition	ICC 0.68 (moderate)	[17]
	Expert reader vs. expert reader	LAP volume, segmentation-dependent	ICC 0.65 (fair)	[107]
	Expert reader vs. expert reader †	Total plaque volume	ICC 0.98; limits of agreement −73 to 82 mm^3^	[109]
	Reader vs. reader, on AI output †	Low-attenuation plaque morphology (visual)	κ 0.67, 12 lesions	[110]
Human-versus-AI agreement	Deep learning vs. expert manual	Total plaque volume	ICC 0.964; 5.7 s vs. 25.7 min	[16]
	Expert reader vs. AI tool	Composition; high-risk plaque designation	Weighted κ 0.23–0.34 and 0.17–0.26 (fair)	[17]
	Human vs. automatic segmentation (QAngioCT, Medis; automatic, not described as AI)	All plaque volumes except calcified	ICC 0.00–0.27 (poor); calcified plaque 0.73–0.88 (good)	[107]
	Deep learning vs. expert manual segmentation †	Total; low-attenuation plaque volume	ICC 0.88; 0.25	[111]
	Deep learning vs. expert reader †	Total plaque volume	ICC 0.95; bias −8.35 mm^3^, limits of agreement −102 to 85 mm^3^	[109]
Inter-scanner and detector reproducibility	Reconstruction algorithm	Volumetric and radiomic metrics	ICC > 0.90 except 5 of 173 parameters	[106]
	EID-CT vs. UHR PCD-CT	Total plaque volume	≈one-third reduction	[86]
	EID-CT vs. UHR PCD-CT	LAP, inter-reader	ICC 0.47 → 0.92 (improved)	[86]
	Across harmonized scanner platforms (single institution; manufacturer prototype software)	Total plaque volume	r = 0.85–0.95	[94]
	Within PCD-CT: tube voltage, dose, iterative strength, kernel	Radiomic features	Robust	[104]
	Within PCD-CT: high-pitch scanning, slice thickness	Radiomic features	Brittle	[104]
	Across scanner systems (1 PCD-CT and 4 dual-energy CT)	Radiomic features	Mean ICC 0.157; no feature > 0.90	[103]
	External site, before vs. after transfer learning †	Low-attenuation plaque volume (AI)	ICC 0.48 → 0.86	[111]
	Reconstruction kernel, slice thickness and iterative strength, against IVUS †	Atheroma volume; PAV; plaque burden	ICC 0.91; 0.64; 0.45 (best reconstruction)	[112]
	Within PCD-CT: standard vs. artefact-correcting reconstruction †	Total plaque volume	111 vs. 75 mm^3^; not interchangeable	[113]
	Ultra-low-dose deep learning vs. hybrid iterative reconstruction †	Necrotic, fibrous, calcified plaque volume	ICC 0.909–0.980	[90]
	Tube voltage and pixel spacing †	Pericoronary adipose tissue attenuation	Shifted by 8 HU at 120 kVp and by pixel spacing	[114]
Inter-vendor reproducibility (and between software versions)	Two software generations of one manufacturer, head-to-head	Inter- and intra-observer variability	22.8%/19.7% vs. 2.3%/0.2%	[68]
	Two platforms, same 24 segments	Plaque components, particularly LAP	Within-platform R ≥ 0.819; between-platform agreement less good, poorest for LAP	[31]
	Two vendors’ tools, same patients (one a non-commercial prototype)	Total plaque volume	Median 67.0 vs. 50.8 mm^3^; mean bias 45.3 mm^3^ (EID-CT), 15.0 mm^3^ (PCD-CT)	[30]
	Deep learning platform vs. two general-purpose workstation packages, same 19 patients (26 segments), IVUS reference ‡	Plaque volume against IVUS	Pearson r 0.85 vs. 0.55 and 0.54; ICC 0.71 vs. 0.55 and 0.41	[79]

The pattern is consistent: agreement is excellent for gross volumetric measures on a fixed platform, markedly lower for compositional and textural measures and lower again across platforms. Statistics should not be compared across comparison-type groups. Rows from records added at revision are marked †; the 110 records added from the re-screen contained no further inter-vendor comparison (File S6). The row marked ‡ was mapped from its abstract; its central-claim fields were checked against the full text (File S7, sheet E). This is the technical basis for the portability claim in Section 9. EID-CT, energy-integrating detector CT; ICC, intraclass correlation coefficient; LAP, low-attenuation plaque; PCD-CT, photon-counting detector CT; UHR, ultra-high-resolution.

## Data Availability

No new patient data were created or analyzed in this study. The charted dataset for all 236 charted records is provided as Table S1. All cited records are identifiable by the PubMed identifiers and digital object identifiers given in the reference list. Search strings supporting the findings of Section 2 and Section 3.7 are provided in File S1.

## Supplementary Materials

The following supporting information can be downloaded at: https://www.mdpi.com/article/10.3390/diagnostics16213703/s1, Supplementary File S1, Verbatim search strategy and record accounting, the verbatim query strings for all 97 searches, the raw returned records and the triage script, permitting the search of 24 July 2026 to be re-executed and its returned records verified, with an addendum giving the trial-registry search (Section 2.2) and the protocol, decisions and list of eligible records for the re-screen of records not selected, as well as a summary of the protocol, with its deviations, for the screen of the confirmatory-search records (Section 2.3); Supplementary File S2, PRISMA-ScR Checklist, the completed PRISMA-ScR checklist, giving the location in the manuscript of each reported item; Supplementary File S3, Sample-size calculations, the sample-size calculations in Section 5.3 and Section 9.2, with their assumptions; Supplementary File S4, Figure 1: Scoring rule and cell-by-cell justification, the scoring rule and cell-by-cell justification for Figure 1, with a sensitivity analysis at alternative participant thresholds, and the key from the study labels in Figure 1, Figure 2, Figure 3, Figure 4, Figure 5, Figure 6, Figure 7, Figure 8, Figure 9 and Figure 10 to reference numbers (Table S4-K); Supplementary File S5, Extended platform, regulatory and economic detail, the detailed platform-by-platform narrative (Section 3.2, Section 3.3, Section 3.4, Section 3.5, Section 3.6 and Section 3.7), regulatory discussion (Section 6) and economic detail (Section 7.2) summarized in the main text; Supplementary File S6, Sensitivity analysis of the evidence map, the comparison of Figure 1, Table 3 and the platform summaries between the 121 records charted at submission, the merged dataset and the dataset with the record charted from its full text and the 307 records mapped from abstracts (Section 8); Supplementary File S7, Screening of the confirmatory-search records, every record identified by the confirmatory search with both AI passes’ decisions, the final decision, the rule applied and a one-line reason, the exclusions by reason and the Figure 1 fields mapped from the abstracts of the 307 records mapped from abstracts only, with source quotes (Section 2.3 and Section 8); Table S1, Charted dataset for all 236 charted records (PRISMA-ScR item 15), with the clinical population of each record and the full-text confirmation status of the 57 charted studies supporting Figure 1 and Table 1, Table 2 and Table 3 (the 58th, [79], is in File S7).

## Abbreviations

Trial and cohort acronyms are proper names, are defined at first mention in the text, and are not repeated here.

Abbreviation	Definition
^18^F-NaF	fluorine-18 sodium fluoride
AI	artificial intelligence
AI-QCPA	AI-enabled quantitative coronary plaque analysis
AI-QCT	atherosclerosis imaging–quantitative computed tomography (Cleerly platform)
AUC	area under the receiver operating characteristic curve
BMI	body mass index
CABG	coronary artery bypass grafting
CAC	coronary artery calcium
CAD	coronary artery disease
CAD-RADS	Coronary Artery Disease Reporting and Data System
CCTA	coronary computed tomography angiography
CI	confidence interval
CLAIM	Checklist for Artificial Intelligence in Medical Imaging
CMR	cardiovascular magnetic resonance
CONSORT-AI	Consolidated Standards of Reporting Trials—Artificial Intelligence extension
CT	computed tomography
CTA	computed tomography angiography
DECT	dual-energy computed tomography
EID-CT	energy-integrating detector computed tomography
FAI	fat attenuation index
FDA	US Food and Drug Administration
FFR	fractional flow reserve
FFR-CT	computed tomography–derived fractional flow reserve
HR	hazard ratio
HTA	health technology assessment
ICA	invasive coronary angiography
ICC	intraclass correlation coefficient
ICER	incremental cost-effectiveness ratio
IVUS	intravascular ultrasound
LAP	low-attenuation plaque
LDL-C	low-density lipoprotein cholesterol
LRNC	lipid-rich necrotic core
MACE	major adverse cardiovascular event
METRICS	METhodological RadiomICs Score
MI	myocardial infarction
ML	machine learning
MRI	magnetic resonance imaging
NHLBI	National Heart, Lung, and Blood Institute
NIHR	National Institute for Health and Care Research
NNT	number needed to treat
NRI	net reclassification index
OCT	optical coherence tomography
PAV	percentage atheroma volume
PCAT	pericoronary adipose tissue
PCCP	Predetermined Change Control Plan
PCD-CT	photon-counting detector computed tomography
PCI	percutaneous coronary intervention
PET	positron emission tomography
PPV	positive predictive value
PRIME	Proposed Requirements for Cardiovascular Imaging-Related Machine Learning Evaluation
PRISMA	Preferred Reporting Items for Systematic Reviews and Meta-Analyses
PRISMA-ScR	Preferred Reporting Items for Systematic Reviews and Meta-Analyses extension for Scoping Reviews
PROBAST	Prediction model Risk Of Bias ASsessment Tool
QALY	quality-adjusted life year
QCA	quantitative coronary angiography
QCI	Quantitative Cardiovascular Imaging
QIBA	Quantitative Imaging Biomarkers Alliance
RQS	Radiomics Quality Score
RR	relative risk
SCAI	Society for Cardiovascular Angiography and Interventions
SCCT	Society of Cardiovascular Computed Tomography
SPECT	single-photon emission computed tomography
SPIRIT-AI	Standard Protocol Items: Recommendations for Interventional Trials—Artificial Intelligence extension
TAVR	transcatheter aortic valve replacement
UHR	ultra-high-resolution
VMI	virtual monoenergetic image
