# Supplementary material for: Reproducibility Before Treatment Decision Rules: A Targeted Scoping Review of AI-Assisted Coronary Plaque Characterization
Source: Diagnostics (Basel). 2026 Nov 9;16(21):3703. doi: 10.3390/diagnostics16213703 (PMC13762246; doi:10.3390/diagnostics16213703)
Supplement: Supplementary file 1 [file diagnostics-16-03703-s001.zip › FileS1_addendum/registry_search/ictrp_raw/ictrp_I2_p1.html]

ICTRP Search Portal


Home

Advanced Search

|  |  |
| --- | --- |
| List By |  |

Search Tips

|  |  |
| --- | --- |
| UTN |  |

|  |  |
| --- | --- |
| ICTRP website |  |

Contact us

List By Health Topics

List By Countries

List By Regions/Countries

UTN request

UTN validation

In English

In French

In Spanish

In Arabic

In Chinese

In Russian

|  |  |  |
| --- | --- | --- |
|  |  | Terms and Conditions for Use of WHO ICTRP Data  1. Introduction  The following Terms and Conditions apply for use of the WHO ICTRP data. Downloading the WHO ICTRP data indicates your acceptance of the following Terms and Conditions. These Terms and Conditions apply to all data obtained from the WHO ICTRP , independent of format and method of acquisition.  2. WHO ICTRP Database  The World Health Organization, through its International Clinical Trials Registry Platform, has developed the ICTRP database to provide patients, family members and members of the public current information about clinical research studies. The ICTRP database contains information about clinical trials being conducted throughout the world. This data is provided to the ICTRP by registries conforming to WHO standards. ICTRP is updated weekly. Trial data posted on this search portal are not endorsed by WHO, but are provided as a service to our users. In no event shall the World Health Organization be liable for any damages arising from the use of the data in the ICTRP database. None of the information obtained through use of the search portal should in any way be used in clinical care without consulting a physician or licensed health professional. WHO is not responsible for the accuracy, completeness and/or use made of the content displayed for any trial record.  3. Availability  ICTRP data are publicly available to all requesters, at no charge.  4. Use of ICTRP Data  a. Neither the WHO ICTRP, nor any of its data providers, make any warranties, expressed or implied, with respect to data contained in the database, and, furthermore, assume no liability for any party's use, or the results of such use, of any part of the database.  b. In any publication or distribution of these data (1) you should, to the extent possible and appropriate, attribute the source of the data as WHO ICTRP; (2) you should update the data such that they are current at all times; (3) you should clearly display the date the data were processed by WHO ICTRP;  c. You shall not assert any proprietary rights to any portion of the ICTRP database.  d. You shall not use information extracted from the ICTRP database for marketing, promotional or commercial purposes.  e. You shall not use the name or emblem of WHO in association with the use of data from the ICTRP database.  These terms and conditions are in effect as long as the user retains any of the data obtained from the WHO ICTRP database . |

37 records for 34 trials found for: plaque AND CCTA
(What is this?)

Trials are sometimes recorded in more than one registry.
These records can refer to each other using the 'Secondary ID' field.
The search portal uses these Secondary IDs to group records about the same trial together in the search results.
  
Each group of records referring to a trial is displayed in table that is seen by pressing the + symbol.
The record with the earliest date of registration is always shown first.

Show 
10
20
50
100
records per page

Display synonyms

|  |  |  |  |  |  |  |
| --- | --- | --- | --- | --- | --- | --- |
| 1 \| 2 \| 3 \| 4 | | | | | | |
| Recruitment status | Prospective Registration | Main ID |  | Public Title | Date of Registration | Results available |
| Recruiting |  | NCT07447648 |  | Assessing the Impact of Intensification of Lipid Lowering Therapy With Guidelines-based Evinacumab Administration on Coronary Plaque Volumes Measured by Coronary Computed Tomography Angiography (CCTA) in Patients With Homozygous Familial Hypercholesterolemia (HoFH) | 2026-02-19 |  |
| Recruiting |  | NCT07293260 |  | Olpasiran Trials of Cardiovascular Events and Lipoprotein(a) Reduction - Coronary Computed Tomography Angiography Trial | 2025-12-17 |  |
| Recruiting |  | DRKS00038652 |  | Prospective comparison of Coronary Computed-Tomography-Angiography (CCTA) with Near-Infrared Spectroscopy-Intravascular Ultrasound (NIRS-IVUS) in patients with angina. | 2025-12-05 |  |
| Recruiting |  | CTIS2025-521117-88-00 |  | OCEAN(a)-CCTA - Olpasiran trials of Cardiovascular Events And LipoproteiN(a) reduction -Coronary Computed Tomography Angiography trial | 2025-08-28 |  |
| Not recruiting |  | NCT07117084 |  | Imaging Markers of High-Risk Plaque Phenotype for Predicting Post-PCI Outcomes | 2025-08-05 |  |
| Not Recruiting |  | ChiCTR2400093460 |  | Detection of INFLAMed and vulnerable coronary plaque using artificial intelligence Modality in Computed Tomography angiography | 2024-12-05 |  |
| Not Recruiting |  | CTIS2024-511126-31-00 |  | Coronary Computed Tomography Study to Assess the Effect of Inclisiran in Addition to Maximally Tolerated Statin Therapy on Atherosclerotic Plaque Progression in Participants With a Diagnosis of Non-obstructive Coronary Artery Disease Without Previous Cardiovascular Events (V-PLAQUE) | 2024-06-21 |  |
| Not recruiting |  | NCT06376851 |  | AI DErived Plaque Quantification: CCTA and AI-QCPA for Determining Effective CAD Management | 2024-03-28 |  |
| Not recruiting |  | NCT06305559 |  | A CCTA Imaging Trial to Evaluate the Effect of Obicetrapib/Ezetimibe on Coronary Plaque Recruitment status \| Main ID \| Public title \| Date of registration \| Results available; Recruiting \| CTIS2023-508475-36-00 \| A trial to test the effect of obicetrapib/ezetimibe on coronary plaque characteristics on coronary computerized tomography angiography in people with atherosclerotic cardiovascular disease \| 28/03/2024 \| | 2024-02-28 |  |
| Recruiting |  | ChiCTR2400079760 |  | Clinical study on evaluation of coronary atherosclerotic plaque in Type 2 Diabetes Mellitus based on CCTA | 2024-01-11 |  |
|  |  |  |  |  |  |  |
| 1 \| 2 \| 3 \| 4 | | | | | | |

Disclaimer: Trials posted on this search portal are not endorsed by WHO, but are provided as a service to our users. In no event shall the World Health Organization be liable for any damages arising from the use of the information linked to in this section. None of the information obtained through use of the search portal should in any way be used in clinical care without consulting a physician or licensed health professional. WHO is not responsible for the accuracy, completeness and/or use made of the content displayed for any trial record.

Copyright - World Health Organization
- Version 3.6 -
Version history
