# Supplementary material for: Reproducibility Before Treatment Decision Rules: A Targeted Scoping Review of AI-Assisted Coronary Plaque Characterization
Source: Diagnostics (Basel). 2026 Nov 9;16(21):3703. doi: 10.3390/diagnostics16213703 (PMC13762246; doi:10.3390/diagnostics16213703)
Supplement: Supplementary file 1 [file diagnostics-16-03703-s001.zip › FileS1_addendum/registry_search/ictrp_raw/records/ACTRN12626000135314.html]

ICTRP Search Portal


Home

Advanced Search

|  |  |
| --- | --- |
| List By |  |

Search Tips

|  |  |
| --- | --- |
| UTN |  |

|  |  |
| --- | --- |
| ICTRP website |  |

Contact us

List By Health Topics

List By Countries

List By Regions/Countries

UTN request

UTN validation

In English

In French

In Spanish

In Arabic

In Chinese

In Russian

Main

Note: This record shows only 24 elements of the WHO Trial Registration Data Set. To view changes that have been made to the source record, or for additional information about this trial, click on the URL below to go to the source record in the primary register.

|  |  |
| --- | --- |
| Register: | ANZCTR |
| Last refreshed on: | 9 February 2026 |
| Main ID: | ACTRN12626000135314 |
| Date of registration: | 03/02/2026 |
| Prospective Registration: | Yes |
| Primary sponsor: | Victorian Heart Institute - Monash University |
| Public title: | IMPACT-AI: IMaging to Personalise coronary Artery disease management using Computed Tomography and Artificial Intelligence in Adults referred for CT coronary angiography |
| Scientific title: | IMPACT-AI: IMaging to Personalise coronary Artery disease management using Computed Tomography and Artificial Intelligence in Adults referred for clinically indicated CT coronary angiography |
| Date of first enrolment: | 23/02/2026 |
| Target sample size: | 480 |
| Recruitment status: | Not yet recruiting |
| URL: | https://anzctr.org.au/ACTRN12626000135314.aspx |
| Study type: | Interventional |
| Study design: | Purpose: Treatment; Allocation: Randomised controlled trial; Masking: Blinded (masking used);Assignment: Parallel;Type of endpoint: Efficacy; |
| Phase: | Not Applicable |

|  |  |  |  |  |  |  |  |
| --- | --- | --- | --- | --- | --- | --- | --- |
| Countries of recruitment | | | | | | | |
| Australia |  |  |  |  |  |  |  |

Contacts

|  |  |
| --- | --- |
| Contact type: | Public Queries |
| Name: | Dr Sarang Paleri |
| Address: | Victorian Heart Hospital, 631 Blackburn Road, Clayton 3168   Australia |
| Telephone: | +61 3 7511 1111 |
| Email: | sarang.paleri@monash.edu |
| Affiliation: |  |

|  |  |
| --- | --- |
| Contact type: | Scientific Queries |
| Name: | Dr Sarang Paleri |
| Address: | Victorian Heart Hospital, 631 Blackburn Road, Clayton 3168   Australia |
| Telephone: | +61 3 7511 1111 |
| Email: | sarang.paleri@monash.edu |
| Affiliation: |  |

Key inclusion & exclusion criteria

Inclusion criteria: Adults referred for clinically indicated CT coronary angiography at the Victorian Heart Hospital.
  
Presence of at least one coronary plaque on CTCA suitable for quantitative analysis (i.e. non-normal scan).
  
Adequate CTCA image quality to allow AI-based quantitative plaque assessment.
  
No left main coronary artery stenosis greater than or equal to 50% on CTCA.
  
Ability to provide written informed consent.

Health Condition(s) or Problem(s) studied

Cardiovascular - Coronary heart disease

Coronary artery disease;   
Coronary artery disease

Intervention(s)

The intervention is a personalised model of care guided by an artificial intelligence (AI) enabled, computed tomography coronary angiography (CTCA)-based clinical decision support tool (AI-CTCA), compared with usual care based on standard visual CTCA interpretation alone.  
  
In the intervention arm, all participants undergo clinically indicated CTCA on a 320-detector scanner using a standardised acquisition protocol; images are then analysed with FDA-cleared deep learning software (Autoplaque v3.0) that automatically segments the coronary arteries and quantifies total, calcified, and non-calcified plaque volumes and stenosis severity for all coronary segments greater or equal to 1.5 mm. The software also generates an Ischaemia Risk Score (0–100) per vessel, integrates plaque volume into age- and sex-specific percentile categories, and produces a one-page AI-CTCA report with recommendations for downstream functional testing or invasive coronary angiography and for medical therapy based on a predefined AI-CTCA Treatment Algorithm.  
  
Reporting clinicians in the intervention arm first perform standard visual CTCA interpretation (Society of Cardiovascular Computed Tomography 18-segment model), then review the AI-CTCA report and repeat their assessment, documenting plaque burden, and confidence scores in electronic questionnaires. Reporting clinicians will undergo an individualised training session for 1 hour with the study coordinators and provided a Clinician Information Sheet prior to commencing analysis for the trial. Subsequent care for these patients is transferred, with the referrer’s permission, to a cardiologist-led team trained in AI-CTCA interpretation, who use the AI-CTCA recommendations and Ischaemia Risk Score to guide referral for functional non-invasi

Primary Outcome(s)

Primary outcome: change in per-patient non-calcified plaque volume on CTCA at 24 months.[Change in per-patient non-calcified plaque volume will be measured on serial CTCA (baseline and 24 months) using an FDA-cleared AI-based quantitative plaque analysis software (Autoplaque v3.0), with all coronary segments greater than or equal to 1.5 mm analysed, plaque volumes derived from automated segmentation with adaptive Hounsfield unit thresholds, summed to patient-level total non-calcified plaque volume, and 24-month change quantified using blinded image analysis in a core laboratory, with mixed-effects linear regression used to compare change between groups adjusted for baseline plaque volume, CTCA interval, demographics, risk factors, medications, and LDL-C change. Baseline and 24 months post initial CTCA.]

Secondary Outcome(s)

Change in clinician-reported CAD-RADS (Coronary Artery Disease – Reporting and Data System) stenosis category[Standard visual CTCA interpretation using SCCT 18-segment model and CAD-RADS in all participants.
  
In the intervention arm only, repeat CTCA interpretation after access to the AI-CTCA quantitative plaque report, with electronic questionnaire capturing CAD-RADS category before and after AI-CTCA report unblinding. At baseline (index CTCA), immediately at point-of-care reporting before and after AI-CTCA report in the intervention arm.]

Change in plaque burden category[Standard visual CTCA interpretation using SCCT 18-segment model in all participants.
  
In the intervention arm only, repeat CTCA interpretation after access to the AI-CTCA quantitative plaque report, with an electronic questionnaire capturing plaque burden category. At baseline (index CTCA), immediately at point-of-care reporting before and after AI-CTCA report in the intervention arm.]

Proportion of patients undergoing downstream functional non-invasive imaging and invasive coronary angiography (ICA).[Prospectively collected data on all cardiac investigations including functional non-invasive imaging (stress echo, nuclear stress testing, stress CMR) and ICA from electronic medical records and participant report.
  
Standardised follow-up questionnaires capturing non-invasive tests and ICA over the preceding 6 months. Routine clinical data collection after the index CTCA.
  
Structured follow-up at 6, 12, 18, and 24 months via phone/SMS/email questionnaires.]

Change in clinician confidence scores at point-of-care (pre- vs post-AI-CTCA report in the intervention arm).[In the intervention arm only, repeat CTCA interpretation after access to the AI-CTCA quantitative plaque report, with an electronic questionnaire capturing confidence on a 5-point Likert scale before and after AI-CTCA report unblinding. At baseline (index CTCA), immediately at point-of-care reporting before and after AI-CTCA report in the intervention arm.]

Proportion of patients undergoing coronary revascularisation (PCI or CABG).[Documentation of PCI and CABG procedures from hospital electronic medical records and through the standardised follow-up questionnaire.
  
Medicare/PBS data linkage where available to corroborate procedure data. From randomisation until 24 months.
  
Captured at 6, 12, 18, and 24 months follow-up contacts.]

Secondary ID(s)

Nil known

Source(s) of Monetary Support

NHMRC

Secondary Sponsor(s)

Ethics review

|  |  |
| --- | --- |
| Status: | Not approved |
| Approval date: |  |
| Contact name: |  |
| Contact address: |  |
| Contact telephone: |  |
| Contact email: |  |

Results

|  |  |
| --- | --- |
| Results available: |  |
| URL: |  |
| URL of the protocol: |  |
| Date Posted: |  |
| Date of completion: |  |
| Date of first publication: |  |
| Results summary: |  |
| Baseline characteristics: |  |
| Adverse events: |  |
| Outcome measures: |  |
| IPD sharing plan: |  |
| IPD sharing description: |  |

Disclaimer: Trials posted on this search portal are not endorsed by WHO, but are provided as a service to our users. In no event shall the World Health Organization be liable for any damages arising from the use of the information linked to in this section. None of the information obtained through use of the search portal should in any way be used in clinical care without consulting a physician or licensed health professional. WHO is not responsible for the accuracy, completeness and/or use made of the content displayed for any trial record.

Copyright - World Health Organization
- Version 3.6 -
Version history
