# Supplementary material for: Reproducibility Before Treatment Decision Rules: A Targeted Scoping Review of AI-Assisted Coronary Plaque Characterization
Source: Diagnostics (Basel). 2026 Nov 9;16(21):3703. doi: 10.3390/diagnostics16213703 (PMC13762246; doi:10.3390/diagnostics16213703)
Supplement: Supplementary file 1 [file diagnostics-16-03703-s001.zip › FileS1_addendum/registry_search/ictrp_raw/records/ACTRN12626000150347.html]

ICTRP Search Portal


Home

Advanced Search

|  |  |
| --- | --- |
| List By |  |

Search Tips

|  |  |
| --- | --- |
| UTN |  |

|  |  |
| --- | --- |
| ICTRP website |  |

Contact us

List By Health Topics

List By Countries

List By Regions/Countries

UTN request

UTN validation

In English

In French

In Spanish

In Arabic

In Chinese

In Russian

Main

Note: This record shows only 24 elements of the WHO Trial Registration Data Set. To view changes that have been made to the source record, or for additional information about this trial, click on the URL below to go to the source record in the primary register.

|  |  |
| --- | --- |
| Register: | ANZCTR |
| Last refreshed on: | 16 February 2026 |
| Main ID: | ACTRN12626000150347 |
| Date of registration: | 06/02/2026 |
| Prospective Registration: | Yes |
| Primary sponsor: | Asanka Wijetunga, Westmead Hospital Department of Vascular Surgery |
| Public title: | Computed Tomography Coronary Angiography Screening of Asymptomatic Patients With Chronic Limb-Threatening Ischaemia at Hospital Discharge: A Randomised Control Trial |
| Scientific title: | Computed Tomography Coronary Angiography Screening of Asymptomatic Patients With Chronic Limb-Threatening Ischaemia at Hospital Discharge: A Randomised Control Trial |
| Date of first enrolment: | 01/07/2026 |
| Target sample size: | 400 |
| Recruitment status: | Not yet recruiting |
| URL: | https://anzctr.org.au/ACTRN12626000150347.aspx |
| Study type: | Interventional |
| Study design: | Purpose: Prevention; Allocation: Randomised controlled trial; Masking: Blinded (masking used); |
| Phase: | Not Applicable |

|  |  |  |  |  |  |  |  |
| --- | --- | --- | --- | --- | --- | --- | --- |
| Countries of recruitment | | | | | | | |
| Australia |  |  |  |  |  |  |  |

Contacts

|  |  |
| --- | --- |
| Contact type: | Public Queries |
| Name: | Asanka Wijetunga |
| Address: | Westmead Hospital. Cnr Darcy and Hawkesbury Roads, Westmead, NSW, 2145   Australia |
| Telephone: | +61288905555 |
| Email: | asanka.wijetunga@health.nsw.gov.au |
| Affiliation: |  |

|  |  |
| --- | --- |
| Contact type: | Scientific Queries |
| Name: | Asanka Wijetunga |
| Address: | Westmead Hospital. Cnr Darcy and Hawkesbury Roads, Westmead, NSW, 2145   Australia |
| Telephone: | +61288905555 |
| Email: | asanka.wijetunga@health.nsw.gov.au |
| Affiliation: |  |

Key inclusion & exclusion criteria

Inclusion criteria: Age greater than or equal to 18 years.
  
Index admission under vascular surgery for CLTI according to current Global Vascular Guidelines (https://pmc.ncbi.nlm.nih.gov/articles/PMC8365864/#F4 - Accessed 19:09 31/12/2025)
  
  
Objectively documented atherosclerotic PAD
  
- Clinical history and examination
  
- Ankle-brachial pressure index (ABPI) / Ankle Pressure (AP) ± toe-brachial index (TBI) / Toe Presure (TP)
  
- Non-invasive vascular imaging modalities where the above are inconclusive / unavailable / where revascularisation is considered (duplex ultrasonography, computed tomography angiography / magnetic resonance angiography).
  
  
Symptoms of
  
- ischaemic rest pain
  
- tissue loss (ulceration or gangrene)
  
  
No known history of coronary artery disease (CAD):
  
- No prior myocardial infarction
  
- No prior coronary revascularisation
  
- No prior angiographically documented >50% coronary stenosis.
  
- No prior positive functional test or CT coronary angiography (CTCA) labelled as CAD.
  
  
Asymptomatic for CAD:
  
- No typical angina or anginal equivalent symptoms in the preceding 3 months.
  
  
Able to provide written informed consent.

Health Condition(s) or Problem(s) studied

Cardiovascular Disease;Peripheral Artery Disease ;Chronic Limb Threatening Ischaemia;   
Cardiovascular Disease   
Peripheral Artery Disease   
Chronic Limb Threatening Ischaemia

Cardiovascular - Coronary heart disease

Intervention(s)

Computed Tomography Coronary Angiography + Best Medical Therapy  
  
In both arms, each patient will receive an information leaflet which will be created and proofread by the panel of investigators.   
  
Arm 1: CTCA Screening + Best Medical Therapy   
CTCA   
• Performed within 90 days of hospital discharge, ideally within 30 days.  
• Scanner specifications: 64-slice multi-detector CT with detector element width 0.625mm with ECG-gating, following SCCT performance guidelines.  
• Patient preparation:  
o Rate control with oral beta-blockers targeting heart rate 100kg in accordance with 'As Low as Reasonably Achievable' (ALARA) principles of radiation safety   
o High-iodine contrast media of 270-400mg iodine/mL as per department protocol   
o Standard biphasic contrast injection protocols (50-80mL contrast media at 5-7mL/s followed by 40-50mL 0.9% sodium chloride chaser at 5-7mL/s.   
o Scan performed during inspiration with medium-sized breaths   
o Reconstructions in standard cardiac planes.  
o Estimated scan duration of 10-15 minutes however preparation including premedication, cannulation and collection of vital signs may take 30-60 mins.   
o Reporting using a structured system (e.g. Coronary Artery Disease – Reporting and Data System: CAD-RADS) including segmental stenosis severity (0–5), plaque characteristics and calcium scoring   
• FFR-CT:  
o CTCA datasets may be sent for FFR-CT analysis.  
o Ischaemia-producing lesions defined as FFR-CT <0.80, and severe ischaemia as FFR-CT <0.75   
o Absence of FFR-CT will not constitute a protocol deviation.   
Post-CTCA Management  
• Lesion management will remain at the discretion of the on-call cardiologist   
• Recorded therapeutic modifications will include   
o Initiation or intensific

Primary Outcome(s)

Time from randomisation to first occurrence of symptomatic non-fatal MI or CAD death within 12 months - they will be assessed as a composite outcome. [Fourth Universal Definition of Myocardial Infarction (https://www.ahajournals.org/doi/10.1161/CIR.0000000000000617 - 31/12/2025 18:50 AEDT)   
  
Review of hospital medical records, and history from patients at clinic visits. Within 12 months of randomisation. Clinic follow up will be organised at 1 month, 3 months, 6 months and 1 year. ]

Secondary Outcome(s)

12-month incidence of all-cause mortality or MACE - will be assessed as a composite outcome. [MACE-4: cardiovascular death, non-fatal MI, non-fatal stroke, unplanned coronary revascularisation -
  
Review of hospital medical records, and history from patients at clinic visits. Within 12 months of randomisation. Clinic follow up will be organised at 1 month, 3 months, 6 months and 1 year. ]

Major adverse limb events (MALE): above-ankle amputation or major index-limb open or endovascular re-intervention at 12 months.[According to the BEST-CLI trial (https://www.nejm.org/doi/full/10.1056/NEJMoa2207899 - 31/12/2025 18:50 AEDT)
  
  
Review of hospital medical records, and history from patients at clinic visits. Within 12 months of randomisation. Clinic follow up will be organised at 1 month, 3 months, 6 months and 1 year. ]

Secondary ID(s)

None

Source(s) of Monetary Support

Secondary Sponsor(s)

Ethics review

|  |  |
| --- | --- |
| Status: | Not approved |
| Approval date: |  |
| Contact name: |  |
| Contact address: |  |
| Contact telephone: |  |
| Contact email: |  |

Results

|  |  |
| --- | --- |
| Results available: |  |
| URL: |  |
| URL of the protocol: |  |
| Date Posted: |  |
| Date of completion: |  |
| Date of first publication: |  |
| Results summary: |  |
| Baseline characteristics: |  |
| Adverse events: |  |
| Outcome measures: |  |
| IPD sharing plan: |  |
| IPD sharing description: |  |

Disclaimer: Trials posted on this search portal are not endorsed by WHO, but are provided as a service to our users. In no event shall the World Health Organization be liable for any damages arising from the use of the information linked to in this section. None of the information obtained through use of the search portal should in any way be used in clinical care without consulting a physician or licensed health professional. WHO is not responsible for the accuracy, completeness and/or use made of the content displayed for any trial record.

Copyright - World Health Organization
- Version 3.6 -
Version history
