# Supplementary material for: Reproducibility Before Treatment Decision Rules: A Targeted Scoping Review of AI-Assisted Coronary Plaque Characterization
Source: Diagnostics (Basel). 2026 Nov 9;16(21):3703. doi: 10.3390/diagnostics16213703 (PMC13762246; doi:10.3390/diagnostics16213703)
Supplement: Supplementary file 1 [file diagnostics-16-03703-s001.zip › FileS1_addendum/registry_search/ictrp_raw/records/ACTRN12626000779370.html]

ICTRP Search Portal


Home

Advanced Search

|  |  |
| --- | --- |
| List By |  |

Search Tips

|  |  |
| --- | --- |
| UTN |  |

|  |  |
| --- | --- |
| ICTRP website |  |

Contact us

List By Health Topics

List By Countries

List By Regions/Countries

UTN request

UTN validation

In English

In French

In Spanish

In Arabic

In Chinese

In Russian

Main

Note: This record shows only 24 elements of the WHO Trial Registration Data Set. To view changes that have been made to the source record, or for additional information about this trial, click on the URL below to go to the source record in the primary register.

|  |  |
| --- | --- |
| Register: | ANZCTR |
| Last refreshed on: | 13 July 2026 |
| Main ID: | ACTRN12626000779370 |
| Date of registration: | 30/06/2026 |
| Prospective Registration: | Yes |
| Primary sponsor: | Manawaora Integrated Health and Research |
| Public title: | He Aronga Hou: Reimagining Early Detection of Heart Risk for Maori Adults |
| Scientific title: | Early Detection of Subclinical Cardiovascular Disease in Maori Adults Using Computed Tomography Coronary Angiography and N-Terminal Pro B-Type Natriuretic Peptide Testing: A Community-Led Feasibility Study |
| Date of first enrolment: | 20/07/2026 |
| Target sample size: | 100 |
| Recruitment status: | Not yet recruiting |
| URL: | https://anzctr.org.au/ACTRN12626000779370.aspx |
| Study type: | Interventional |
| Study design: | Purpose: Prevention; Allocation: Non-randomised trial; Masking: Open (masking not used);Assignment: Single group; |
| Phase: | Not Applicable |

|  |  |  |  |  |  |  |  |
| --- | --- | --- | --- | --- | --- | --- | --- |
| Countries of recruitment | | | | | | | |
| New Zealand |  |  |  |  |  |  |  |

Contacts

|  |  |
| --- | --- |
| Contact type: | Public Queries |
| Name: | Tasha Burton |
| Address: | Manawaora integrated Health and Research, 103 Third Avenue, 3110, Tauranga   New Zealand |
| Telephone: | +64 220180557 |
| Email: | tasha@thecentreforhealth.co.nz |
| Affiliation: |  |

|  |  |
| --- | --- |
| Contact type: | Scientific Queries |
| Name: | Tasha Burton |
| Address: | Manawaora integrated Health and Research, 103 Third Avenue, 3110, Tauranga   New Zealand |
| Telephone: | +64 220180557 |
| Email: | tasha@thecentreforhealth.co.nz |
| Affiliation: |  |

Key inclusion & exclusion criteria

Inclusion criteria: Inclusion Criteria:
  
• Maori men aged 30–59 years
  
• Maori women aged 40–59 years
  
• Classified as intermediate risk (5–14% five-year CVD risk) using the PREDICT tool
  
• Able to provide informed consent

Health Condition(s) or Problem(s) studied

Cardiovascular - Coronary heart disease

Cardiovascular Health ;   
Cardiovascular Health

Intervention(s)

What is the intervention  
The intervention is a community-based cardiovascular screening programme designed to support the early detection of subclinical cardiovascular disease in Maori adults at intermediate predicted cardiovascular risk. Participants undergo computed tomography coronary angiography (CTCA) with coronary artery calcium scoring and plaque assessment, blood biomarker testing including N-terminal pro B-type natriuretic peptide (NT-proBNP), and cardiovascular risk assessment. Participants identified as higher risk are referred back to their primary care provider with cardiology-supported recommendations for further investigation and management.  
  
How participants will gain access to the intervention  
Participants will primarily be identified and referred through partnering Maori health providers and affiliated primary care/general practice services (e.g., Ngati Whatua Orakei, Ngati Porou Oranga, Turanga Health). Recruitment pathways may include:  
  
• Referral from primary healthcare providers or nurse-led clinics  
• Identification through existing cardiovascular risk assessment programmes  
• Community outreach and provider-led engagement  
• Self-expression of interest following community promotion approved by partner organisations  
  
Once referred, participants will be contacted by the research/provider team to confirm eligibility and arrange screening appointments.  
  
  
Who delivers it and their relevant expertise  
The study is delivered collaboratively by Maori health providers, the research team, radiology services, and cardiology specialists. Maori health provider research staff and coordinators support recruitment, consent, participant engagement, and study logistics within their communities. CTCA imaging is performed by

Primary Outcome(s)

Feasibility will be assessed as a composite outcome using:  
(1) recruitment rate (proportion of eligible individuals who provide informed consent);  
(2) completion rate of coronary CT angiography (CTCA) and staged investigations including NT-proBNP testing and echocardiography where clinically indicated;  
(3) proportion of participants successfully completing the full assessment pathway;  
(4) participant retention and follow-up completion rates[Feasibility will be assessed using: (1) recruitment rate (proportion of eligible individuals who provide informed consent) from study recruitment record; (2) completion rate of coronary CT angiography (CTCA) and staged investigations including NT-proBNP testing and echocardiography where clinically indicated assessed from study data base; (3) proportion of participants successfully completing the full assessment pathway assessed from study records and study completion logs; (4) participant retention and follow-up completion rates calculated as the proportion of enrolled participants who complete the scheduled follow-up assessments.  
  
 End of study participation (following completion of all cardiovascular assessments and follow-up procedures, approximately 3 months after enrolment).]

Detection of subclinical cardiovascular disease through the enhanced cardiovascular assessment pathway.[Assessed by the proportion of participants with evidence of coronary artery plaque on CTCA, distribution of coronary artery calcium scores, proportion of participants with structural heart disease where echocardiography is undertaken, and proportion of participants reclassified from intermediate to higher cardiovascular risk following enhanced assessment.  
  
Coronary artery calcium score  
Presence and extent of coronary plaque  
Coronary artery stenosis severity At completion of the enhanced cardiovascular assessment pathway, following CTCA and any indicated staged testing.]

Secondary Outcome(s)

N-terminal pro B-type natriuretic peptide (NT-proBNP) concentrations[Venous blood sample analysed by accredited pathology laboratory Assessed at screening visit]

Changes to cardiovascular risk classification following computed tomography coronary angiography (CTCA) findings[Comparison of baseline predicted cardiovascular risk with post-CTCA risk assessment and imaging findings Following CTCA reporting]

Identification of previously undiagnosed cardiovascular abnormalities or incidental findings[This will be assessed as a composite outcome. CTCA imaging report, echocardiography findings (if performed), and clinician review. Following completion of imaging investigations]

Proportion of participants referred for echocardiography following elevated NT-proBNP results[Review of NT-proBNP results and clinical referral records Following baseline blood test results]

Secondary ID(s)

nil

Source(s) of Monetary Support

Putahi Manawa | Healthy Hearts Aotearoa New Zealand

Secondary Sponsor(s)

Ethics review

|  |  |
| --- | --- |
| Status: | Approved |
| Approval date: | 11/05/2026 |
| Contact name: |  |
| Contact address: |  |
| Contact telephone: |  |
| Contact email: |  |

Results

|  |  |
| --- | --- |
| Results available: |  |
| URL: |  |
| URL of the protocol: |  |
| Date Posted: |  |
| Date of completion: |  |
| Date of first publication: |  |
| Results summary: |  |
| Baseline characteristics: |  |
| Adverse events: |  |
| Outcome measures: |  |
| IPD sharing plan: |  |
| IPD sharing description: |  |

Disclaimer: Trials posted on this search portal are not endorsed by WHO, but are provided as a service to our users. In no event shall the World Health Organization be liable for any damages arising from the use of the information linked to in this section. None of the information obtained through use of the search portal should in any way be used in clinical care without consulting a physician or licensed health professional. WHO is not responsible for the accuracy, completeness and/or use made of the content displayed for any trial record.

Copyright - World Health Organization
- Version 3.6 -
Version history
