# Supplementary material for: Reproducibility Before Treatment Decision Rules: A Targeted Scoping Review of AI-Assisted Coronary Plaque Characterization
Source: Diagnostics (Basel). 2026 Nov 9;16(21):3703. doi: 10.3390/diagnostics16213703 (PMC13762246; doi:10.3390/diagnostics16213703)
Supplement: Supplementary file 1 [file diagnostics-16-03703-s001.zip › FileS1_addendum/registry_search/ictrp_raw/records/ChiCTR2400093460.html]

ICTRP Search Portal


Home

Advanced Search

|  |  |
| --- | --- |
| List By |  |

Search Tips

|  |  |
| --- | --- |
| UTN |  |

|  |  |
| --- | --- |
| ICTRP website |  |

Contact us

List By Health Topics

List By Countries

List By Regions/Countries

UTN request

UTN validation

In English

In French

In Spanish

In Arabic

In Chinese

In Russian

Main

Note: This record shows only 24 elements of the WHO Trial Registration Data Set. To view changes that have been made to the source record, or for additional information about this trial, click on the URL below to go to the source record in the primary register.

|  |  |
| --- | --- |
| Register: | ChiCTR |
| Last refreshed on: | 9 December 2024 |
| Main ID: | ChiCTR2400093460 |
| Date of registration: | 2024-12-05 |
| Prospective Registration: | Yes |
| Primary sponsor: | Beijing Anzhen Hospital, Capital Medical University |
| Public title: | Detection of INFLAMed and vulnerable coronary plaque using artificial intelligence Modality in Computed Tomography angiography |
| Scientific title: | Detecting coronary plaque-specific inflammation using an artificial-intelligence aided computed tomography software |
| Date of first enrolment: | 2025-01-01 |
| Target sample size: | Target condition:66;Difficult condition:0 |
| Recruitment status: | Pending |
| URL: | https://www.chictr.org.cn/showproj.html?proj=243952 |
| Study type: | Diagnostic test |
| Study design: | Diagnostic test for accuracy |
| Phase: | Diagnostic New Technique Clincal Study |

|  |  |  |  |  |  |  |  |
| --- | --- | --- | --- | --- | --- | --- | --- |
| Countries of recruitment | | | | | | | |
| China |  |  |  |  |  |  |  |

Contacts

|  |  |
| --- | --- |
| Contact type: | Public |
| Name: | Yu Du |
| Address: | Number 2, Anzhen Road, Chaoyang District, Beijing, China 100029 |
| Telephone: | +86 185 1838 9843 |
| Email: | 18518389843@163.com |
| Affiliation: | Department of Cardiology, Beijing Anzhen Hospital, Capital Medical University |

|  |  |
| --- | --- |
| Contact type: | Scientific |
| Name: | Yu Du |
| Address: | Number 2, Anzhen Road, Chaoyang District, Beijing, China 100029 |
| Telephone: | +86 185 1838 9843 |
| Email: | 18518389843@163.com |
| Affiliation: | Department of Cardiology, Beijing Anzhen Hospital, Capital Medical University |

Key inclusion & exclusion criteria

Inclusion criteria: 1.Age > 18;  
2.Signed informed consent form;  
3.Participants who diagnosed with stable angina, unstable angina or myocardial infarction (1~8 weeks from symptom onset), undergoing invasive coroanry angiography and optical coherence tomography;  
4.Non-culprit plaque with diameter stenosis 25%~75% within a coronary artery = 2.0mm in diameter based on coronary angiography.

Health Condition(s) or Problem(s) studied

Coronary Atherosclerotic Heart Disease

Intervention(s)

Gold Standard:Gold standard of plaque active inflammation based on PET-CT: 18F-NaF TBRmax > 1.30;  
Gold standard of anatomic high-risk plaque met at least two of the following criteria based on OCT: fibrous hat thickness < 75µm, maximal lipid pool curve > 180°, or detection of macrophages.;Index test:Plaque active inflammation based on CCTA-AI software (Shanghai United Imaging Healthcare Co., Ltd): plaque specific fat attenuation index (pFAI);  
Anatomic high-risk plaque met at least two of the following criteria based on CCTA-AI software (Shanghai United Imaging Healthcare Co., Ltd): positive remodeling, low-attenuation plaque, napkin-ring sign, and spotty calcification.;

Primary Outcome(s)

Plaque specific fat attenuation index (pFAI);Anatomic high-risk plaque based on CCTA;

Secondary Outcome(s)

Secondary ID(s)

Source(s) of Monetary Support

Scientific and Technological Innovation 2030 - Major Project (Youth Training Program)

Secondary Sponsor(s)

Ethics review

|  |  |
| --- | --- |
| Status: | Approved |
| Approval date: | 29/09/2024 |
| Contact name: | Chaoyang Wu |
| Contact address: | Number 2, Anzhen Road, Chaoyang District, Beijing, China |
| Contact telephone: | +86 10 6445 6214 |
| Contact email: |  |

Results

|  |  |
| --- | --- |
| Results available: |  |
| URL: |  |
| URL of the protocol: |  |
| Date Posted: |  |
| Date of completion: |  |
| Date of first publication: |  |
| Results summary: |  |
| Baseline characteristics: |  |
| Adverse events: |  |
| Outcome measures: |  |
| IPD sharing plan: | Yes |
| IPD sharing description: |  |

Disclaimer: Trials posted on this search portal are not endorsed by WHO, but are provided as a service to our users. In no event shall the World Health Organization be liable for any damages arising from the use of the information linked to in this section. None of the information obtained through use of the search portal should in any way be used in clinical care without consulting a physician or licensed health professional. WHO is not responsible for the accuracy, completeness and/or use made of the content displayed for any trial record.

Copyright - World Health Organization
- Version 3.6 -
Version history
