# Supplementary material for: Reproducibility Before Treatment Decision Rules: A Targeted Scoping Review of AI-Assisted Coronary Plaque Characterization
Source: Diagnostics (Basel). 2026 Nov 9;16(21):3703. doi: 10.3390/diagnostics16213703 (PMC13762246; doi:10.3390/diagnostics16213703)
Supplement: Supplementary file 1 [file diagnostics-16-03703-s001.zip › FileS1_addendum/registry_search/ictrp_raw/records/CTIS2025-522868-32-00.html]

ICTRP Search Portal


Home

Advanced Search

|  |  |
| --- | --- |
| List By |  |

Search Tips

|  |  |
| --- | --- |
| UTN |  |

|  |  |
| --- | --- |
| ICTRP website |  |

Contact us

List By Health Topics

List By Countries

List By Regions/Countries

UTN request

UTN validation

In English

In French

In Spanish

In Arabic

In Chinese

In Russian

Main

Note: This record shows only 24 elements of the WHO Trial Registration Data Set. To view changes that have been made to the source record, or for additional information about this trial, click on the URL below to go to the source record in the primary register.

|  |  |
| --- | --- |
| Register: | CTIS |
| Last refreshed on: | 29 September 2025 |
| Main ID: | CTIS2025-522868-32-00 |
| Date of registration: | 27/06/2025 |
| Prospective Registration: | No |
| Primary sponsor: | Semmelweis University |
| Public title: | Changes in plaque characteristics after short-term statin therapy assessed with coronary CT (INTENSE) |
| Scientific title: | Changes in plaque characteristics after short-term statin therapy assessed with coronary CT - INTENSE |
| Date of first enrolment: |  |
| Target sample size: | 140 |
| Recruitment status: | Not Recruiting |
| URL: | https://euclinicaltrials.eu/search-for-clinical-trials/?lang=en&EUCT=2025-522868-32-00 |
| Study type: | Interventional clinical trial of medicinal product |
| Study design: | Controlled: no Randomised: no Open: no Single blind: no Double blind: no Parallel group: no Cross over: no Other: no If controlled, specify comparator, Other Medicinial Product: no Placebo: no Number of treatment arms in the trial: |
| Phase: | Human pharmacology (Phase I): No Therapeutic exploratory (Phase II): Yes Therapeutic confirmatory - (Phase III): Yes Therapeutic use - (Phase IV): No |

|  |  |  |  |  |  |  |  |
| --- | --- | --- | --- | --- | --- | --- | --- |
| Countries of recruitment | | | | | | | |
| Hungary |  |  |  |  |  |  |  |

Contacts

|  |  |
| --- | --- |
| Contact type: | Public |
| Name: | Barnabas Baksa |
| Address: |  |
| Telephone: | +36308771806 |
| Email: | baksa.barnabas@semmelweis.hu |
| Affiliation: | Semmelweis University |

|  |  |
| --- | --- |
| Contact type: | Scientific |
| Name: | Barnabas Baksa |
| Address: |  |
| Telephone: | +36308771806 |
| Email: | baksa.barnabas@semmelweis.hu |
| Affiliation: | Semmelweis University |

Key inclusion & exclusion criteria

Inclusion criteria: Clinically indicated coronary CT angiography, 45-75 yo. females or min. 40-75 yo. males, Statin naive patients, There are no contraindications to CT angiography, Understanding and signing the consent form, At least one partially-calcified or non-calcified plaque, FFRCT>0.75 distal to stenosis  
Exclusion criteria: History of statin or other lipid lowering (ezetimibe) treatment, FFRCT<0.75 distal to stenosis, females below 45 yo. or males below 40 yo., Diabetes mellitus (Type I. and II.), Coronary artery stent or bypass graft, Previous myocardial infarction, Acute liver disease, Hypersensitivity to any of the excipients of the investigational medicinal product, Concomitant treatment with the sofosbuvir/velpatasvir/voxilaprevir combination., Concomitant treatment with cyclosporine., Women of childbearing potential who are not using an adequate method of contraception., Elevated alanine aminotransferase levels (>3x upper limit of normal), Elevated creatine kinase (>3x upper limit of normal), Elevated low density lipoprotein (>5 mmol/L), Pregnancy or breastfeeding, >75 yo. patients (both genders), Chronic renal failure or decreased renal function (eGFR <30 ml/m2), Active oncological treatment, =70% luminal stenosis in proximal LAD or =50% luminal stenosis in left main coronary artery  
  
Age minimum:
18  
Age maximum:
65+  
Gender:
Female: yes Male: yes

Health Condition(s) or Problem(s) studied

Coronary Artery Disease, Cardiology, Statin Therapy, Coronary Computed Tomography Angiography, Stable chest pain   
MedDRA version: 20.0Level: PTClassification code: 10058108Term: Dyslipidaemia Class: 100000004861   
MedDRA version: 27.0Level: LLTClassification code: 10090144Term: Nonobstructive coronary artery disease Class: 100000004849   
MedDRA version: 27.0Level: LLTClassification code: 10090447Term: Coronary artery atherosclerotic heart disease Class: 100000004849   
MedDRA version: 20.0Level: PTClassification code: 10011078Term: Coronary artery disease Class: 100000004849

Therapeutic area: Diseases [C] - Cardiovascular Diseases [C14]

Intervention(s)

Product Name: Tabletta placebo 0,2 g fehér, laktózmentes (4x) tartalmú kapszula, Product Code:N/A, Pharmaceutical Form: N/A, Other descriptive name: N/A , Strength: , Pharmaceutical form of the placebo: N/A , Product Name: Xeter 10 mg filmtabletta, Product Code:PRD671566, Pharmaceutical Form: FILM-COATED TABLET, Other descriptive name: , Strength:

Primary Outcome(s)

Primary end point(s): Non-calcified plaque volume at 3 months follow-up (expressed as mm3).

Main Objective: Change in non-calcified plaque volume during a 3-month follow-up period.

Secondary Objective: Change in total plaque volume at 3-month follow-up period., Change in total plaque volume during a 24-month follow-up period., Change in total plaque volume between 3- and 24-month follow-up period., Change in low attenuation non-calcified plaque volume during a 3-month follow-up period., Change in low attenuation non-calcified plaque volume during a 24-month follow-up period., Change in non-calcified plaque volume between 3- and 24-month follow-up period., Change in non-calcified plaque volume during a 24-month follow-up period., Change in plaque composition during a 3-month follow-up period., Change in plaque composition during a 24-month follow-up period., Change in per-vessel fractional flow reserve [FFRCT] during a 3-month follow-up period., Change in per-vessel fractional flow reserve [FFRCT] during a 24-month follow-up period., Change in the prevalence of high-risk plaque features during a 3-month follow-up period., Change in the prevalence of high-risk plaque features during a 24-month follow-up period., Change in radiomic features during a 24-month follow-up period., Change in radiomic features during a 3-month follow-up period., Prevalence of major adverse cardiac event, Prevalence of clinical adverse event., Changes in blood lipid profile., Changes in inflammatory markers., Changes in markers of glucose homeostasis.

Secondary Outcome(s)

Secondary end point(s):Plaque composition measured during the 3-month visit: each component expressed in mm³ – low attenuation: -100 to 30 HU; non-calcified: 30 to 350 HU; calcified: >350 HU.

Secondary end point(s):Visual presence of high risk plaque features during the 3-month follow up: low attenuation plaque, positive remodeling, napkin-ring sign, spotty calcification, minimal luminal area or plaque burden =70%

Secondary end point(s):Low-attenuation non-calcified plaque volume during the 24-month follow-up period (mm³)

Secondary end point(s):Major adverse events (death, cardiovascular death, fatal and nonfatal myocardial infarction, fatal and nonfatal stroke/TIA, unstable angina, hospitalization))

Secondary end point(s):Minor adverse events (muscle pain, constipation, abdominal pain or meteorismus, nausea, headache, vertigo, fatigue, skin rash, pruritus)

Secondary end point(s):CT-based Fractional Flow Reserve (FFR-CT) during the 3 months follow-up

Secondary end point(s):Laboratory parameters (total cholesterol, low-density lipoprotein (LDL) cholesterol, high-density lipoprotein (HDL) cholesterol, triglycerides, LP(a), apoA1, apoB, hs-CRP, ferritin and hemoglobin A1c.

Secondary end point(s):CT-based Fractional Flow Reserve (FFR-CT) during the 24 months follow-up

Secondary end point(s):Low-attenuation non-calcified plaque volume during the 3-month follow-up period (expressed in mm³)

Secondary end point(s):Total plaque volume (mm3) during the 3-month follow up

Secondary end point(s):Total plaque volume (mm3) during the 24-month follow up

Secondary end point(s):Plaque composition measured during the 24-month visit: each component expressed in mm³ – low attenuation: -100 to 30 HU; non-calcified: 30 to 350 HU; calcified: >350 HU.

Secondary end point(s):Radiomic features during the 24-month follow-up

Secondary end point(s):Radiomic features during the 3-month follow up

Secondary end point(s):Non-calcified plaque volume at 24 months follow-up (expressed as mm3).

Secondary end point(s):Visual presence of high risk plaque features during the 24-month follow up: low attenuation plaque, positive remodeling, napkin-ring sign, spotty calcification, minimal luminal area or plaque burden =70%

Secondary ID(s)

NCT06603363

Source(s) of Monetary Support

National Research, Development and Innovation Office – Hungarian Scientific Research Fund

Secondary Sponsor(s)

Ethics review

|  |  |
| --- | --- |
| Status: | Authorised |
| Approval date: | 26/09/2025 |
| Contact name: |  |
| Contact address: |  |
| Contact telephone: |  |
| Contact email: |  |

Results

|  |  |
| --- | --- |
| Results available: |  |
| URL: |  |
| URL of the protocol: | https://euclinicaltrials.eu/search-for-clinical-trials/?lang=en&EUCT=2025-522868-32-00 |
| Date Posted: |  |
| Date of completion: |  |
| Date of first publication: |  |
| Results summary: |  |
| Baseline characteristics: |  |
| Adverse events: |  |
| Outcome measures: |  |
| IPD sharing plan: | Yes |
| IPD sharing description: | GUIDE-IT platform (www.guide-it.org) will be used for sharing clinical and imaging data after results are published. |

Disclaimer: Trials posted on this search portal are not endorsed by WHO, but are provided as a service to our users. In no event shall the World Health Organization be liable for any damages arising from the use of the information linked to in this section. None of the information obtained through use of the search portal should in any way be used in clinical care without consulting a physician or licensed health professional. WHO is not responsible for the accuracy, completeness and/or use made of the content displayed for any trial record.

Copyright - World Health Organization
- Version 3.6 -
Version history
