# Supplementary material for: Reproducibility Before Treatment Decision Rules: A Targeted Scoping Review of AI-Assisted Coronary Plaque Characterization
Source: Diagnostics (Basel). 2026 Nov 9;16(21):3703. doi: 10.3390/diagnostics16213703 (PMC13762246; doi:10.3390/diagnostics16213703)
Supplement: Supplementary file 1 [file diagnostics-16-03703-s001.zip › FileS1_addendum/registry_search/ictrp_raw/records/CTIS2025-524858-34-00.html]

ICTRP Search Portal


Home

Advanced Search

|  |  |
| --- | --- |
| List By |  |

Search Tips

|  |  |
| --- | --- |
| UTN |  |

|  |  |
| --- | --- |
| ICTRP website |  |

Contact us

List By Health Topics

List By Countries

List By Regions/Countries

UTN request

UTN validation

In English

In French

In Spanish

In Arabic

In Chinese

In Russian

Main

Note: This record shows only 24 elements of the WHO Trial Registration Data Set. To view changes that have been made to the source record, or for additional information about this trial, click on the URL below to go to the source record in the primary register.

|  |  |
| --- | --- |
| Register: | CTIS |
| Last refreshed on: | 14 September 2026 |
| Main ID: | CTIS2025-524858-34-00 |
| Date of registration: | 19/05/2026 |
| Prospective Registration: | Yes |
| Primary sponsor: | Eli Lilly & Co. |
| Public title: | A Study to See if Lepodisiran Can Reduce Plaque in Coronary Arteries of Adults With Elevated Lp(a) Who Have Had Heart Events or Are at High Risk (ACCLAIM-CTA) |
| Scientific title: | J3L-MC-EZEJ: A Phase 3, Randomized, Double-Blind, Placebo-Controlled Trial of Lepodisiran to Investigate the Effect on Coronary Plaque in Adults With Elevated Lp(a) Who Have Established Atherosclerotic Cardiovascular Disease or Are at Risk for a First Cardiovascular Event - J3L-MC-EZEJ |
| Date of first enrolment: | 14/08/2026 |
| Target sample size: | 252 |
| Recruitment status: | Recruiting |
| URL: | https://euclinicaltrials.eu/search-for-clinical-trials/?lang=en&EUCT=2025-524858-34-00 |
| Study type: | Interventional clinical trial of medicinal product |
| Study design: | Controlled: no Randomised: no Open: no Single blind: no Double blind: no Parallel group: no Cross over: no Other: no If controlled, specify comparator, Other Medicinial Product: no Placebo: no Number of treatment arms in the trial: |
| Phase: | Human pharmacology (Phase I): No Therapeutic exploratory (Phase II): No Therapeutic confirmatory - (Phase III): Yes Therapeutic use - (Phase IV): No |

|  |  |  |  |  |  |  |  |
| --- | --- | --- | --- | --- | --- | --- | --- |
| Countries of recruitment | | | | | | | |
| Australia | Czechia | Hungary | Netherlands | Spain | United Kingdom | United States |  |

Contacts

|  |  |
| --- | --- |
| Contact type: | Public |
| Name: | Lilly Clinical Trials information desk |
| Address: |  |
| Telephone: | 00353214232400 |
| Email: | EU\_lilly\_clinical\_trials@lilly.com |
| Affiliation: | Eli Lilly & Co. |

|  |  |
| --- | --- |
| Contact type: | Scientific |
| Name: | Lilly Clinical Trials information desk |
| Address: |  |
| Telephone: | 00353214232400 |
| Email: | EU\_lilly\_clinical\_trials@lilly.com |
| Affiliation: | Eli Lilly & Co. |

Key inclusion & exclusion criteria

Inclusion criteria: Have an Lp(a) =175 nmol/L, Have established cardiovascular (CV) disease or are at risk for a first CV event, Have angiographic evidence of coronary artery disease on screening CCTA, If taking lipid-lowering medications, inclusive of statins or prescription strength niacin and PCSK9 inhibitors, these should be stable for 8 weeks  
Exclusion criteria: Have had a major CV event less than 60 days before measurement of the Lp(a) level used for eligibility or uncontrolled high blood pressure at screening, Have moderate to severe renal dysfunction, Have severe heart failure, Have a history of coronary artery bypass graft surgery (CABG), Have a planned coronary angiography, percutaneous coronary intervention (PCI), coronary artery bypass graft, or valvular intervention, Have had a procedure to remove lipoproteins from the blood or received therapy specifically targeting Lp(a), Are unable to safely undergo CCTA due to medication intolerance, contrast allergies, or anatomical/technical factors  
  
Age minimum:
18  
Age maximum:
64  
Gender:
Female: yes Male: yes

Health Condition(s) or Problem(s) studied

Therapeutic area: Diseases [C] - Cardiovascular Diseases [C14]

Lipoprotein(a), Atherosclerosis, Cardiovascular Diseases

Intervention(s)

Product Name: Lepodisiran, Product Code:PRD13991605, Pharmaceutical Form: SOLUTION FOR INJECTION, Other descriptive name: , Strength: , Product Name: Placebo to match LY, Product Code:N/A, Pharmaceutical Form: N/A, Other descriptive name: N/A , Strength: , Pharmaceutical form of the placebo: N/A

Primary Outcome(s)

Secondary Outcome(s)

Secondary ID(s)

Source(s) of Monetary Support

Secondary Sponsor(s)

Ethics review

|  |  |
| --- | --- |
| Status: | Authorised |
| Approval date: | 30/07/2026 |
| Contact name: |  |
| Contact address: |  |
| Contact telephone: |  |
| Contact email: |  |

Results

|  |  |
| --- | --- |
| Results available: |  |
| URL: |  |
| URL of the protocol: | https://euclinicaltrials.eu/search-for-clinical-trials/?lang=en&EUCT=2025-524858-34-00 |
| Date Posted: |  |
| Date of completion: |  |
| Date of first publication: |  |
| Results summary: |  |
| Baseline characteristics: |  |
| Adverse events: |  |
| Outcome measures: |  |
| IPD sharing plan: | Yes |
| IPD sharing description: |  |

Disclaimer: Trials posted on this search portal are not endorsed by WHO, but are provided as a service to our users. In no event shall the World Health Organization be liable for any damages arising from the use of the information linked to in this section. None of the information obtained through use of the search portal should in any way be used in clinical care without consulting a physician or licensed health professional. WHO is not responsible for the accuracy, completeness and/or use made of the content displayed for any trial record.

Copyright - World Health Organization
- Version 3.6 -
Version history
