# Supplementary material for: Reproducibility Before Treatment Decision Rules: A Targeted Scoping Review of AI-Assisted Coronary Plaque Characterization
Source: Diagnostics (Basel). 2026 Nov 9;16(21):3703. doi: 10.3390/diagnostics16213703 (PMC13762246; doi:10.3390/diagnostics16213703)
Supplement: Supplementary file 1 [file diagnostics-16-03703-s001.zip › FileS1_addendum/registry_search/ictrp_raw/records/CTRI_2025_03_081609.html]

ICTRP Search Portal


Home

Advanced Search

|  |  |
| --- | --- |
| List By |  |

Search Tips

|  |  |
| --- | --- |
| UTN |  |

|  |  |
| --- | --- |
| ICTRP website |  |

Contact us

List By Health Topics

List By Countries

List By Regions/Countries

UTN request

UTN validation

In English

In French

In Spanish

In Arabic

In Chinese

In Russian

Main

Note: This record shows only 24 elements of the WHO Trial Registration Data Set. To view changes that have been made to the source record, or for additional information about this trial, click on the URL below to go to the source record in the primary register.

|  |  |
| --- | --- |
| Register: | CTRI |
| Last refreshed on: | 7 April 2025 |
| Main ID: | CTRI/2025/03/081609 |
| Date of registration: | 04-03-2025 |
| Prospective Registration: | Yes |
| Primary sponsor: | Kasturba Medical College Manipal Manipal Academy Of Higher Education |
| Public title: | Using Artificial Intelligence to Detect Plaques in Heart Arteries from Special Blood Vessel Scans |
| Scientific title: | Development and Validation of AI-Driven Models for Automatic Plaque Classification in Coronary Artery Disease using IVUS Imaging - NIL |
| Date of first enrolment: | 17-03-2025 |
| Target sample size: | 600 |
| Recruitment status: | Not Yet Recruiting |
| URL: | http://www.ctri.nic.in/Clinicaltrials/pmaindet2.php?trialid=126143 |
| Study type: | Observational |
| Study design: | Other Method of generating randomization sequence:Not Applicable Method of allocation concealment:Not Applicable Blinding and masking:Not Applicable |
| Phase: | N/A |

|  |  |  |  |  |  |  |  |
| --- | --- | --- | --- | --- | --- | --- | --- |
| Countries of recruitment | | | | | | | |
| India |  |  |  |  |  |  |  |

Contacts

|  |  |
| --- | --- |
| Contact type: | Public |
| Name: | Dr Ganesh P |
| Address: | Department of Cardiology, 3rd floor, Kasturba Medical College and Hospital, Madhav Nagar, Manipal  576104 Udupi, KARNATAKA India |
| Telephone: | 9914204224 |
| Email: | ganeshbmc@gmail.com |
| Affiliation: | Kasturba Medical College, Manipal, Manipal Academy of Higher Education, Manipal |

|  |  |
| --- | --- |
| Contact type: | Scientific |
| Name: | Dr Ganesh P |
| Address: | Department of Cardiology, 3rd floor, Kasturba Medical College and Hospital, Madhav Nagar, Manipal  576104 Udupi, KARNATAKA India |
| Telephone: | 9914204224 |
| Email: | ganeshbmc@gmail.com |
| Affiliation: | Kasturba Medical College, Manipal, Manipal Academy of Higher Education, Manipal |

Key inclusion & exclusion criteria

Inclusion criteria: 1. All patients undergoing angioplasty under the IVUS guidance  
Exclusion criteria: 1. Poor image quality   
  
2. Participants who refuse to give consent

Health Condition(s) or Problem(s) studied

Health Condition 1: I20-I25- Ischemic heart diseases

Intervention(s)

Intervention1: NIL: NIL  
Control Intervention1: NIL: NIL

Primary Outcome(s)

1. To develop an AI-based model for plaque classification using IVUS images.Timepoint: Baseline

Secondary Outcome(s)

1. To compare AI-based plaque type classification with human expert.   
  
2. To compare AI-based lumen segmentation with manual segmentation.   
  
Timepoint: 1. Baseline   
  
2. Baseline

Secondary ID(s)

NIL

Source(s) of Monetary Support

Kasturba Medical College and Hospital, Manipal, Manipal Academy of Higher Education, Madhav
Nagar, Manipal, Udupi, Karnataka, India - 576104

Secondary Sponsor(s)

Ethics review

|  |  |
| --- | --- |
| Status: | Approved |
| Approval date: | 19/02/2025 |
| Contact name: | KMC and KH IEC |
| Contact address: |  |
| Contact telephone: |  |
| Contact email: |  |

Results

|  |  |
| --- | --- |
| Results available: |  |
| URL: |  |
| URL of the protocol: |  |
| Date Posted: |  |
| Date of completion: |  |
| Date of first publication: |  |
| Results summary: |  |
| Baseline characteristics: |  |
| Adverse events: |  |
| Outcome measures: |  |
| IPD sharing plan: |  |
| IPD sharing description: |  |

Disclaimer: Trials posted on this search portal are not endorsed by WHO, but are provided as a service to our users. In no event shall the World Health Organization be liable for any damages arising from the use of the information linked to in this section. None of the information obtained through use of the search portal should in any way be used in clinical care without consulting a physician or licensed health professional. WHO is not responsible for the accuracy, completeness and/or use made of the content displayed for any trial record.

Copyright - World Health Organization
- Version 3.6 -
Version history
