# Supplementary material for: Reproducibility Before Treatment Decision Rules: A Targeted Scoping Review of AI-Assisted Coronary Plaque Characterization
Source: Diagnostics (Basel). 2026 Nov 9;16(21):3703. doi: 10.3390/diagnostics16213703 (PMC13762246; doi:10.3390/diagnostics16213703)
Supplement: Supplementary file 1 [file diagnostics-16-03703-s001.zip › FileS1_addendum/registry_search/ictrp_raw/records/CTRI_2025_09_094416.html]

ICTRP Search Portal


Home

Advanced Search

|  |  |
| --- | --- |
| List By |  |

Search Tips

|  |  |
| --- | --- |
| UTN |  |

|  |  |
| --- | --- |
| ICTRP website |  |

Contact us

List By Health Topics

List By Countries

List By Regions/Countries

UTN request

UTN validation

In English

In French

In Spanish

In Arabic

In Chinese

In Russian

Main

Note: This record shows only 24 elements of the WHO Trial Registration Data Set. To view changes that have been made to the source record, or for additional information about this trial, click on the URL below to go to the source record in the primary register.

|  |  |
| --- | --- |
| Register: | CTRI |
| Last refreshed on: | 15 September 2025 |
| Main ID: | CTRI/2025/09/094416 |
| Date of registration: | 08-09-2025 |
| Prospective Registration: | Yes |
| Primary sponsor: | MUSKAN YADAV |
| Public title: | Studying Heart Artery Blockages with Advanced CT Scans in Patients Having Chest Pain in Rural Central India |
| Scientific title: | CORONARY PLAQUE CHARACTERIZATION USING 128-SLICE CT ANGIOGRAPHY IN SYMPTOMATIC PATIENTS FROM RURAL CENTRAL INDIA - nil |
| Date of first enrolment: | 20-09-2025 |
| Target sample size: | 30 |
| Recruitment status: | Not Yet Recruiting |
| URL: | http://www.ctri.nic.in/Clinicaltrials/pmaindet2.php?trialid=139864 |
| Study type: | Observational |
| Study design: | Other Method of generating randomization sequence:Not Applicable Method of allocation concealment:Not Applicable Blinding and masking:Not Applicable |
| Phase: | N/A |

|  |  |  |  |  |  |  |  |
| --- | --- | --- | --- | --- | --- | --- | --- |
| Countries of recruitment | | | | | | | |
| India |  |  |  |  |  |  |  |

Contacts

|  |  |
| --- | --- |
| Contact type: | Public |
| Name: | SHIVALI KASHIKAR |
| Address: | HOUSE NO-121 BEHIND MAGANWADI RAMNAGAR WARDHA MAHARASHTRA  442007 Wardha, MAHARASHTRA India |
| Telephone: | 9822471380 |
| Email: | Shivalikashikar@gmail.com |
| Affiliation: | JAWAHARLAL NEHRU MEDICAL COLLEGE ACHARYA VINOBA BHAVE RURAL HOSPITAL SAWANGI MEGHE WARDHA |

|  |  |
| --- | --- |
| Contact type: | Scientific |
| Name: | SHIVALI KASHIKAR |
| Address: | HOUSE NO-121 BEHIND MAGANWADI RAMNAGAR WARDHA MAHARASHTRA  442007 Wardha, MAHARASHTRA India |
| Telephone: | 9822471380 |
| Email: | Shivalikashikar@gmail.com |
| Affiliation: | JAWAHARLAL NEHRU MEDICAL COLLEGE ACHARYA VINOBA BHAVE RURAL HOSPITAL SAWANGI MEGHE WARDHA |

Key inclusion & exclusion criteria

Inclusion criteria: Patients with chest pain, dyspnoea, palpitations, syncope, fatigue, reduced exercise tolerance, or a positive family history of CVD, referred for CCTA with suspected CAD, will be included   
  
Patients providing written informed consent   
  
Stable heart rate

Health Condition(s) or Problem(s) studied

Health Condition 1: I00-I99- Diseases of the circulatory system

Intervention(s)

Intervention1: Nil: Nil  
Intervention2: Nil: Nil

Primary Outcome(s)

To evaluate the prevalence and morphological characteristics of coronary plaques (calcified, non-calcified, and mixed) using 128-slice coronary CTangiography (CCTA) in symptomatic patients from rural Central India.   
  
 To assess the degree of coronary artery stenosis associated with different types of coronary plaques.   
  
To explore correlations between coronary plaque characteristics and clinical risk   
  
factors such as hypertension, diabetes, dyslipidaemia, smoking, and family   
  
history.Timepoint: To evaluate the prevalence and morphological characteristics of coronary plaques (calcified, non-calcified, and mixed) using 128-slice coronary CT angiography (CCTA) in symptomatic patients from rural Central India.

Secondary Outcome(s)

nilTimepoint: nil

Secondary ID(s)

NIL

Source(s) of Monetary Support

JAWAHARLAL NEHRU MEDICAL COLLEGE ACHARYA VINOBA BHAVE RURAL HOSPITAL SAWANGI MEGHE WARDHA

Secondary Sponsor(s)

Ethics review

|  |  |
| --- | --- |
| Status: | Approved |
| Approval date: | 15/05/2025 |
| Contact name: |  |
| Contact address: |  |
| Contact telephone: |  |
| Contact email: |  |

Results

|  |  |
| --- | --- |
| Results available: |  |
| URL: |  |
| URL of the protocol: |  |
| Date Posted: |  |
| Date of completion: |  |
| Date of first publication: |  |
| Results summary: |  |
| Baseline characteristics: |  |
| Adverse events: |  |
| Outcome measures: |  |
| IPD sharing plan: |  |
| IPD sharing description: |  |

Disclaimer: Trials posted on this search portal are not endorsed by WHO, but are provided as a service to our users. In no event shall the World Health Organization be liable for any damages arising from the use of the information linked to in this section. None of the information obtained through use of the search portal should in any way be used in clinical care without consulting a physician or licensed health professional. WHO is not responsible for the accuracy, completeness and/or use made of the content displayed for any trial record.

Copyright - World Health Organization
- Version 3.6 -
Version history
