# Supplementary material for: Reproducibility Before Treatment Decision Rules: A Targeted Scoping Review of AI-Assisted Coronary Plaque Characterization
Source: Diagnostics (Basel). 2026 Nov 9;16(21):3703. doi: 10.3390/diagnostics16213703 (PMC13762246; doi:10.3390/diagnostics16213703)
Supplement: Supplementary file 1 [file diagnostics-16-03703-s001.zip › FileS1_addendum/registry_search/ictrp_raw/records/CTRI_2026_07_113620.html]

ICTRP Search Portal


Home

Advanced Search

|  |  |
| --- | --- |
| List By |  |

Search Tips

|  |  |
| --- | --- |
| UTN |  |

|  |  |
| --- | --- |
| ICTRP website |  |

Contact us

List By Health Topics

List By Countries

List By Regions/Countries

UTN request

UTN validation

In English

In French

In Spanish

In Arabic

In Chinese

In Russian

Main

Note: This record shows only 24 elements of the WHO Trial Registration Data Set. To view changes that have been made to the source record, or for additional information about this trial, click on the URL below to go to the source record in the primary register.

|  |  |
| --- | --- |
| Register: | CTRI |
| Last refreshed on: | 3 August 2026 |
| Main ID: | CTRI/2026/07/113620 |
| Date of registration: | 07-07-2026 |
| Prospective Registration: | Yes |
| Primary sponsor: | ANKITA DROLIA |
| Public title: | A study in Indian adults using AI-assisted scans of the neck and heart blood vessels to improve the detection of heart disease |
| Scientific title: | Radiomics based multimodal evaluation of atherosclerotic disease in Indian population: Correlation of Carotid Doppler IMT with CT Coronary Angiography and machine learning - driven plaque characterisation - NIL |
| Date of first enrolment: | 19-07-2026 |
| Target sample size: | 146 |
| Recruitment status: | Not Yet Recruiting |
| URL: | http://www.ctri.nic.in/Clinicaltrials/pmaindet2.php?trialid=154587 |
| Study type: | Observational |
| Study design: | Other Method of generating randomization sequence:Not Applicable Method of allocation concealment:Not Applicable Blinding and masking:Not Applicable |
| Phase: | N/A |

|  |  |  |  |  |  |  |  |
| --- | --- | --- | --- | --- | --- | --- | --- |
| Countries of recruitment | | | | | | | |
| India |  |  |  |  |  |  |  |

Contacts

|  |  |
| --- | --- |
| Contact type: | Public |
| Name: | Dr Priya P S |
| Address: | Department of radiodiagnosis and imaging kasturba medical college and hospital MAHE Manipal Udupi 576104 Karnataka  576104 Udupi, KARNATAKA India |
| Telephone: | 9895563264 |
| Email: | priya.ps@manipal.edu |
| Affiliation: | kasturba medical college and hospital manipal MAHE |

|  |  |
| --- | --- |
| Contact type: | Scientific |
| Name: | Dr Priya P S |
| Address: | Department of radiodiagnosis and imaging kasturba medical college and hospital MAHE Manipal Udupi 576104 Karnataka  576104 Udupi, KARNATAKA India |
| Telephone: | 9895563264 |
| Email: | priya.ps@manipal.edu |
| Affiliation: | kasturba medical college and hospital manipal MAHE |

Key inclusion & exclusion criteria

Inclusion criteria: Indian adults aged 18 years or older   
  
   
  
Individuals undergoing CT coronary angiography and carotid Doppler ultrasonography on the same day as part of the study protocol   
  
   
  
Willing and able to provide written informed consent to participate in the study.

Health Condition(s) or Problem(s) studied

Health Condition 1: I708- Atherosclerosis of other arteries

Intervention(s)

Intervention1: Nil: Nil

Primary Outcome(s)

1 To assess the relationship between carotid IMT on Doppler ultrasound and CAD severity on CT coronary angiography   
  
   
  
2 To characterize plaque and utilize radionics for plaque analysis based on CT coronary angiography imagesTimepoint:   
  
Baseline or day 0   
  
   
  
All study imaging including CT coronary angiography and carotid Doppler ultrasonography will be performed on the same day   
  
   
  
Outcome measures will be derived from these baseline images, with offline radiomic and AI-based image analysis performed subsequently

Secondary Outcome(s)

To evaluate radiomics-based ML models in differentiating significant, intermediate and non-significant CADTimepoint: baseline

Secondary ID(s)

NIL

Source(s) of Monetary Support

KASTURBA MEDICAL COLLEGE AND HOSPITAL
MAHE
MANIPAL
UDUPI
KARNATAKA
576104
INDIA

Secondary Sponsor(s)

Ethics review

|  |  |
| --- | --- |
| Status: | Approved |
| Approval date: | 06/11/2025 |
| Contact name: |  |
| Contact address: |  |
| Contact telephone: |  |
| Contact email: |  |

Results

|  |  |
| --- | --- |
| Results available: |  |
| URL: |  |
| URL of the protocol: |  |
| Date Posted: |  |
| Date of completion: |  |
| Date of first publication: |  |
| Results summary: |  |
| Baseline characteristics: |  |
| Adverse events: |  |
| Outcome measures: |  |
| IPD sharing plan: |  |
| IPD sharing description: |  |

Disclaimer: Trials posted on this search portal are not endorsed by WHO, but are provided as a service to our users. In no event shall the World Health Organization be liable for any damages arising from the use of the information linked to in this section. None of the information obtained through use of the search portal should in any way be used in clinical care without consulting a physician or licensed health professional. WHO is not responsible for the accuracy, completeness and/or use made of the content displayed for any trial record.

Copyright - World Health Organization
- Version 3.6 -
Version history
