# Supplementary material for: Reproducibility Before Treatment Decision Rules: A Targeted Scoping Review of AI-Assisted Coronary Plaque Characterization
Source: Diagnostics (Basel). 2026 Nov 9;16(21):3703. doi: 10.3390/diagnostics16213703 (PMC13762246; doi:10.3390/diagnostics16213703)
Supplement: Supplementary file 1 [file diagnostics-16-03703-s001.zip › FileS1_addendum/registry_search/ictrp_raw/records/DRKS00037560.html]

ICTRP Search Portal


Home

Advanced Search

|  |  |
| --- | --- |
| List By |  |

Search Tips

|  |  |
| --- | --- |
| UTN |  |

|  |  |
| --- | --- |
| ICTRP website |  |

Contact us

List By Health Topics

List By Countries

List By Regions/Countries

UTN request

UTN validation

In English

In French

In Spanish

In Arabic

In Chinese

In Russian

Main

Note: This record shows only 24 elements of the WHO Trial Registration Data Set. To view changes that have been made to the source record, or for additional information about this trial, click on the URL below to go to the source record in the primary register.

|  |  |
| --- | --- |
| Register: | German Clinical Trials Register |
| Last refreshed on: | 30 March 2026 |
| Main ID: | DRKS00037560 |
| Date of registration: | 29/07/2025 |
| Prospective Registration: | Yes |
| Primary sponsor: | MVZ CCB Frankfurt und Main-Taunus GbR |
| Public title: | Comparison of Prognostic Utility and Additive Value of Quantitative Coronary Plaque Volumes Assessed with Coronary Computed Tomography Angiography and Quantitative Myocardial Ischemia Assessed by Cardiac Magnetic Resonance Imaging |
| Scientific title: | Comparison of Prognostic Utility and Additive Value of Quantitative Coronary Plaque Volumes Assessed with Coronary Computed Tomography Angiography and Quantitative Myocardial Ischemia Assessed by Cardiac Magnetic Resonance Imaging - COPRODUCTION registry |
| Date of first enrolment: | 01/08/2025 |
| Target sample size: | 10000 |
| Recruitment status: | Recruiting |
| URL: | http://drks.de/search/en/trial/DRKS00037560 |
| Study type: | observational |
| Study design: | Allocation: ; Masking: ; Control: ; Assignment: ; Study design purpose: prevention |
| Phase: |  |

|  |  |  |  |  |  |  |  |
| --- | --- | --- | --- | --- | --- | --- | --- |
| Countries of recruitment | | | | | | | |
| Germany |  |  |  |  |  |  |  |

Contacts

|  |  |
| --- | --- |
| Contact type: | public |
| Name: | Philipp    Breitbart |
| Address: | Im Prüfling 23 60389 Frankfurt am Main Germany |
| Telephone: | +49 69 9450280 |
| Email: | p.breitbart@ccb.de |
| Affiliation: | MVZ CCB Frankfurt und Main-Taunus GbR |

|  |  |
| --- | --- |
| Contact type: | scientific |
| Name: | Philipp    Breitbart |
| Address: | Im Prüfling 23 60389 Frankfurt am Main Germany |
| Telephone: | +49 69 9450280 |
| Email: | p.breitbart@ccb.de |
| Affiliation: | MVZ CCB Frankfurt und Main-Taunus GbR |

Key inclusion & exclusion criteria

Inclusion criteria: 1. Coronary CT (with photon-counting CT) based on clinical indication
  
2. Written consent for the pseudonymized use of clinical, procedural, and follow-up data

Health Condition(s) or Problem(s) studied

I25

Chronic ischaemic heart disease

Intervention(s)

Group 1: Patients who underwent coronary computed tomography angiography for a clinical indications.

Primary Outcome(s)

Assessment and quantification of coronary plaque volume (total and per vessel) as well as plaque characteristics (including potential vulnerability criteria) using coronary CT angiography at the time of study inclusion, and evaluation of a correlation between plaque burden/composition and the extent of cardiovascular risk factors (in particular LDL cholesterol and lipoprotein(a) levels).

Secondary Outcome(s)

Major adverse cardiac events (composite event rate including coronary revascularization, myocardial infarction, cardiac or cardiovascular death, and all-cause mortality) during follow-up after coronary CT angiography.

Secondary ID(s)

2021-2543 2-evBO

Source(s) of Monetary Support

MVZ CCB Frankfurt und Main-Taunus GbR

Secondary Sponsor(s)

Ethics review

|  |  |
| --- | --- |
| Status: | Approved |
| Approval date: | 22/07/2025 |
| Contact name: | ethikkommission@laekh.de |
| Contact address: | ethikkommission@laekh.de |
| Contact telephone: | +49-69-97672209 |
| Contact email: | ethikkommission@laekh.de |

Results

|  |  |
| --- | --- |
| Results available: |  |
| URL: | http://drks.de/search/en/trial/DRKS00037560#studyResults |
| URL of the protocol: | http://drks.de/search/en/trial/DRKS00037560#studyProtocols |
| Date Posted: |  |
| Date of completion: |  |
| Date of first publication: |  |
| Results summary: |  |
| Baseline characteristics: | http://drks.de/search/en/trial/DRKS00037560#basicReporting |
| Adverse events: | http://drks.de/search/en/trial/DRKS00037560#basicReporting |
| Outcome measures: | http://drks.de/search/en/trial/DRKS00037560#basicReporting |
| IPD sharing plan: | No |
| IPD sharing description: |  |

Disclaimer: Trials posted on this search portal are not endorsed by WHO, but are provided as a service to our users. In no event shall the World Health Organization be liable for any damages arising from the use of the information linked to in this section. None of the information obtained through use of the search portal should in any way be used in clinical care without consulting a physician or licensed health professional. WHO is not responsible for the accuracy, completeness and/or use made of the content displayed for any trial record.

Copyright - World Health Organization
- Version 3.6 -
Version history
