# Supplementary material for: Reproducibility Before Treatment Decision Rules: A Targeted Scoping Review of AI-Assisted Coronary Plaque Characterization
Source: Diagnostics (Basel). 2026 Nov 9;16(21):3703. doi: 10.3390/diagnostics16213703 (PMC13762246; doi:10.3390/diagnostics16213703)
Supplement: Supplementary file 1 [file diagnostics-16-03703-s001.zip › FileS1_Search-Strategy_rev2-edited.pdf]

File S1. Verbatim search strategy and record accounting

Reproducibility Before Treatment Decision Rules: A Targeted Scoping Review of AI-Assisted Coronary Plaque Characterization

1. Search parameters

Database. MEDLINE/PubMed.

Interface. NCBI E-utilities (esearch/esummary/efetch).

Date executed. 24 July 2026—a single date; searches were not re-run before submission.

Queries. 97, organized into 16 thematic blocks across two rounds.

Publication-date limits. Round 1: 2019–2026. Round 2: 2018–2026. Applied uniformly to every block within each round (datetype=pdat).

Sort/retrieval. sort=relevance; retmax=40 per query.

Language filter. None applied. Every record meeting the eligibility criteria was published in English.

2. Record accounting

Counts below are recomputed from the raw returns and reconcile with Figure 2 of the manuscript.

Records returned across all 97 queries (with duplicates)	**1,773**
Duplicate records removed	**292**
Unique records	**1,481**
Removed by the automated eligibility filter	**66**
—no abstract and not indexed as a review	64
—erratum or retraction notices	2
Records listed for AI title review (shortlist.txt)	**1,415**
Records included at first submission, before 13 physiology-only records were reclassified	**134**

3. Automated eligibility filter

Applied to all deduplicated records before the AI title review (triage.py; Section 2.3 of the manuscript). A record was removed if any of the following held:

- no publication year recorded;
- publication year before 2015;
- title matching /retracted|erratum|correction to|author correction/i;
- no abstract available and publication type not indexed as Review.

The pre-2015 criterion removed no records, since both rounds were already date-limited at source; the earliest record returned was published in 2018.

4. Verbatim queries

Reproduced exactly as submitted to the E-utilities interface.

Round 1—search.py, publication dates 2019–2026

A_ccta_ai_plaque (5 queries)

```
artificial intelligence coronary CT angiography plaque quantification
deep learning coronary plaque characterization CCTA
automated plaque quantification coronary computed tomography angiography validation
machine learning atherosclerotic plaque burden coronary CT prognosis
AI-QCT quantitative coronary computed tomography
```

B_ffrct (4 queries)

```
FFR-CT deep learning fractional flow reserve computed tomography
machine learning FFRCT diagnostic accuracy coronary
HeartFlow FFRCT clinical outcomes
on-site CT-derived fractional flow reserve validation
```

C_pcct_spectral (4 queries)

```
photon-counting detector CT coronary plaque
photon counting CT coronary artery imaging quantification
spectral CT coronary plaque characterization dual-energy
photon-counting CT radiomics
```

D_groups (7 queries)

```
Kolossvary radiomics coronary plaque
Maurovich-Horvat coronary CT plaque
Antoniades perivascular fat attenuation index
Oikonomou perivascular fat radiomic
Dey machine learning cardiac imaging plaque
Slomka artificial intelligence cardiac imaging
Lin AI quantitative plaque coronary CT
```

E_commercial (7 queries)

```
Cleerly coronary plaque analysis validation
CLEERLY CCTA artificial intelligence outcomes
Elucid vascuCAP plaque histology validation
Caristo fat attenuation index CaRi-Heart validation
Artrya Salix coronary artificial intelligence
Circle Cardiovascular Imaging cvi42 validation
HeartFlow plaque analysis validation study
```

F_digitaltwin_fusion (4 queries)

```
digital twin cardiovascular imaging
multimodal fusion deep learning cardiac imaging
digital twin coronary artery hemodynamics patient-specific
foundation model cardiac imaging multimodal
```

G_health_econ (4 queries)

```
cost-effectiveness coronary CT angiography artificial intelligence
FFRCT cost-effectiveness invasive coronary angiography reduction
economic evaluation AI cardiac imaging
downstream testing reduction coronary CT decision support
```

H_regulatory (4 queries)

```
FDA clearance artificial intelligence cardiovascular imaging devices
regulatory artificial intelligence medical imaging 510(k) review
generalizability external validation deep learning medical imaging
radiomics reproducibility repeatability CT
```

I_background (5 queries)

```
SCOT-HEART coronary CT angiography outcomes
PROMISE trial coronary CT angiography
high-risk plaque features adverse cardiac events CT
low attenuation plaque burden myocardial infarction
pericoronary adipose tissue attenuation prognosis
```

Round 2—search2.py, publication dates 2018–2026

D2_authors (9 queries)

```
Kolossvary M[au] AND (coronary OR radiomics OR plaque)
Maurovich-Horvat P[au] AND (coronary OR plaque OR CT)
Antoniades C[au] AND (coronary OR perivascular OR inflammation)
Oikonomou EK[au] AND (coronary OR cardiovascular OR imaging)
Dey D[au] AND (plaque OR coronary OR "machine learning")
Slomka PJ[au] AND ("artificial intelligence" OR "deep learning" OR "machine learning")
Lin A[au] AND (plaque OR coronary CT OR "machine learning")
Nakanishi R[au] AND coronary plaque
Nurmohamed NS[au] AND (plaque OR coronary)
```

E2_commercial (19 queries)

```
"Cleerly"[All Fields]
CONFIRM2 trial coronary artificial intelligence plaque
artificial intelligence quantitative coronary plaque analysis agreement intravascular ultrasound
Elucid[All Fields] AND (plaque OR coronary)
vascuCAP
CaRi-Heart
ORFAN cohort perivascular fat attenuation cardiac risk
Artrya[All Fields]
Salix coronary anatomy artificial intelligence Australia
cvi42 OR "Circle Cardiovascular Imaging"
DeepVessel FFR OR "CT-FFR" China multicenter deep learning
FFRCT ADVANCE registry
PACIFIC trial FFRCT diagnostic performance
NXT trial FFRCT diagnostic performance HeartFlow
DISCHARGE trial coronary CT
PRECISE trial precision strategy chest pain
TARGET trial CT-derived FFR randomized
FASTTRACK CABG coronary CT planning
P3 study artificial intelligence plaque progression statin
```

G2_econ (5 queries)

```
cost-effectiveness FFRCT stable chest pain
FFRCT economic outcomes PLATFORM
cost effectiveness artificial intelligence coronary plaque analysis
budget impact analysis cardiac imaging artificial intelligence
reduction unnecessary invasive coronary angiography CT guided
```

H2_reg (7 queries)

```
FDA authorized artificial intelligence machine learning medical devices radiology
De Novo pathway artificial intelligence medical device cardiology
external validation artificial intelligence coronary CT multicenter
radiomics quality score coronary
dataset shift bias artificial intelligence medical imaging equity
CONSORT-AI SPIRIT-AI reporting guideline
CLAIM checklist artificial intelligence medical imaging reporting
```

C2_pcct (4 queries)

```
photon-counting CT calcium blooming stent coronary ultra-high resolution
photon counting detector CT coronary CTA image quality clinical first
photon-counting CT plaque volume quantification accuracy phantom
spectral photon counting CT atherosclerotic plaque ex vivo
```

F2_fusion (4 queries)

```
digital twin heart clinical translation
in silico trial cardiovascular device virtual patient
transformer multimodal electronic health record imaging cardiovascular risk
CT MRI fusion myocardial ischemia artificial intelligence
```

I2_bg (5 queries)

```
ISCHEMIA trial invasive conservative
plaque burden progression statin therapy serial CT
napkin ring sign prognosis
CAD-RADS 2.0
coronary inflammation imaging biomarker prediction cardiac mortality
```

5. Reproduction

The search is reproducible end-to-end with:

```
python3 search.py && python3 search2.py && python3 triage.py
```

search.py and search2.py execute the two rounds and write raw_results.json and raw_results2.json; triage.py merges both rounds, deduplicates on PMID, applies the eligibility filter in Section 3 above and writes candidates.json (1,415 records) together with the grouped shortlist used for screening. The scripts and raw returns are in the folder FileS1_addendum/search_july2026; the only change from the files as run is that the contact e-mail address required by NCBI has been replaced by a placeholder.

Because PubMed indexing changes over time, re-executing these queries after 24 July 2026 will return a larger record set than the counts in Section 2.

Vendor-search outcome note: Circle Cardiovascular Imaging (cvi42)

The two vendor queries above (rounds 1 and 2) returned no study validating this vendor's coronary plaque quantification against an intravascular, histological or expert reference standard. The software does appear in the coronary CT literature as the measurement tool rather than as the object of study. Records identified in which Circle Cardiovascular Imaging (cvi42) was used to derive CCTA plaque or pericoronary adipose tissue measurements, without reported validation of those measurements against a reference standard: PMID 31143662; 35773708; 36915301; 39039597; 40001233. One of these (PMID 35773708) reports intra- and inter-observer reproducibility only. This note supports Section 3.7 of the manuscript.

Confirmatory search, 15 September 2026

This search was run after the review was drafted to test how reproducible the thematic searches of 24 July 2026 are. At the first revision, its additional records were not screened; at the second revision, all of them were screened (Section 2.3 of the manuscript; addendum C below; File S7).

MEDLINE/PubMed

Database: MEDLINE/PubMed via NCBI E-utilities esearch (db=pubmed, usehistory=y, POST). Run 15 September 2026, 12:47 PDT.

Publication-date limit 2018-01-01 to 2026-09-15 (datetype=pdat). No language filter. No record cap: all results were paged with WebEnv/retstart.

Count: 4,058 records. Block counts with the same date limit: modality 430,086; method 632,626; target 201,342.

Verbatim query string, three blocks combined with AND:

```
("Computed Tomography Angiography"[Mesh] OR "Tomography, X-Ray Computed"[Mesh] OR
"Coronary Angiography"[Mesh] OR CCTA[tiab] OR CTA[tiab] OR "computed tomograph*"[tiab]
OR "CT angiograph*"[tiab] OR "coronary CT"[tiab] OR "cardiac CT"[tiab] OR "CT"[tiab] OR
"photon-counting"[tiab] OR "photon counting"[tiab] OR "dual-energy"[tiab] OR "spectral
CT"[tiab] OR FFRCT[tiab] OR "FFR-CT"[tiab] OR "CT-FFR"[tiab] OR "FFR CT"[tiab])

AND

("Artificial Intelligence"[Mesh] OR "Machine Learning"[Mesh] OR "Deep Learning"[Mesh]
OR "Neural Networks, Computer"[Mesh] OR "Radiographic Image Interpretation, Computer-
Assisted"[Mesh] OR "artificial intelligence"[tiab] OR "machine learning"[tiab] OR "deep
learning"[tiab] OR "neural network*"[tiab] OR convolutional[tiab] OR radiomic*[tiab] OR
"texture analys*"[tiab] OR automat*[tiab] OR "semi-automat*"[tiab] OR
semiautomat*[tiab] OR "AI-QCT"[tiab] OR "AI-based"[tiab] OR "AI-enabled"[tiab] OR "AI-
guided"[tiab] OR "AI-driven"[tiab] OR "computer-aided"[tiab] OR "computational fluid
dynamic*"[tiab] OR FFRCT[tiab] OR "FFR-CT"[tiab] OR "CT-FFR"[tiab] OR "CT-derived
fractional flow reserve"[tiab] OR "fat attenuation index"[tiab] OR
radiotranscriptomic*[tiab] OR Cleerly[tiab] OR HeartFlow[tiab] OR Elucid[tiab] OR
vascuCAP[tiab] OR "CaRi-Heart"[tiab] OR Artrya[tiab] OR cvi42[tiab] OR "Circle
Cardiovascular"[tiab])

AND

("Plaque, Atherosclerotic"[Mesh] OR "Coronary Artery Disease"[Mesh] OR "Coronary
Disease"[Mesh] OR "Coronary Stenosis"[Mesh] OR "Coronary Vessels"[Mesh] OR "Fractional
Flow Reserve, Myocardial"[Mesh] OR "Atherosclerosis"[Mesh] OR plaque*[tiab] OR
atheroscl*[tiab] OR atherom*[tiab] OR "coronary artery disease"[tiab] OR "coronary
stenos*"[tiab] OR pericoronary[tiab] OR "peri-coronary"[tiab] OR perivascular[tiab] OR
"fat attenuation"[tiab] OR "fractional flow reserve"[tiab] OR FFRCT[tiab] OR "FFR-
CT"[tiab] OR "CT-FFR"[tiab])
```

Europe PMC

Database: Europe PMC REST API (searchPOST, cursorMark paging, resultType=lite). Run 15 September 2026, 12:55 PDT. Date limit FIRST_PDATE:[2018-01-01 TO 2026-09-15].

The same three term blocks were used as TITLE_ABS free text, without MeSH clauses: Europe PMC's MH field is sparse, and adding MH clauses reduced rather than increased the counts. Block counts: modality 387,705; method 659,579; target 168,255.

Count: 2,784 records (MEDLINE 2,633, preprints 135, theses 6, Agricola 5, PMC 5). All 2,633 MEDLINE records were already in the PubMed set, so Europe PMC contributed no new PubMed identifiers.

Scopus and Embase were not searched; they require institutional access. The PubMed string above is written so that it can be translated directly to Emtree.

Record counts

PubMed 4,058 and Europe PMC 2,784, total 6,842; less 2,633 records held by both sources, giving 4,209 records by identifier (4,058 with a PubMed identifier and 151 without; at most 4,159 unique because 50 of the 151 duplicate PubMed records by title, as found at the second revision).

Of these, 424 had already been identified by the searches of 24 July 2026, including 79 of the 134 included records, leaving 3,785 records not previously identified.

Recall against the included set: 79 of 134 (59.0%) overall and 79 of the 81 included records that fall inside all three concept blocks (97.5%). The remainder are background, regulatory, reporting-guideline and detector-physics records that lie outside the artificial-intelligence block by design.

A deterministic rule-based pre-screen of the 3,785 new records labeled 1,431 potentially eligible, 829 uncertain and 1,525 ineligible; 1,364 of the potentially eligible records had been indexed in MEDLINE on or before 24 July 2026. The pre-screen is a prioritization and not an eligibility decision: on a read sample, its precision was 68-82%. This pre-screen was superseded at the second revision by a two-pass AI screen of all 3,634 PubMed records with author decisions on unresolved records (addendum C; File S7).

Per-record outputs

The folder FileS1_addendum/confirmatory_search contains: structured_search_pmids.txt, the 4,058 PubMed identifiers; merge_counts.json, the de-duplication and recall counts; structured_search_run.json and epmc_structured_run.json, the executed queries; and pubmed_structured.py and epmc_structured.py, the search scripts (contact e-mail replaced by a placeholder). Every one of the 3,785 additional records is listed with its screening decisions in File S7. The superseded rule-based pre-screen file is available from the corresponding author on request.

Addendum (revision, October 2026)

A. Trial-registry search (8 October 2026)

ClinicalTrials.gov and the WHO ICTRP were searched for randomized trials of AI-based coronary plaque analysis on CCTA with clinical endpoints. Query strings, raw returns and the screened list are provided in the folder FileS1_addendum/registry_search.

B. Re-screen of records not selected at the title stage

The 1,280 records not selected were re-screened on title and abstract by one AI pass (Stage 2) under the retained protocol (FileS1_addendum/recall_check/PROTOCOL.md, including dated amendments), which flagged 125. A second, blind AI pass (Stage 3) assessed the 125 flagged records and a seeded random sample of 50 Stage 2 exclusions (175 records; agreement 144 of 175, Cohen's κ 0.68; AGREEMENT.md); its decisions were time-stamped and hashed before comparison (LOG.md). Stage 3 was run as a second, blind AI pass rather than the human adjudication planned in section 1 of the protocol (see manuscript Section 2.3), and reference numbers in the frozen protocol refer to the first-submission reference list. Decisions for every record, the agreement analysis and the reconciliation log are provided. The protocol and log are reproduced as frozen in October 2026, so they carry the manuscript's working title and the internal file paths in use at that time. Public copies differ from the frozen originals only in working-file and working-folder names, which are replaced by [working file] or [working folder], one reference to the orchestrating agent, replaced by "an instruction to the agent", and the corresponding author's informal first name, removed; originals are available from the corresponding author on request.

C. Screen of the confirmatory-search records (second revision)

The 3,785 records added by the confirmatory search were screened under a separate protocol fixed and hashed before screening. The screener instruction, the decision files of both AI passes, the agreement statistics, the exclusion counts and the PRISMA counts are in the folder FileS1_addendum/screen3785, whose README.md summarizes the protocol and lists every protocol deviation. The full screening protocol and the adjudication working file are available from the corresponding author on request. Per-record decisions are in File S7. Public copies differ from the frozen originals only in working-file and working-folder names, which are replaced by [working file] or [working folder] (and one reference to the orchestrating agent, replaced by "an instruction to the agent"); originals are available from the corresponding author on request.
