# Supplementary material for: Reproducibility Before Treatment Decision Rules: A Targeted Scoping Review of AI-Assisted Coronary Plaque Characterization
Source: Diagnostics (Basel). 2026 Nov 9;16(21):3703. doi: 10.3390/diagnostics16213703 (PMC13762246; doi:10.3390/diagnostics16213703)
Supplement: Supplementary file 1 [file diagnostics-16-03703-s001.zip › FileS2_PRISMA-ScR-Checklist_rev2-edited.pdf]

File S2 (rev3). PRISMA-ScR Checklist

Reproducibility Before Treatment Decision Rules: A Targeted Scoping Review of AI-Assisted Coronary Plaque Characterization

Preferred Reporting Items for Systematic reviews and Meta-Analyses extension for Scoping Reviews (PRISMA-ScR) Checklist. Tricco AC, Lillie E, Zarin W, et al. PRISMA extension for scoping reviews (PRISMA-ScR): checklist and explanation. Ann Intern Med. 2018;169:467–473. doi:10.7326/M18-0850

Section	#	PRISMA-ScR checklist item	Reported in (page of the revised manuscript)
**TITLE**	1	**Title.** Identify the report as a scoping review.	Title page, p. 1—"Reproducibility Before Treatment Decision Rules: A Targeted Scoping Review of AI-Assisted Coronary Plaque Characterization".
**ABSTRACT**	2	**Structured summary.** Provide a structured summary that includes (as applicable): background, objectives, eligibility criteria, sources of evidence, charting methods, results, and conclusions that relate to the review questions and objectives.	Abstract, pp. 1–2—Structured under Background/Objectives, Methods, Results and Conclusions; states reporting according to PRISMA-ScR, the source and timing of the search (July 2026, supplemented by a confirmatory search in September 2026), the AI title-only selection and how records were re-screened and screened, and the numbers of records assessed, charted (236) and mapped from their abstracts (307).
**INTRODUCTION**	3	**Rationale.** Describe the rationale for the review in the context of what is already known. Explain why the review questions/objectives lend themselves to a scoping review approach.	Section 1, pp. 2–6; Figure 1, p. 5; rationale for the scoping form in Section 2.1, p. 6.
	4	**Objectives.** Provide an explicit statement of the questions and objectives being addressed with reference to their key elements (e.g., population or participants, concepts and context) or other relevant key elements used to conceptualize the review questions and/or objectives.	Section 1, final paragraph, p. 4 (review questions framed as population, concept and context); Section 2.1, p. 6.
**METHODS**	5	**Protocol and registration.** Indicate whether a review protocol exists; state if and where it can be accessed (e.g., a Web address); and if available, provide registration information, including the registration number.	Section 2.1, p. 6—The review protocol was not registered in a public registry, and no separate protocol was published; the methods are reported in full in Sections 2.2–2.7 (pp. 6–15) and File S1. The written protocol of the re-screen of records not selected is retained in full in File S1; the protocol of the screen of the confirmatory-search records is summarized, with its deviations, in File S1, and the full protocol is available from the corresponding author on request (Section 2.3, pp. 8–13).
	6	**Eligibility criteria.** Specify characteristics of the sources of evidence used as eligibility criteria (e.g., years considered, language and publication status), and provide a rationale.	Section 2.3, pp. 8–13 (inclusion and exclusion criteria, with rationale; physiology-only readouts excluded explicitly at revision); Section 2.2, pp. 6–8 (publication-date limits 2019–2026 in search round 1 and 2018–2026 in round 2, 2018 to 15 September 2026 in the confirmatory search; no language restriction at the search stage).
	7	**Information sources.** Describe all information sources in the search (e.g., databases with dates of coverage and contact with authors to identify additional sources), as well as the date the most recent search was executed.	Section 2.2, pp. 6–8—MEDLINE/PubMed via the NCBI E-utilities API, thematic searches executed on 24 July 2026; a confirmatory structured search of MEDLINE/PubMed and Europe PMC, 15 September 2026; trial registers ClinicalTrials.gov (API v2) and the WHO ICTRP, searched on 8 October 2026; six records added by the authors outside the search pipeline, and four records from the confirmatory search added by the authors before it was screened (Section 2.2; Figure 2, p. 11).
	8	**Search.** Present the full electronic search strategy for at least 1 database, including any limits used, such that it could be repeated.	Section 2.2, pp. 6–8—Free-text PubMed queries covering three concepts, relevance-sorted with at most 40 records per query, publication-date limits and vendor product and company-name queries; the confirmatory structured query (controlled-vocabulary and title/abstract terms, no record cap) and the registry searches are described in the same section. File S1 gives the verbatim query strings for

			all 97 searches with the scripts and raw returns, the confirmatory query with its counts and the registry query strings.
	9	**Selection of sources of evidence.** State the process for selecting sources of evidence (i.e., screening and eligibility) included in the scoping review.	Section 2.3, pp. 8–13—An automated filter that applied no eligibility criteria; the titles of the remaining 1,415 records were screened by an AI agent under selection rules set by the corresponding author, and it selected 135 for assessment (no relevance score or pre-specified cut-off was used; Section 2.7, pp. 14–15); title-and-abstract evaluation of the 141 records (135 selected + 6 added by the authors) by the corresponding author, without independent duplicate screening; full texts not used for selection. The 1,280 records not selected were re-screened by one AI pass under a written protocol, with a second, blind AI pass on the 125 records it flagged and a random sample of 50 of its exclusions, and the AI-recommended decisions on records the protocol did not settle were accepted by the corresponding author at the second revision. Of the 3,785 records of the confirmatory search, 50 duplicates, and 101 rule-based exclusions were removed before the remaining 3,634 were screened by two independent AI passes (Cohen's κ 0.931), and the 335 records the passes did not settle were decided by the corresponding author, who accepted written AI recommendations in a single instruction. Figure 2, p. 11.
	10	**Data charting process.** Describe the methods of charting data from the included sources of evidence (e.g., calibrated forms or forms that have been tested by the team before their use and whether data charting was done independently or in duplicate) and any processes for obtaining and confirming data from investigators.	Section 2.6, pp. 13–14—Charting by AI-assisted extraction (Section 2.7, pp. 14–15); for the 121 records charted at submission, there was no independent duplicate extraction, and at the second revision, an AI verification step re-checked every value against the source, with the corrections accepted by the corresponding author; the records added at revision were charted by two independent AI extraction passes, reconciled against the source by a further AI session and not checked field by field by an author; the 307 records added by the confirmatory-search screen were mapped from their abstracts by two independent AI sessions (Section 2.3). The charted dataset is Table S1; the mapped records are in File S7.
	11	**Data items.** List and define all variables for which data were sought and any assumptions and simplifications made.	Section 2.6, pp. 13–14—Full list of charted fields; the vendor-attribution hierarchy is defined in Section 2.4, p. 13; codebook and charted dataset in Table S1.
	12	**Critical appraisal of individual sources of evidence.** If done, provide a rationale for conducting a critical appraisal of included sources of evidence; describe the methods used and how this information was used in any data synthesis (if appropriate).	Section 2.5, p. 13—No formal risk-of-bias instrument was applied; each source was appraised against the specific claim it was cited to support, against seven stated criteria.
	13	**Synthesis of results.** Describe the methods of handling and summarizing the data that were charted.	Sections 2.5 and 2.6, pp. 13–14—Narrative synthesis along a four-stage translational axis; the evidence map (Figure 1) is scored with a stated rule (File S4), and a sensitivity analysis specified before the added records were scored is given in File S6.
**RESULTS**	14	**Selection of sources of evidence.** Give numbers of sources of evidence screened, assessed for eligibility and included in the review, with reasons for exclusions at each stage, ideally using a flow diagram.	Figure 2, p. 11, and Section 2.3, pp. 8–13—July 2026 search: 1,773 records identified; 292 duplicates and 66 records removed by the automated filter; the titles of 1,415 records reviewed by an AI agent, 135 selected and 1,280 not selected; 135 assessed on title and abstract, 6 excluded (reasons not recorded) and 129 included; 6 records added by the authors, 1 excluded (outside scope) and 5 included; 13 physiology-only records reclassified as context, leaving 121. Re-screen of the 1,280 records not selected: 127 potentially eligible; on full charting 110

			included and 17 excluded, with reasons. Four records from the confirmatory search added by the authors. Screen of the confirmatory search: 3,785 records, of which 50 duplicates and 101 records excluded by rule were removed, and 3,634 were screened; 3,054 excluded by both AI passes and 266 by the author, with reasons; 314 eligible, of which 6 were already charted, 1 was charted from its full text, and 307 were mapped from their abstracts. In total, 236 charted records and 307 mapped records (543 records; no records merged under the report-grouping rule).
	15	**Characteristics of sources of evidence.** For each source of evidence, present characteristics for which data were charted and provide the citations.	Table 1, p. 17 (platforms, reference standards and evidence stage); Table 2, p. 24 (thresholds and derivation conditions); Table 3, p. 27 (reproducibility); Sections 3–7, pp. 15–40; charted dataset for all 236 charted records in Table S1; Figure 1 fields for the 307 mapped records in File S7.
	16	**Critical appraisal within sources of evidence.** If done, present data on critical appraisal of included sources of evidence (see item 12).	Sections 3.1–3.8, pp. 15–22 (independence from the platform developer stated at each point of use); Section 6, pp. 39–40; Section 8, pp. 40–43.
	17	**Results of individual sources of evidence.** For each included source of evidence, present the relevant data that were charted that relate to the review questions and objectives.	Sections 3–7, pp. 15–40; Table 1, p. 17; Table 2, p. 24; Table 3, p. 27; Figure 4, p. 26; Figures 6–9, pp. 32–39; Table S1; File S7 (Figure 1 fields of the 307 abstract-mapped records).
	18	**Synthesis of results.** Summarize and/or present the charting results as they relate to the review questions and objectives.	Figure 1, p. 5 (evidence status across five claim domains and four translational stages; scoring rule and cell-by-cell justification in File S4; sensitivity analysis in File S6); Sections 5–7, pp. 29–40; Figures 6–9, pp. 32–39.
**DISCUSSION**	19	**Summary of evidence.** Summarize the main results (including an overview of concepts, themes and types of evidence available), link to the review questions and objectives, and consider the relevance to key groups.	Section 9.1, pp. 43–45.
	20	**Limitations.** Discuss the limitations of the scoping review process.	Section 8, pp. 40–43—No step performed in independent duplicate by two human reviewers; for the original thematic searches, a single search date (24 July 2026), a single database and at most 40 records per query (the uncapped confirmatory search of 15 September 2026 added Europe PMC); records selected for assessment by an AI agent's judgement on truncated titles, with no retained prompt, rather than by manual title screening; eligibility assessed on title and abstract; exclusion reasons not recorded for six records; six records added outside the search; the limitations of the AI screen of the confirmatory-search records (both passes used the same model, so errors common to both cannot be detected; the only check on the shared exclusions is an AI re-read of 100 of them; blinding between the passes was self-reported; 307 records mapped from their abstracts only), so that the 236 charted and 307 mapped records are a broader but still not exhaustive sample of the indexed literature; no formal risk-of-bias assessment; no quantitative synthesis; publication-bias asymmetry.
	21	**Conclusions.** Provide a general interpretation of the results with respect to the review questions and objectives, as well as potential implications and/or next steps.	Sections 9.1 and 9.2, pp. 43–49—Including the specification of an outcome-anchored randomized trial (Figure 10, p. 47) and the sample-size calculations in File S3.
**FUNDING**	22	**Funding.** Describe sources of funding for the included sources of evidence, as well as sources of funding for the scoping review. Describe the role of the funders of the scoping review.	Funding statement, p. 50—This research received no external funding. Developer affiliation and funding of the included sources are characterized in Sections 3 and 8 and flagged at each point of use according to Section 2.4.

Note. Page numbers refer to the second revised manuscript (26 October 2026), as rendered to PDF; they may shift slightly in the journal's template. Items 12 and 16 are optional in PRISMA-ScR and are reported as described in Section 2.5. The flow of records is reported in Figure 2 using the PRISMA 2020 flow diagram template (Page MJ, McKenzie JE, Bossuyt PM, et al. BMJ 2021;372:n71. doi:10.1136/bmj.n71).
