# Supplementary material for: Reproducibility Before Treatment Decision Rules: A Targeted Scoping Review of AI-Assisted Coronary Plaque Characterization
Source: Diagnostics (Basel). 2026 Nov 9;16(21):3703. doi: 10.3390/diagnostics16213703 (PMC13762246; doi:10.3390/diagnostics16213703)
Supplement: Supplementary file 1 [file diagnostics-16-03703-s001.zip › FileS3_Sample-Size_rev2-edited.pdf]

File S3. Sample-size calculations

Reproducibility Before Treatment Decision Rules: A Targeted Scoping Review of AI-Assisted Coronary Plaque Characterization

Inputs are the modeled 10-year relative risk reduction (RRR) and number needed to treat (NNT) reported by FISH&CHIPS [118] (Parsa et al., Am J Prev Cardiol 2026;26:101452), a developer-affiliated modeling study. Absolute risk reduction ARR = 1/NNT; control-arm 10-year event rate p1 = ARR/RRR; intervention-arm rate p2 = p1 − ARR. Two-arm superiority comparison of two proportions, two-sided α = 0.05, power 90%, 1:1 allocation, normal approximation:

n per arm = $[\, z(1-\alpha/2)\cdot\sqrt{2\cdot\bar{p}\cdot(1-\bar{p})} + z(1-\beta)\cdot\sqrt{p1(1-p1) + p2(1-p2)}\,]^2 / (p1 - p2)^2$, with $\bar{p} = (p1+p2)/2$, $z(1-\alpha/2) = 1.95996$ and $z(1-\beta) = 1.28155$ (full-precision standard normal quantiles); n per arm is rounded up to the next whole participant.

Scenario	RRR	NNT	p1 (control)	p2 (intervention)	n per arm	n total
DECIDE stage 3 (enriched design)	24.2%	27	15.3%	11.6%	1,782	3,564
DECIDE stage 1	1.5%	1686	4.0%	3.9%	2,252,348	4,504,696
DECIDE stage 2 (added at the second revision)	18.2%	59	9.3%	7.6%	5,667	11,334
DECIDE stage 4 (added at the second revision)	33.76%	11	26.9%	17.8%	440	880
All stages combined (no enrichment)	19.1%	61	8.6%	6.9%	5,598	11,196

Allowing for 20% attrition, the stage-3 total is inflated by 1/0.8 (≈4450). These figures are a design proposal derived from modeled effects; an observed relative risk reduction may differ from a modeled one. Event rates are 10-year rates for cardiovascular death or non-fatal myocardial infarction as modeled in the source.

Added at the second revision (26 Oct 2026): the rows for DECIDE stage 2 (RRR 18.2%, NNT 59) and stage 4 (RRR 33.76%, NNT 11), computed with the same formula and inputs from

FISH&CHIPS [118], so that every sample size in Figure 9A can be traced here. Stage 3 is 1,782 per arm (3,564 in total), as in Section 9.2.
