# Supplementary material for: Reproducibility Before Treatment Decision Rules: A Targeted Scoping Review of AI-Assisted Coronary Plaque Characterization
Source: Diagnostics (Basel). 2026 Nov 9;16(21):3703. doi: 10.3390/diagnostics16213703 (PMC13762246; doi:10.3390/diagnostics16213703)
Supplement: Supplementary file 1 [file diagnostics-16-03703-s001.zip › FileS4_Figure1-Scoring_rev2-edited.pdf]

File S4 (rev2). Figure 1: scoring rule and cell-by-cell justification

Reproducibility Before Treatment Decision Rules: A Targeted Scoping Review of AI-Assisted Coronary Plaque Characterization

1. The rule

Figure 1 uses the following auditable rule in place of "adequately powered": no formal power calculation was used to define the evidence-map threshold, so the rule uses design properties that can be audited in the chart.

A cell is scored on **AI-derived or automated quantification of that domain's readout**. Evidence from non-AI or semi-automated quantification, or from reviews pooling mixed methods, may be cited in the caption as context. It cannot make a cell filled.

● **FILLED (established).** At least one included primary study at that stage meets all three general criteria and the stage criterion.

- (a) **Design:** Prospective data collection *or* ≥ 2 centers.
- (b) **Size:** ≥ 200 patients. At Stage 1, ≥ 200 patients *or* ≥ 200 lesions or vessels assessed against the reference.
- (c) **Prespecified endpoint.** This is the primary endpoint of a diagnostic study, or an outcome defined and adjudicated prospectively by the parent trial or registry. A post hoc analysis of a prospectively adjudicated outcome qualifies. A cut-off or endpoint derived from the same data does not.
- (d) **Stage criterion:**
- **Stage 1: The reference standard is invasive or histological, as the column header states, and it can measure the readout. IVUS and QCA are adequate for volume and stenosis. Lipid-rich or low-attenuation components need histology, OCT, or NIRS. Agreement with readers, as well as reproducibility, does not count.**
- **Stage 2:** A clinical-event endpoint.
- **Stage 3:** An **observed** change in management or downstream testing. A stated physician intention after unblinded re-review does not count, nor does a modeled change.
- **Stage 4:** Randomized allocation to a strategy using the readout, with a **hard clinical endpoint** (death or MI) as the primary endpoint.

◐ **HALF (limited).** Evidence exists at that stage but none meets FILLED. Typical reasons: single-center and retrospective; fewer than 200; reproducibility only; intention-only or modeled; randomized but with a composite primary endpoint that is not hard.

○ **OPEN.** No included record contributes at that stage.

⊘ **CONTESTED.** Two conditions must both hold.

- The claim is supported by ≥ 1 study of at least HALF grade.
- ≥ 1 **independent** primary study (no developer employees, funding or core-lab role) that itself meets criteria (a) and (b) reaches a **materially different conclusion on the same claim**: a null association, no incremental value or the opposite direction.

A health-technology assessment or review concluding that evidence is *insufficient* is **not** contestation.

Developer marker: superscript "D" on any filled symbol whose qualifying evidence is **all developer-affiliated**. It makes the independence finding of §2.5 visible in the figure without a fifth symbol. Developer-affiliated (D) means that the qualifying evidence comes only from studies whose authors are affiliated with, or funded by, the commercial developer of the platform; software developed by a study's own academic authors is classed as academic in-house and is not marked D.

Rationale for the rule

1. Stage 3 requires an observed change because a stated intention is not one: CERTAIN measures intention after unblinded cross-over. §2.5 classes endpoints as measurement, intention or event, and §5.2 argues that cross-over designs "cannot distinguish better information from anchoring".
2. **Stage 1 requires a reference that can see the readout. Grayscale IVUS validates volume, not lipid core. Without this condition, REVEALPLAQUE's non-calcified-plaque correlation [60] would fill the composition row, which is defined by LAP and LRNC.**
3. **The ≥ 200 threshold is a convention, not a power calculation. It is roughly where a 95% CI on sensitivity or AUC narrows to about ±0.05–0.07. REVEALPLAQUE's own prespecified sample size was 223 [60]. For Stage 2, a stricter alternative would be ≥ 100 events. Applying it would demote Burden 2 ([63] had 55 MACEs; [37] had 87) and Radiomics 2 ([100] had 82 MI), leaving [38] (167 MACEs) as the only qualifying burden or composition study. This stricter rule is reported as a sensitivity option and was not adopted.**

2. Cell-by-cell scoring

Symbols: ● filled · ◐ half-filled · ○ open · ⊘ contested. D: all qualifying evidence is developer-affiliated. The scored dataset is Table S1.

Plaque burden (total plaque volume, PAV)

Stage	Score and references	Justification
1	**● D [60]; limited: [16,52,61]**	[60] REVEALPLAQUE: prospective, 15 centers, 237 patients/432 lesions, blinded IVUS core lab, prespecified sample size. It meets every criterion, but 7/18 authors are HeartFlow employees. The rest fall below the threshold: [61] has 33 patients at one center, [52] has 85 patients/108 vessels, and [16]'s IVUS subset is 50 patients. [66]'s IVUS test-set n is not stated in the abstract.
2	**● [11,37,38,63]; also [16,53,54,70]**	CONFIRM2 [37] (n = 1,916) and [38] (n = 3,551, 18 centers, 167 MACEs), and ADVANCE [63] (n = 4,430, multicenter), all with prospectively adjudicated events. [53] is a single cohort (n = 536). The commercial studies are all developer-sponsored. [16] and [70] use an academic tool whose developers are among

		the authors. [54]: gain "modest"; not significant against vessel count. Added at the second revision: [11] (prospective population-based cohort at six university hospitals, analyses prespecified, n = 23,314, coronary heart disease death or myocardial infarction; total plaque volume quantified with academic software developed by three of its authors [109], with no commercial developer affiliation disclosed) meets the rule. The cell carries no D because studies without commercial developer affiliation meet the rule: [11], and [16] and [70], whose tool is academic in-house (its developers are among the authors; they disclose royalties and equity in a company that licenses plaque-analysis software from their institution, but the sources do not state that this tool is commercialized). The cell would carry no D even if [16] and [70] were counted as developer-affiliated, because [11] qualifies.
3	● ^{D} [27]; also [55,56]	[27] DECIDE registry: prospective, multicenter, 972 patients, prespecified primary endpoint, observed change in management in 51.3%; developer-affiliated (sponsor is the platform manufacturer; several authors are its employees), hence D. [27] was cited at the first revision and is one of the four records added from the confirmatory search (it is not among the 121 records charted at submission; without it the cell is half-filled). Below the bar: [55] CERTAIN passes design and size, but its endpoint is intention after unblinded cross-over; [56]'s reduction in angiography is modeled.
4	○ **none**	[26] AI-DECIDE is a design

		paper with no results, and its abstract does not state randomized allocation.

Plaque composition (LAP, LRNC)

Stage	Score and references	Justification
1	**◐ [33,34]; note [60]**	Carotid histology: [33] has 53 patients (496/408 cross-sections) and [34] has 576 tissue samples with the patient n not stated. Both are developer-affiliated (Elucid). [60] validates the non-calcified split against IVUS (r = 0.87; LAP AUC 0.69–0.72), but IVUS cannot measure LRNC or LAP (rule d).
2	**● [11,38]; non-AI context [3,9] in the caption**	Qualifying studies: [38] and, from the second revision, [11] (below). [38]: AI-QCT non-calcified plaque volume, HR 1.07 per 50 mm^3 as an independent predictor, 18 centers, n = 3,551, Cleerly-sponsored. [3] and [9] are SCOT-HEART semi-automated Autoplaque; [6] is a review pooling manual and AI methods. No AI-derived LAP-specific prognostic study in the included set meets the rule. If the row is read strictly as LAP/LRNC, the cell becomes ◐ with non-AI evidence only. Added at the second revision: [11], the SCAPIS general-population cohort (prospective, six university hospitals, analyses prespecified, n = 23,314, first MI or coronary death; noncalcified plaque volume by QuantiPlaque, developed by three of its authors [109], with no commercial developer affiliation disclosed), meets the rule without developer affiliation, so the D marker is removed. Its analysis centers on total plaque volume; noncalcified plaque volume "yielded similar results".
3	**◐ [55]**	[55] tests the whole AI-QCT report, not composition alone,

		and measures intention.
4	○	No CT-based trial. [125] PREVENT selected plaques by intracoronary imaging; the Figure 1 caption notes this.

Pericoronary inflammation (FAI Score, PCAT attenuation)

Stage	Score and references	Justification
1	**◐ [47]**	The biopsy study has 167 patients, and the reference is adipose-tissue gene expression, not a coronary invasive or histological reference. Developer-affiliated.
2	⊘ [48,49] vs [25]; also [24]	For: [49] ORFAN, prospective, 8 hospitals, prognostic cohort n = 3,393 from 2 hospitals, prespecified endpoints; [48] two centers, n = 3,912 (design corrected against the full text, October 2026). Both are developer-affiliated. Against: [25], an independent three-center cohort (n = 777; no competing interests reported), in which pericoronary attenuation did not improve prediction (a radiomic model of the same fat did); it meets design (a) and size (b), so the cell is contested. [24] RAPID-CTCA (37 sites, n = 353, post hoc; C-statistic 0.57, ΔC 0.00 over LAP) reached the same conclusion, but its disclosure statement could not be accessed at the second revision, so its independence is charted as not stated and it does not contest the cell on its own; at the first revision, the cell was contested by [24], and the 121 records charted at submission alone would score it ●[D] (File S6). Note: [24] and [25] measured raw PCAT attenuation in suspected ACS, not the CaRi-Heart FAI Score in stable chest pain. If Figure 1's claim is "the FAI-Score device", [24] and [25] do not test it, and the cell becomes ●[D] [48,49]. Figure 1 scores the row at class level, as stated in its

		caption.
3	**◐ [50]**	Single center, n = 164, before/after management intention, no outcomes. [76] is a developer-affiliated economic model, not Stage 3.
4	○	—

Radiomic phenotyping

Stage	Score and references	Justification
1	**◐ [97–99]**	[97]: ex vivo, 7 hearts, 95 plaques (445 sections; test set 112), histology. [98] is a post hoc single-center analysis of 25 patients/44 plaques against IVUS, OCT and NaF-PET, with no independent test set. [99]: case-control, 120 patients at one center plus 35 external, with culprit status as the reference. None reaches 200 patients or lesions against an invasive or histological reference.
2	**● [100]**	[100] is a SCOT-HEART post hoc analysis: n = 1,750, multicenter RCT, 82 prospectively adjudicated MI events, C-statistic 0.70 → 0.74. Filled rests on one post hoc study with no external validation. Segmentation was AutoPlaque, with radiomic features on top.
3	○	—
4	○	—

Photon-counting acquisition (UHR PCD-CT measurement)

Stage	Score and references	Justification
1	**◐ [82,83,86]; also [85]**	[82]: 114 patients; the reference is phantom specification only, and it compares reconstructions within PCD-CT. [83]: prospective, single center, 49 patients/278 plaques; invasive QCA covers only 12 patients/56 lesions. [86]: 48 patients/164 plaques, reproducibility only. [85]'s vessel-level accuracy against ICA comes from a retrospective single-center study. None meets the design,

		size and invasive-reference criteria together.
2	○	—
3	**◐ [85]**	Retrospective, single center, non-randomized comparison by scanner, with detector confounded with scanner vendor. Its endpoint (observed referral to ICA, 9.9% vs 13.1%) is Stage 3, but its design fails criterion (a). The legend itself puts single-center evidence at half. [162] and [163] are models.
4	○	—

Addendum (first revision; now incorporated in the table above): plaque burden, stage 3

After this document was drafted, the primary-outcomes publication of the AI-DECIDE program became available (Rinehart et al., JACC Cardiovasc. Imaging 2026;19:706-716; reference [27]). It is prospective, multicenter, enrols 972 patients, has a prespecified primary endpoint and reports an observed change in management in 51.3% of participants rather than a stated intention or a modeled estimate. It therefore meets the criteria for a filled symbol at stage 3, and the plaque-burden stage-3 cell is scored filled, carrying the D marker because the sponsor is the platform manufacturer and several authors are its employees. The earlier references [55,56] remain listed beneath the cell as supporting but non-qualifying evidence: [55] measures intention after unblinded cross-over, and [56] is modeled.

HISTORICAL (first revision, superseded at the second revision; see the Round-2 update). Sensitivity analysis (added at the first revision, October 2026)

The coronary physiology (FFR-CT) domain has been removed from Figure 1 because CT-derived fractional flow reserve falls outside the revised scope of the review. Figure 1 now scores 5 domains across 4 stages, which gives 20 cells. The ≥200-participant threshold in criterion (b) is a convention rather than a power calculation, so Figure 1 is a heuristic evidence map. We re-scored every cell with the threshold at 100 and at 500 participants (at Stage 1, or lesions/vessels). All other criteria and the evidence were left unchanged. The threshold was applied both to studies qualifying a cell and to contesting studies under the ∅ rule. At 100, no cell changes category. At 500, 2 out of 20 cells change. Plaque burden at Stage 1 moves from established to limited ([60]: 237 patients, 432 lesions). Pericoronary inflammation at Stage 2 moves from contested to established, developer-affiliated, because the contesting study [24] (n = 353) no longer meets the size criterion. Both cells are marked † in Figure 1.

HISTORICAL counts (first revision; superseded). Counts (Figure 1 rev2, 5 domains × 4 stages = 20 cells)

Threshold	Cells changing category (of 20)	Cells
≥100	0	none
≥200 (as published)	reference	—
≥500	2	Burden S1 ●^{D} → ◐; Inflammation S2 ∅ → ●^{D}	(Superseded at the second revision: at 500 only one cell changes; see the Round-2 update below.)

Round-2 update (October 2026)

Reference numbers in this file follow the revised manuscript. The coronary physiology (FFR-CT) row has been removed because CT-derived FFR is outside the revised scope; Figure 1 scores 5 domains × 4 stages (20 cells).

At the second revision, the 127 records identified by the re-screen and 4 records added from the confirmatory search were charted, and Figure 1 was re-scored with the rule above (File S6). Relative to the published figure, no cell changed category or D marker at the threshold of 200 participants; relative to the 121 records charted at submission under the corrected charting, two cells changed (plaque burden stage 3 through [27]; pericoronary inflammation stage 2 through [25]); File S6 compares the five versions. Changes to the justifications: Pericoronary inflammation, stage 2: the cell is contested by [25] (independent three-center cohort, n = 777; pericoronary attenuation did not improve prediction) and by [24], whose disclosure statement could not be accessed (charted "not stated" at this revision); at a threshold of 500, the cell therefore remains contested, and its † is removed. Pericoronary inflammation, stage 3: ORFAN [49] also reported a management change in 45% of 744 surveyed patients (details in a supplement not assessed); developer-affiliated; the cell remains half-filled. Plaque burden, stage 3: filled by the DECIDE registry [27], which is now in the charted dataset as one of the four records added from the confirmatory search. Other added records that contribute to a cell are listed in File S6.

Plaque composition, stage 2 (screen of the confirmatory search, 25 October 2026): the SCAPIS general-population cohort [11] was charted from its full text (author decision) and meets the rule: prospective population-based cohort at six university hospitals, analyses prespecified in an analysis plan, 23,314 participants, first myocardial infarction or coronary heart disease death; noncalcified plaque volume was quantified with QuantiPlaque, software developed by three of its authors [109], with no commercial developer affiliation disclosed. Because this qualifying evidence is not developer-affiliated, the D marker is removed (● [11,38]). Its analysis centers on total plaque volume; the authors report that noncalcified plaque volume yielded similar results. The 307 other eligible records from that screen were mapped from their abstracts and changed no cell (File S6).

Current sensitivity analysis (final 236-record dataset; thresholds 100 and 500): at 100, no cell changes; at 500, one cell changes (Plaque burden, stage 1, ●^{D} → ◐; [60] has 237 patients and 432 lesions).

Table S4-K. Key to figure labels

Key from the Author Year labels printed inside Figures 1 to 10 (first author and year of the cited reference; a, b, c or d where first author and year repeat) to the reference numbers of the main-text reference list. The figure captions of the manuscript cite the references already numbered at that point; this table gives the complete key, including references that are first cited later in the text.

Label in figure	Reference number	PMID	Drawn in
Williams 2025	[1]	39863372	Fig 6, Fig 10
Williams 2020	[3]	32174130	Fig 6, Fig 9
Bell 2025	[6]	40425410	Fig 6
Bianchini 2025	[7]	40758074	Fig 6
Malmqvist 2026a	[11]	42663364	Fig 1, Fig 2
Lin 2022a	[16]	35337643	Fig 1, Fig 6, Fig 7
Jonas 2022a	[17]	35986973	Fig 3, Fig 7
Lakshmanan 2026	[24]	41504639	Fig 1, Fig 6, Fig 8
Shang 2025	[25]	40887649	Fig 1

Rinehart 2025	[26]	40673854	Fig 1, Fig 5
Rinehart 2026	[27]	41817483	Fig 1, Fig 2
Tricco 2018	[28]	30178033	Fig 2
Page 2021	[29]	33782057	Fig 2
Sipos 2026	[30]	42155322	Fig 2, Fig 7
Mancini 2024	[31]	38514283	Fig 2, Fig 7
Chandrashekhar 2026	[32]	41405512	Fig 2
Buckler 2023a	[33]	36481054	Fig 1, Fig 2
Buckler 2023b	[34]	36603478	Fig 1, Fig 2
van Rosendael 2026a	[37]	41324522	Fig 1, Fig 8
van Rosendael 2026b	[38]	41906604	Fig 1, Fig 8
Koçak 2025	[43]	39937033	Fig 10
Oikonomou 2019	[47]	31504423	Fig 1, Fig 8
Oikonomou 2021	[48]	34450625	Fig 1, Fig 8
Chan 2024	[49]	38823406	Fig 1, Fig 5, Fig 6, Fig 10
Henry 2024	[50]	40248499	Fig 1, Fig 10
Nakanishi 2026	[52]	41781729	Fig 1
Nurmohamed 2024b	[53]	37480907	Fig 6, Fig 8
Nurmohamed 2024c	[54]	39101625	Fig 8
Nurmohamed 2024d	[55]	38270472	Fig 1, Fig 10
Lipkin 2022	[56]	35441530	Fig 1, Fig 5
Narula 2024	[60]	38700097	Fig 1
Ihdayhid 2024b	[61]	39540820	Fig 1
Dundas 2024	[63]	38469689	Fig 1, Fig 6, Fig 9
Douglas 2023	[64]	37610731	Fig 5, Fig 6, Fig 10
Weichsel 2024	[68]	38248031	Fig 3, Fig 7, Fig 10
Miller 2024	[70]	39405390	Fig 6, Fig 9
Buckler 2024	[75]	39060206	Fig 3, Fig 5, Fig 10
Park 2024a	[79]	38634943	Fig 7
Oberoi 2014	[80]	24370164	Fig 7
Si-Mohamed 2022	[81]	35166583	Fig 4
Halfmann 2024	[82]	38376407	Fig 1, Fig 4
Vecsey-Nagy 2024	[83]	39328060	Fig 1, Fig 4, Fig 9
Tremamunno 2025	[84]	40328542	Fig 4, Fig 9
Sakai 2025	[85]	39466216	Fig 1, Fig 5
Vecsey-Nagy 2025a	[86]	40035676	Fig 1, Fig 3, Fig 4, Fig 7, Fig 9, Fig 10
Zou 2025	[90]	39891682	Fig 7
Vattay 2023	[91]	37488295	Fig 3
Skoog 2025	[92]	40009728	Fig 3
Szilveszter 2026	[93]	42488087	Fig 3
Vecsey-Nagy 2026	[94]	42486966	Fig 5, Fig 7
Maaniitty 2026	[95]	41937634	Fig 5, Fig 9, Fig 10
Schulze 2026	[96]	40751112	Fig 5, Fig 9, Fig 10
Kolossváry 2019a	[97]	31385755	Fig 1
Kolossváry 2019b	[98]	30838375	Fig 1
Lin 2022b	[99]	35512957	Fig 1
Kolossváry 2025	[100]	39480364	Fig 1
Zhu 2025	[103]	39048741	Fig 7
Zhang 2025	[104]	39890616	Fig 7
Kolossváry 2019c	[106]	30447949	Fig 3, Fig 7
Kolossváry 2021	[107]	32868246	Fig 3, Fig 7
Flores Tomasino 2024	[108]	38466392	Fig 7
Malmqvist 2026b	[109]	42135689	Fig 7
Malkasian 2026	[110]	42489557	Fig 7
Jávorszky 2022	[111]	35524783	Fig 7
Ramasamy 2023a	[112]	36270952	Fig 7
Sandoval 2025	[115]	40360362	Fig 5

Kawashima 2020	[116]	33303435	Fig 5
Bolognese 2025	[117]	40248287	Fig 5
Parsa 2026	[118]	42006406	Fig 5, Fig 9, Fig 10
Einstein 2026	[132]	41739468	Fig 10
Hussain 2026	[133]	40835433	Fig 5
Liu 2020	[138]	32909959	Fig 10
Rivera 2020	[139]	32907797	Fig 10
