# Supplementary material for: Reproducibility Before Treatment Decision Rules: A Targeted Scoping Review of AI-Assisted Coronary Plaque Characterization
Source: Diagnostics (Basel). 2026 Nov 9;16(21):3703. doi: 10.3390/diagnostics16213703 (PMC13762246; doi:10.3390/diagnostics16213703)
Supplement: Supplementary file 1 [file diagnostics-16-03703-s001.zip › FileS5_Extended-detail_rev2-edited.pdf]

File S5. Extended platform, regulatory and economic detail

Reproducibility Before Treatment Decision Rules: A Targeted Scoping Review of AI-Assisted Coronary Plaque Characterization

This file holds, verbatim, the detailed text moved from the main text at revision in response to Reviewer 3 (comment 13): the platform-by-platform narrative of Sections 3.2–3.7, the regulatory discussion of Section 6 and the economic detail of Section 7.2. Each main-text section keeps a summary that points here. It is organized by original section number. Bracketed numbers refer to the reference list of the main article; cross-references to sections, figures and tables refer to the main article.

S5.1. Section 3.2. AI-QCT (Cleerly)

The largest published evidence base belongs to Cleerly's atherosclerosis imaging–quantitative CT (AI-QCT); in this review, AI-QCT denotes this platform only, and generic use is written as AI-assisted quantitative CCTA. In a CREDENCE substudy (n = 303), AI-QCT achieved per-patient sensitivity/specificity of 94%/68% for ≥50% stenosis, correlating well with blinded core-lab quantitative coronary angiography (ICC 0.73) [51]. Against intravascular ultrasound (IVUS), the INVICTUS registry reported correlations of 0.83 for plaque volume and 0.94 for lumen volume with independent blinded core-lab reading; it was sponsored by the manufacturer, with the CCTA analysis performed at its own core laboratory, so it is developer-affiliated [52].

Prognostically, AI-QCT plaque staging predicted MACEs over a median 10.3 years (AI-QCT plaque stage 3, defined by percentage atheroma volume: adjusted HR 3.57), improving 10-year AUC over risk factors plus calcium score (0.82 vs. 0.73) [53]. In the CONFIRM2 registry (n = 1916), AI-QCT added incrementally to CAD-RADS 2.0 (AUC 0.81 vs. 0.79; net reclassification index [NRI] 0.47), the calcium score and the modified Duke Index, retaining significance after excluding severe stenosis [37]; a companion analysis (n = 3551, median 4.27 years) identified diameter stenosis and non-calcified plaque volume as the only independent predictors, improving discrimination over a clinical likelihood model from AUC 0.63 to 0.76 [38,39].

Two findings qualify this. In the plaque-staging study, AI-QCT improved 10-year discrimination over both CAD-RADS 2.0 and manual quantitative CT (0.82 versus 0.78 for each; P = 0.023 and P = 0.040), but net reclassification reached significance against neither (0.09, 95% CI −0.02 to 0.29; and 0.04, 95% CI −0.05 to 0.27) [53]. The gain over expert manual quantification is therefore small and, on reclassification, was not demonstrated. In the ISCHEMIA substudy (n = 3711), the authors called the improvement in event prediction "modest" (AUC 0.688 vs. 0.637 over clinical characteristics alone; P = 0.006) in that heavily diseased population; against a model that already included the number of diseased vessels, the further gain from plaque quantification was not significant (0.688 vs. 0.670; P = 0.234) [54].

Downstream effects were examined in CERTAIN, a cross-over study in which physicians re-assessed 750 patients after viewing AI-QCT output: diagnosis or management changed in 57.1%, projected downstream testing fell 37.1%, and statin initiation or intensification rose 28.1% [55]. Because clinicians re-assessed unblinded, this measures stated intent, not realized events. A CREDENCE post hoc analysis reported that AI-QCT outperformed myocardial perfusion imaging (AUC 0.88 vs. 0.66) and modeled a 39–49% reduction in invasive angiography [56]. A CCTA-derived ischemia model combining atherosclerosis and vascular morphology reached AUC 0.80–0.85, outperforming FFR-CT and SPECT but not PET [69]. Age- and sex-specific percentiles have been derived and externally evaluated, with ≥75th percentile total plaque volume associated with MI (unadjusted HR 2.65; adjusted estimates 2.38–2.50); that work used an academic deep-learning system, not this platform [70].

S5.2. Section 3.3. HeartFlow

HeartFlow's most extensive evidence concerns CT-derived FFR: the ADVANCE registry [62,71], a prognostic meta-analysis [72], the PRECISE strategy trial [64] and a real-world audit [73]. FFR-CT is a physiological readout outside the scope of this review, and these studies are cited as context only; this section considers the plaque analysis tool (AI-QCPA).

The plaque tool has been assessed alongside physiology and against invasive imaging. In ADVANCE (n = 4430), AI-enabled total plaque volume >564 mm^3 predicted MACEs or late revascularization incrementally to stenosis and FFR-CT (adjusted HR 1.52) [63]. Against IVUS, the largest prospective validation is REVEALPLAQUE, a 15-center US–Japan study (237 patients, 432 lesions) reporting correlations of 0.91 for total and calcified plaque volume and 0.87 for non-calcified plaque with little Bland–Altman bias [60]; seven of its authors are full-time employees of the manufacturer. A smaller single-center study agreed strongly for total (ρ = 0.92), non-calcified (0.91) and calcified plaque (0.87) but included only 33 patients and 67 vessels and underestimated total plaque volume by 9.4 mm^3 [61].

AI-DECIDE, a manufacturer-funded observational cohort with a projected enrolment of 20,000 (ClinicalTrials.gov NCT06376851) [26,74], has now published the primary outcomes of its pre–post substudy [27]. In 972 symptomatic patients with plaque, release of the AI plaque analysis 90 days after the index CCTA was followed by a change in management in 51.3%, rising with burden to 67.8% in the highest stage; the commonest change was intensification of medical therapy (35.6%), and patients whose management changed had a greater fall in LDL cholesterol (−11 versus 1 mg/dL, P = 0.002). The design is observational, unblinded and without a concurrent control, so it records what clinicians did once they saw the output rather than whether patients benefited, as its authors note in calling for randomized trials.

S5.3. Section 3.4. Elucid

Elucid's distinguishing feature is that tissue definitions are anchored to histology rather than Hounsfield-unit conventions. In a radiologic–pathologic validation study using carotid endarterectomy specimens with paired CTA, machine learning phenotype classification

agreed with expert pathologists (weighted κ 0.82; AUC 0.95–0.99), while diameter stenosis correlated poorly with histological plaque type (weighted κ 0.25) [33]. A companion "virtual pathology" study of 576 tissue samples reported near-unity regression slopes against histology for lipid-rich necrotic core (LRNC), intraplaque hemorrhage, calcification and matrix [34]. In an EVAPORATE substudy the software detected treatment-related LRNC change at 9 months, albeit in 55 patients [57]; an observational psoriasis cohort (n = 209) showed favourable but small LRNC modification with biologic therapy (ΔLRNC −0.22 mm^2) [58]. Reader reproducibility was high in 64 vessels from only 16 patients [59].

As Table 1 shows, the platform with the strongest tissue-level validation in this field has no outcome-anchored evidence identified in the sources searched: no study linking Elucid-derived plaque characterization to MACEs was identified. This is not a criticism of the histological work, the most rigorous analytical validation of any platform. Analytical accuracy against a tissue reference and prognostic performance are separate properties, and the first does not establish the second.

An important Elucid-associated contribution is the consensus QIBA Profile for atherosclerosis biomarkers by CTA, which states that vendors use differing definitions, metrics and validation datasets, leaving clinicians unable to compare accuracy and precision objectively [75], a central structural problem of this product category.

S5.4. Section 3.5. Caristo

Caristo's CaRi-Heart uses the perivascular fat attenuation index (FAI) as a readout of coronary inflammation. The originating work linked CT radiomic features of subcutaneous adipose tissue to adipose gene expression for inflammation, fibrosis and vascularity and then applied the resulting fat radiomic profile to perivascular fat, which improved MACE prediction beyond risk factors, calcium score, stenosis and high-risk plaque features in SCOT-HEART (ΔC-statistic 0.126) [47]. Standardized FAI-Score mapping was then reported as a prognostic medical device in 3912 US and European patients (ΔC-statistic 0.085 and 0.149) [48]. In ORFAN, patients without obstructive CAD accounted for two-thirds of MACE in the full cohort (n = 40,091), whereas the FAI Score was evaluated in a nested cohort of 3393 patients from two hospitals, where elevation in all three coronary arteries carried a hazard ratio for cardiac mortality of 29.8 (95% CI 13.9–63.9), comparing the top with the bottom quartile of the FAI Score in each artery [49]. The effect size and the whole-cohort event distribution therefore come from different populations.

Counter-evidence also exists. An independent NIHR Early Value Assessment identified only one includable study, rated it at high risk of bias by PROBAST, found no studies reporting clinical effects or costs and noted a general lack of evidence that treating on the basis of vascular inflammation improves outcomes [40], the assumption on which a developer-affiliated cost-effectiveness model depends [76]. A single-center series (n = 164) found AI-Risk reclassified about half of patients and changed management in a third, without outcome data [50]. PCAT may also be less robust than early work suggested: in a RAPID-CTCA substudy (n = 353), PCAT attenuation discriminated weakly (C-statistic 0.57) and,

though independently associated with 1-year death or recurrent MI, added no discrimination beyond LAP burden (ΔC-statistic 0.00) [24] (its disclosure statement could not be accessed); a recent review likewise finds only modest additional discrimination in large datasets [77]. A further cohort identified by the recall check (Section 2.3), whose authors include an employee and a founder of the developer, reported independent prognostic value for CaRi-Heart risk [78].

We do not adjudicate between ORFAN and RAPID-CTCA; the discrepancy is unresolved, and developer affiliation differs between them. One asymmetry is relevant. Every other platform in Table 1 is validated against an imaging or histological measure of plaque; CaRi-Heart's originating validation is against adipose gene expression [47]. That is a defensible anchor, but the two studies are not measuring quite the same construct, so comparing effect sizes alone cannot resolve the discrepancy.

S5.5. Section 3.6. Artrya

Artrya-affiliated work reports a fully automated deep learning system whose stenosis evidence is reasonably powered (769 patients, 3012 vessels; 93.5% per-vessel agreement within one CAD-RADS category, per-vessel AUC 0.897 for ≥50% stenosis) but whose high-risk-plaque test set is not (45 patients, AUC 0.77–0.80) [44]. Calcium-scoring [45] and perioperative risk [46] models were validated separately. The product name is absent from indexed abstracts, so attribution rests on author affiliation.

S5.6. Section 3.7. Circle Cardiovascular Imaging

Searching by product and company name (reproduced in File S1) did not identify a peer-reviewed study validating this vendor's coronary plaque quantification against an intravascular, histological or expert reference standard. The software appears in the coronary CT literature as a measurement tool rather than as the object of study: five single-center CCTA studies of plaque quantification, compositional plaque progression and pericoronary adipose tissue used it to derive their plaque measurements without reporting any validation of those measurements against a reference standard, and one of them reports observer reproducibility only (records listed in File S1). We therefore make no claims about its plaque-analysis performance and record, as a finding rather than an omission, that indexed validation was not identified in the sources searched.

S5.7. Section 6. Regulatory and Validation Considerations

The devices in Section 3 are already authorized for clinical use, so what that authorization certifies is part of interpreting their clinical literature.

6.1. The 510(k) Pathway and Predicate Lineage

A scoping review of FDA-authorized AI/ML devices found that 277 out of 1016 (27.3%) had cardiology applications; 97.1% were cleared via the 510(k) pathway, 58.0% were judged at risk of predicate creep, AI technology type was unreported in 58.8%, and only 3.2% were supported by high-quality clinical trials [133].

The structural concern with 510(k) is that clearance rests on substantial equivalence to a predicate. Predicate-network analysis showed more than a third of cleared AI/ML devices originated from non-AI/ML first-generation devices and that the longest-standing cardiovascular AI predicates date to 2008, and the authors raised safety concerns [134]. Earlier work found that only 45% of FDA announcements mentioned AI/ML [135]; a 2024 analysis found Predetermined Change Control Plans in only 16.7% of summaries, with 29.2% reporting both sensitivity and specificity and 15.5% providing demographic data [136]. EU, US and Chinese frameworks are converging on core principles [137], though convergence on principles has not yet produced convergence on the evidentiary threshold for clearance.

6.2. Reporting Standards and Their Uneven Application

Reporting standards exist but are unevenly applied. CONSORT-AI and SPIRIT-AI define trial reporting and protocol requirements [138,139]; an umbrella review of CLAIM adherence spanning 33 reviews and 1458 studies reported, within an 18-review subset (993 studies), a weighted mean compliance of 63% and identified 11 items under-reported in ≥50% of studies [43]. PRIME 2.0 adds cardiovascular-imaging-specific items [140]; FUTURE-AI provides a life-cycle framework [141]; STANDING Together addresses dataset transparency and algorithmic bias [142]; and an NHLBI workshop reached similar conclusions on the translational gap [143]. For quantitative coronary plaque analysis specifically, the 2025 ACC Scientific Statement adds consensus guidance on interpretation, reporting and standardization [32].

The limitation is therefore adherence rather than a lack of standards. Every framework above is voluntary, none is a condition of 510(k) clearance, and the CLAIM compliance figure of 63% [43] indicates that even journal-mediated enforcement is partial.

6.3. Generalizability and External Validation

Generalizability remains a central scientific concern. A scoping review in an adjacent imaging domain found excellent internal performance but external validation in only 7% of studies [144], a pattern the coronary literature partly shares, with exceptions such as multicenter external testing in heavily calcified vessels [67] and multicenter percentile derivation [70]. AI performance for detecting ≥50% stenosis appeared unaffected by scanner vendor, tube voltage, gating technique, beta-blocker or nitroglycerin use, BMI or heart rate; that study addressed stenosis detection rather than plaque quantification [145].

Robustness to acquisition parameters within a platform (Table 3, upper rows) is a different property from agreement between platforms, and the evidence on the second is less favourable [68,75,103].

6.4. What Clearance Does Not Certify

The regulatory and clinical records describe the same gap from two directions. The platforms in Section 3 are in clinical use, yet the fourth column of Figure 1 is empty for every domain. The finding that only 3.2% of authorized cardiology AI devices are supported by high-quality clinical trials [133] is not a separate observation from the finding that no

randomized trial allocating patients to AI-based plaque analysis was identified in the sources searched; it is the same finding seen from the regulatory side.

The practical consequence is that clearance certifies substantial equivalence to a predicate, not analytical comparability between contemporary devices and not clinical benefit. The QIBA Profile [75] attempts to supply this voluntarily by specifying the definitions, metrics and validation datasets that 510(k) equivalence does not require vendors to share. Until such a profile is either widely adopted or required, the non-interchangeability documented in Sections 3.8 and 4.2 is likely to persist as a structural property of the market rather than a temporary immaturity.

S5.8. Section 7.2. The Health-Economic Case

Economic evaluations of CCTA with FFR-CT [158–161], as well as the English real-world FFR-CT audit [73], concern a physiological readout outside the scope of this review and are cited as context only. For AI inflammation analysis, the developer-led model reports strong cost effectiveness [76], while the independent assessment concludes the underlying evidence is insufficient to populate such a model [40].

PCD-CT economics appear more favourable, chiefly through avoided downstream testing: Monte Carlo modeling found 18.9% fewer functional follow-up tests and 6.0% fewer invasive angiograms, with approximately $795 saved per patient over a 10-year horizon [162], and a Finnish analysis found 26.9–33.8% lower downstream costs, breaking even after 4950–7880 patients [163]. Radiation stewardship is also a current equity issue. Across 19,302 patients at 742 centers in 101 countries undergoing non-invasive coronary testing of all kinds, the median effective dose for CCTA specifically was 7.4 mSv; only 56% of the centers performing CCTA met the ≤9 mSv recommendation, and doses were up to 96% higher in low- and lower-middle-income countries [132].
