# Supplementary material for: Reproducibility Before Treatment Decision Rules: A Targeted Scoping Review of AI-Assisted Coronary Plaque Characterization
Source: Diagnostics (Basel). 2026 Nov 9;16(21):3703. doi: 10.3390/diagnostics16213703 (PMC13762246; doi:10.3390/diagnostics16213703)
Supplement: Supplementary file 1 [file diagnostics-16-03703-s001.zip › FileS6_Sensitivity-Analysis_rev3-edited.pdf]

File S6. Sensitivity analysis of the evidence map: the published figure, the 121 records charted at submission and 235, 236 and 543 records after the second revision

Reproducibility Before Treatment Decision Rules: A Targeted Scoping Review of AI-Assisted Coronary Plaque Characterization

1. Purpose and specification

Reviewers asked whether the 127 records identified by the re-screen of records not selected (Section 2.3) could change the evidence map. All 127 were charted at revision; after full charting and the corresponding author's acceptance of AI-recommended decisions (24 October 2026, after he had reviewed the written assessments; not blind to the recommendations), 110 met the eligibility criteria, and 17 did not (Table S1). Four records identified by the confirmatory search and already cited [27,30–32] were added by the authors outside the search pipeline. The merged dataset therefore has 235 records.

The analysis was specified in writing before any added record was scored (protocol file hashed on 21 October 2026). It compares (1) every Figure 1 cell under the unchanged scoring rule of File S4, at the published threshold of 200 participants and at 100 and 500, across five defined versions (Section 2); (2) Table 3 by comparison type; and (3) the platform summaries of Table 1. A change was defined in advance as material if a Figure 1 symbol or developer (D) marker changed, a "none identified" entry in Table 1 became non-empty or an added record contradicted a conclusion in Section 9.1.

Secondary analyses: All 131 added records regardless of eligibility on full charting; only the records that both screening passes had called eligible; and one alternative ruling on a single record (PMID 40831467) as an independent plaque-composition study (the corresponding author ruled it is not a composition readout). Dependence on the four confirmatory-search records is reported separately (Section 2, last paragraph).

2. Figure 1

Domain	Stage	(a) Published Figure 1 (first revision, 9 Oct 2026)	(b) 121 records charted at submission, corrected charting	(c) 235 records (121 + 110 re-screen + 4 confirmatory-search records)	(d) 236 records (+ [11], charted from its full text)	(e) 543 records (+ 307 mapped from abstracts)	Added records contributing to the cell in (c) (round-2 ID: grade)
Plaque burden	Stage 1	●D	●D	●D	●D	●D	N006:HALF<br>N020:HALF<br>N024:HALF

							N043:HALF(dev) N044:HALF(dev) N049:HALF(dev) N072:HALF(dev) N079:HALF(dev) N085:HALF(dev) N106:HALF(dev) N111:HALF(dev) N117:HALF(dev) N119:HALF C2:HALF(dev)
Plaque burden	Stage 2	●	●	●	●	●	N004:HALF N006:HALF N014:HALF N031:FILLED(dev) N054:HALF(dev) N075:HALF(dev) N076:FILLED(dev) N078:HALF(dev) N097:HALF(dev) N101:HALF(dev) N102:HALF N104:HALF(dev) N105:FILLED(dev) N107:HALF(dev) N126:HALF(dev)
Plaque burden	Stage 3	●[D]	◐	●[D]	●[D]	●[D]	N031:HALF(dev) C1:FILLED(dev)
Plaque burden	Stage 4	○	○	○	○	○	—

Plaque composition	Stage 1	◐	◐	◐	◐	◐	N006:HALF N016:HALF N020:HALF N024:HALF N042:HALF N043:HALF(dev) N044:HALF(dev) N066:HALF(dev) N072:HALF(dev) N074:HALF N079:HALF(dev) N085:HALF(dev) N087:HALF N111:HALF(dev) N116:HALF N119:HALF C3:HALF(dev)
Plaque composition	Stage 2	●[D]	●[D]	●[D]	●	●	N004:HALF N006:HALF N014:HALF N035:HALF N036:HALF N054:HALF(dev) N075:HALF(dev) N076:FILLED(dev) N080:HALF(dev) N097:HALF(dev) N101:HALF(dev) N102:HALF N126:HALF(dev) N127:HALF(dev)
Plaque composition	Stage 3	◐	◐	◐	◐	◐	—
Plaque composition	Stage 4	○	○	○	○	○	—
Pericoronary	Stage 1	◐	◐	◐	◐	◐	N025:HALF

inflammation							
Pericoronary inflammation	Stage 2	∅	●[D]	∅	∅	∅	N036:HALF N063:HALF(dev) N091:HALF N096:HALF
Pericoronary inflammation	Stage 3	◐	◐	◐	◐	◐	—
Pericoronary inflammation	Stage 4	○	○	○	○	○	—
Radiomic phenotyping	Stage 1	◐	◐	◐	◐	◐	N038:HALF N066:HALF(dev)
Radiomic phenotyping	Stage 2	●	●	●	●	●	N005:HALF N035:HALF N091:HALF
Radiomic phenotyping	Stage 3	○	○	○	○	○	—
Radiomic phenotyping	Stage 4	○	○	○	○	○	—
Photon-counting acquisition	Stage 1	◐	◐	◐	◐	◐	N055:HALF(dev) N061:HALF(dev) N093:HALF(dev) C2:HALF(dev)
Photon-counting acquisition	Stage 2	○	○	○	○	○	—
Photon-counting acquisition	Stage 3	◐	◐	◐	◐	◐	N093:HALF(dev)
Photon-counting acquisition	Stage 4	○	○	○	○	○	—

All symbols are at the published threshold of 200 participants. At 100 and 500 participants, the only cells whose symbol differs from the 200 column in any version are as follows:

Cell	Threshold	(a)	(b)	(c) 235	(d) 236	(e) 543
Plaque burden, stage 1	500	◐	◐	◐	◐	◐

Pericoronary inflammation, stage 2	500	●^{D}	●^{D}	∅	∅	∅

Result. Definitions: (a) is the figure as published at the first revision; (b) applies the same rule to the 121 records charted at submission, under the charting corrected at this revision (cell-level evidence from File S4; membership and independence from Table S1); and (c) to (e) add the records charted at this revision, scored by the same scoring functions with (b) as the starting point. Relative to the published figure (a), the 235 records (c) changed no cell or developer marker at the published threshold of 200 participants, in the main analysis and in every secondary analysis except the alternative ruling on PMID 40831467 (composition, stage 2, would lose its D marker; the corresponding author ruled that this multi-input risk model is not a plaque-composition readout). Relative to the corrected 121-record baseline (b), two cells changed at 200: plaque burden at stage 3, from limited (◐) to established and developer-affiliated (●^{D}), through the DECIDE registry [27], one of the four confirmatory-search records; and pericoronary inflammation at stage 2, from established and developer-affiliated (●^{D}) to contested (∅), through [25] (round-2 record N091; independent three-center cohort of 777 patients; pericoronary attenuation did not improve prediction). The two cells differ between (a) and (b) for these reasons: in (a), the stage-3 burden cell already rested on [27], which was cited in the first revision, and the inflammation cell was contested by [24], whose disclosure statement could not be accessed at this revision, so its independence is charted as "not stated", and it can no longer contest the cell. At a threshold of 500, the inflammation cell is contested in (c) to (e) through [25]; the published figure marked it † for changing to established at that threshold. Adding [11] (d) removed the developer marker on plaque composition at stage 2; the 307 mapped records (e) changed nothing further (Section 5). Figure 1 in the revised manuscript shows (d), which is identical to (e). Round-2 IDs map to PMIDs in Table S1.

Dependence on the confirmatory-search records: the burden stage-3 cell is filled only by the DECIDE registry [27], one of the four records added from the confirmatory search; without [27], it is half-filled, resting on stated intention [55] and modeling [56], as in (b). No other cell depends on the four records.

3. Table 3

Round-2 ID/PMID	Comparison type	Row as extracted	Independence
N020/35524783	Human-versus-AI agreement	human-versus-AI agreement | DL vs. expert manual segmentation | total, noncalcified, calcified, low-attenuation plaque volume | ICC 0.88, 0.84, 0.99, 0.25 (test set)	Academic in-house software; developers among the study authors
N020/35524783	Inter-scanner and detector	inter-scanner and detector	Academic in-house software;

	reproducibility	reproducibility | external site/different machinery and plaque characteristics | total, NCP, CP, LAP volume | ICC 0.62/0.54/0.91/0.48 before and 0.94/0.93/0.95/0.86 after transfer learning	developers among the study authors
N025/36266205	Inter-scanner and detector reproducibility	inter-scanner and detector reproducibility | imaging parameters (tube voltage, pixel spacing and heart rate) and patient characteristics | PCAT attenuation and gradient | not reproducible across parameters: 120 kVp +8 HU, pixel spacing +32 HU (p<.001); plaque–PCAT associations lost after adjustment	independent
N026/36270952	Inter-scanner and detector reproducibility	inter-scanner and detector reproducibility | reconstruction settings (kernel, slice thickness 0.75 vs. 0.50 mm, iterative reconstruction strength) vs. IVUS | total atheroma volume, PAV, MLA, plaque burden | best approach TAV ICC 0.91, PAV ICC 0.64, plaque burden ICC 0.45	n.a.
N049/38630211	Human-versus-AI agreement	human-versus-AI agreement | AI-QCT plaque-volume stage vs. expert-consensus semi-	developer-affiliated

		quantitative/visual methods | total plaque burden category | 93% (k=0.87) vs. SIS; 64.4% (k=0.488) vs. visual; 66.3% (k=0.488) vs. CACS	
N055/38882632	Inter-scanner and detector reproducibility	inter-scanner and detector reproducibility | reconstruction (standard vs. ZeeFree stair-step artefact correction) on PCD-CT | total, calcific, non-calcific, fibrotic and lipidic plaque volume | not interchangeable: total 111±60 vs. 75±50 mm3 (p<0.001)	partial
N061/39321385	Inter-scanner and detector reproducibility	inter-scanner and detector reproducibility | detector type (simulated deep-silicon PCD vs. EID), voxel size/kernel, noise and motion | necrotic core area, fibrous cap thickness | PCD errors lower (necrotic core area error 24% vs. 91.5%; fibrous cap thickness 33.3% vs. 349.8%)	developer-affiliated
N069/39891682	Inter-scanner and detector reproducibility	inter-scanner and detector reproducibility | reconstruction algorithm and dose (LD HIR vs. ULD SR-DLR, same scanner) | necrotic, fibrous and calcified plaque volumes | ICC 0.909/0.973/0.980	developer-affiliated
N072/40019592	Human-versus-AI agreement	human-versus-AI agreement | reader	developer-affiliated

		CAD-RADS/CAC category vs. AI-QCT | total and non-calcified plaque volume | stepwise TPV increase across reader categories; AUC 0.86 for TPV <12.3 mm3 vs. reader NORMAL; AI found no plaque in only 26.0% of reader-normal	
N091/40887649	Inter-reader reproducibility	inter-reader reproducibility | two radiologists | culprit PCAT radiomic features | features with ICC > 0.9 retained (individual ICC values not reported in main text)	independent
N111/41549197	Human-versus-AI agreement	human-versus-AI agreement | AI-QCT vs. clinical visual reading | detection of non-obstructive plaque | substantial disagreement (non-obstructive 88.5%/85.6% AI-QCT vs. 16.7%/11.6% clinical); obstructive stenosis comparable	developer-affiliated
N116/41940098	Human-versus-AI agreement	human-versus-AI agreement | DL calcium-scoring tool vs. five expert cardiologists | calcification score | ICC 0.84	Academic in-house software; developers among the study authors
N116/41940098	Human-versus-AI agreement	human-versus-AI agreement | DL vs. expert visual calcification scoring | calcification score | kappa 0.76	Academic in-house software; developers among the study authors
N119/42135689	Human-versus-AI agreement	human-versus-AI agreement |	Academic in-house software;

		algorithm vs. single expert reader | total and calcified plaque volume | TPV ICC 0.95, bias -8.35 mm3, LoA -102 to 85 mm3; CPV ICC 0.90	developers among the study authors
N119/42135689	Inter-reader reproducibility	inter-reader reproducibility | two expert readers (subset) | total plaque volume | ICC 0.98, LoA -73 to 82 mm3	Academic in-house software; developers among the study authors
N122/42209864	Inter-reader reproducibility	inter-reader reproducibility | core-laboratory readers | plaque volumes | not stated in main text (Supplemental Table 1)	Academic in-house software; developers among the study authors
N127/42489557	Inter-reader reproducibility	inter-reader reproducibility | two readers on 12 randomly selected lesions | visual LAP shape/embeddedness (ALM) on AI-QCT output | κ = 0.67 (substantial)	developer-affiliated

Result: the added records contained no further inter-vendor (between-platform) comparison. Rows that met the Table 3 definition were added to Table 3 and marked †; they repeat the published pattern (close agreement for total plaque volume, poor agreement for low-attenuation plaque and reconstruction- and parameter-dependent values).

4. Platform summaries (Table 1)

Added records by platform: of the 115 records charted at revision, a record is counted for a vendor when Table S1 attributes it to that vendor at any level of the attribution hierarchy (Section 2.4); records the source leaves unattributed are not counted. AI-QCT (Cleerly) 30 (24 developer-affiliated, 3 partial, 2 independent and 1 not stated; neither independent record is a prognostic, decision-impact or randomized study); HeartFlow 5 and Caristo 6 (all developer-affiliated); Elucid 2 (no prognostic study); Artrya and Circle none. Pericoronary-fat software from one further vendor (Shukun) was used in three independent prognostic studies. No added record reported a randomized outcome trial.

Correction made at revision: a cohort previously described as not evidently affiliated with the developer of CaRi-Heart (reference [78]) lists a developer employee and the developer's founder among its authors; Sections 3.5 and 8 were corrected.

Conclusion: The added records support the existing findings and do not change the Figure 1 classes, the Table 3 picture or the conclusions in Section 9.1.

Method note: Extraction, reconciliation and scoring were performed by AI agents (Section 2.7); eligibility questions and judgement items were decided by the corresponding author on 24 October 2026. Scripts and hashed outputs are retained by the authors.

5. Records from the screened confirmatory search (second revision)

Data. The 3,634 records of the confirmatory search of 15 September 2026 were screened by two independent AI passes with author decisions on unresolved records (main text Section 2.3; File S7). In total, 314 records were eligible: 6 were already charted ([33], [34] and [27,30–32]), and 308 were new. One new record, the SCAPIS general-population cohort [11], was charted from its full text (author decision, 25 October 2026); it is row [11] of Table S1. The other 307 were mapped from their abstracts and PubMed disclosure fields on the fields Figure 1 scoring uses, not fully charted (author decision, 25 October 2026). Each mapped value was extracted by two independent AI sessions; every disagreement was resolved against the abstract with a verbatim quote (File S7, sheet B).

Method. The File S4 scoring rule and thresholds (100, 200, 500) were applied without change, by copies of the scoring scripts whose scoring functions are byte-identical to the originals, to the 235 records charted before this screen, to those records plus [11] (236) and to 236 + 307 = 543 records, and, as a secondary analysis, with only the 238 mapped records that both passes included.

Domain	Stage	(c) 235 charted @100/200/500	(d) 236 charted @100/200/500	(e) 236 charted + 307 mapped (543) @100/200/500
burden	S1	●[D] / ●[D] / ◐	●[D] / ●[D] / ◐	●[D] / ●[D] / ◐
burden	S2	● / ● / ●	● / ● / ●	● / ● / ●
burden	S3	●[D] / ●[D] / ●[D]	●[D] / ●[D] / ●[D]	●[D] / ●[D] / ●[D]
burden	S4	○ / ○ / ○	○ / ○ / ○	○ / ○ / ○
composition	S1	◐ / ◐ / ◐	◐ / ◐ / ◐	◐ / ◐ / ◐
composition	S2	●[D] / ●[D] / ●[D]	● / ● / ●	● / ● / ●
composition	S3	◐ / ◐ / ◐	◐ / ◐ / ◐	◐ / ◐ / ◐
composition	S4	○ / ○ / ○	○ / ○ / ○	○ / ○ / ○
inflammation	S1	◐ / ◐ / ◐	◐ / ◐ / ◐	◐ / ◐ / ◐
inflammation	S2	⊘ / ⊘ / ⊘	⊘ / ⊘ / ⊘	⊘ / ⊘ / ⊘
inflammation	S3	◐ / ◐ / ◐	◐ / ◐ / ◐	◐ / ◐ / ◐
inflammation	S4	○ / ○ / ○	○ / ○ / ○	○ / ○ / ○
radiomics	S1	◐ / ◐ / ◐	◐ / ◐ / ◐	◐ / ◐ / ◐
radiomics	S2	● / ● / ●	● / ● / ●	● / ● / ●
radiomics	S3	○ / ○ / ○	○ / ○ / ○	○ / ○ / ○
radiomics	S4	○ / ○ / ○	○ / ○ / ○	○ / ○ / ○
pcd	S1	◐ / ◐ / ◐	◐ / ◐ / ◐	◐ / ◐ / ◐

pcd	S2	○ / ○ / ○	○ / ○ / ○	○ / ○ / ○
pcd	S3	◐ / ◐ / ◐	◐ / ◐ / ◐	◐ / ◐ / ◐
pcd	S4	○ / ○ / ○	○ / ○ / ○	○ / ○ / ○

Result. Relative to the 235 records (c), one cell changed: plaque composition at stage 2 lost its developer marker (●^{D} → ●) at every threshold because [11] meets the rule (prospective population-based cohort at six university hospitals; analyses prespecified in an analysis plan; 23,314 participants; clinical-event endpoint; noncalcified plaque volume quantified with QuantiPlaque, software developed by three of its authors [109], with no commercial developer affiliation disclosed) and is not developer-affiliated in the commercial sense (academic in-house software, as for [16] and [70]). The published analysis centers on total plaque volume; the authors report that replacing it with noncalcified plaque volume "yielded similar results". The 307 mapped records changed no cell or developer marker, with all of them or with only those both passes included. They contained no randomized outcome study, no observed decision-impact study and no photon-counting prognostic study.

Limitation. Abstracts rarely state a prespecified endpoint or the number of centers. Upper-bound runs (UB1: unstated prospective/multicenter design and prespecified endpoint set to met; UB2: additionally, unstated independence set to independent) identify one further cell that full texts could change at 200 participants: radiomics stage 1 (◐→●), through PMID 35171348 (546 pathology samples) and PMID 36786701 (708 patients); neither full text could be retrieved through open-access interfaces (Europe PMC, NCBI PMC, Unpaywall-listed locations; no bypassing of publisher access controls). Composition stage 1 could change only at 100. Two histology validations first identified as composition stage-1 candidates are already charted as [33] and [34]; their full texts (supplied by the corresponding author) do not meet the stage-1 bar (53 carotid endarterectomy patients; the 576 samples and 904 cross-sections are sections, not lesions; no prespecified endpoint). Other full-text checks: PMID 42366355 (PMC13573479) is single-center and retrospective (no change); PMID 40161391 (PMC11951307) states no prespecified endpoint.

Changes to the text. One further between-platform comparison of plaque measurement (PMID 38634943, main text [79]; confirmed in its open-access full text, PMC11213768) is added to Table 3 (row marked ‡). One further method comparison concerns pericoronary fat rather than plaque: PMID 42606570 compared an AI tool for the fat attenuation index with workstation measurement on the same examinations of 1,125 patients from five scanners (ρ 0.877; mean bias 1.60 HU), with a developer affiliation inferred from an author affiliation (abstract only). The mapped records contribute 41 candidate reproducibility rows (inter-scanner and detector 24, human-versus-AI 7, intra-algorithm 5, inter-reader 4 and inter-vendor 1); only the inter-vendor row is added to Table 3 because the mapped records are reported at the abstract level. Ref. [11] is also cited in Sections 1 and 9.1 as evidence of improved prediction in a general population, not of treatment benefit.

Method note. Extraction, resolution, full-text charting of [11] and scoring were performed by AI agents; the author did not check values field by field.
